# Effects of sleep and wake on astrocytes: clues from molecular and ultrastructural studies

**DOI:** 10.1186/s12915-015-0176-7

**Published:** 2015-08-25

**Authors:** Michele Bellesi, Luisa de Vivo, Giulio Tononi, Chiara Cirelli

**Affiliations:** Department of Psychiatry, University of Wisconsin–Madison, 6001 Research Park Blvd, Madison, WI 53719 USA

**Keywords:** Mouse, Forebrain, Sleep, Wake, Sleep deprivation, bacTRAP

## Abstract

**Background:**

Astrocytes can mediate neurovascular coupling, modulate neuronal excitability, and promote synaptic maturation and remodeling. All these functions are likely to be modulated by the sleep/wake cycle, because brain metabolism, neuronal activity and synaptic turnover change as a function of behavioral state. Yet, little is known about the effects of sleep and wake on astrocytes.

**Results:**

Here we show that sleep and wake strongly affect both astrocytic gene expression and ultrastructure in the mouse brain. Using translating ribosome affinity purification technology and microarrays, we find that 1.4 % of all astrocytic transcripts in the forebrain are dependent on state (three groups, sleep, wake, short sleep deprivation; six mice per group). Sleep upregulates a few select genes, like *Cirp* and *Uba1*, whereas wake upregulates many genes related to metabolism, the extracellular matrix and cytoskeleton, including *Trio*, *Synj2* and *Gem*, which are involved in the elongation of peripheral astrocytic processes. Using serial block face scanning electron microscopy (three groups, sleep, short sleep deprivation, chronic sleep restriction; three mice per group, >100 spines per mouse, 3D), we find that a few hours of wake are sufficient to bring astrocytic processes closer to the synaptic cleft, while chronic sleep restriction also extends the overall astrocytic coverage of the synapse, including at the axon–spine interface, and increases the available astrocytic surface in the neuropil.

**Conclusions:**

Wake-related changes likely reflect an increased need for glutamate clearance, and are consistent with an overall increase in synaptic strength when sleep is prevented. The reduced astrocytic coverage during sleep, instead, may favor glutamate spillover, thus promoting neuronal synchronization during non-rapid eye movement sleep.

**Electronic supplementary material:**

The online version of this article (doi:10.1186/s12915-015-0176-7) contains supplementary material, which is available to authorized users.

## Background

In addition to clearing neurotransmitters and maintaining ionic balance, astrocytes can mediate neurovascular coupling, modulate neuronal excitability, and promote synaptic formation and remodeling [1–4]. These functions are likely to be modulated by the sleep/wake cycle, but as yet little is known about the effects of sleep/wake on astrocyte functions. Several experiments focused on glycogen, which in the brain is mainly present in astrocytes, testing the hypotheses that sleep serves to restore glycogen deposits or that glycogen levels during wake affect sleep need. However, it was found that the overall effect of wake is to increase glycogen turnover rather than to cause its depletion, providing little support to the original hypotheses [5]. On the other hand, astrocytes can affect sleep. Intracellular recordings of neurons and glia under slow-wave anesthesia show that by buffering K^+^ and Ca^++^ fluxes, astrocytes modulate the timing of the on and off phases of the slow oscillation [6, 7], the fundamental cellular phenomenon that underlies non-rapid eye movement (NREM) sleep [8]. Moreover, dnSNARE mice, in which gliotransmission is blocked, show a blunted homeostatic increase in sleep slow waves after sleep deprivation [9], although new evidence shows that the dnSNARE transgene is also expressed in cortical neurons [10]. Finally, extracellular cortical levels of lactate and glutamate increase with wake and decrease with NREM sleep in a manner that reflects sleep depth [11, 12], suggesting that glycolytic activity in astrocytes, likely through its link with glutamatergic transmission, reflects sleep homeostasis.

Here we first employed translating ribosome affinity purification (TRAP) technology combined with microarray analysis to obtain a genome-wide sleep/wake profiling of astrocytic transcripts in the mouse forebrain. The expression of an eGFP-L10a ribosomal transgene was targeted to cells expressing 10-formyltetrahydrofolate dehydrogenase (ALDH1L1), with the goal of tagging polysomes and immunoaffinity purify mRNAs [13]. Although there is no comprehensive and entirely specific molecular marker for astrocytes [2], ALDH1L1 is more inclusive than glial fibrillary acidic protein (GFAP), and its expression is not limited to specific cortical areas or layers [14]. As a result, we isolated astrocytic mRNAs attached to ribosomes, and thus presumably poised to become proteins. We then used serial block face scanning electron microscopy (SBF-SEM) [15] to test whether sleep, acute sleep deprivation and chronic sleep restriction affect the ultrastructure of astrocytic processes contacting cortical spines. Both sleep and astrocytes affect synaptic plasticity, and sleep need increases not only with the duration of wake, but also with the extent of the plastic changes that occur during wake [16]. We focused on layer II of the prefrontal cortex, because supragranular layers are highly plastic [17] and the slow-wave activity (SWA) of NREM sleep, which reflects the need for sleep and its depth, is highest in frontal areas [18].

## Results

### Molecular studies

**Fig. 1:**
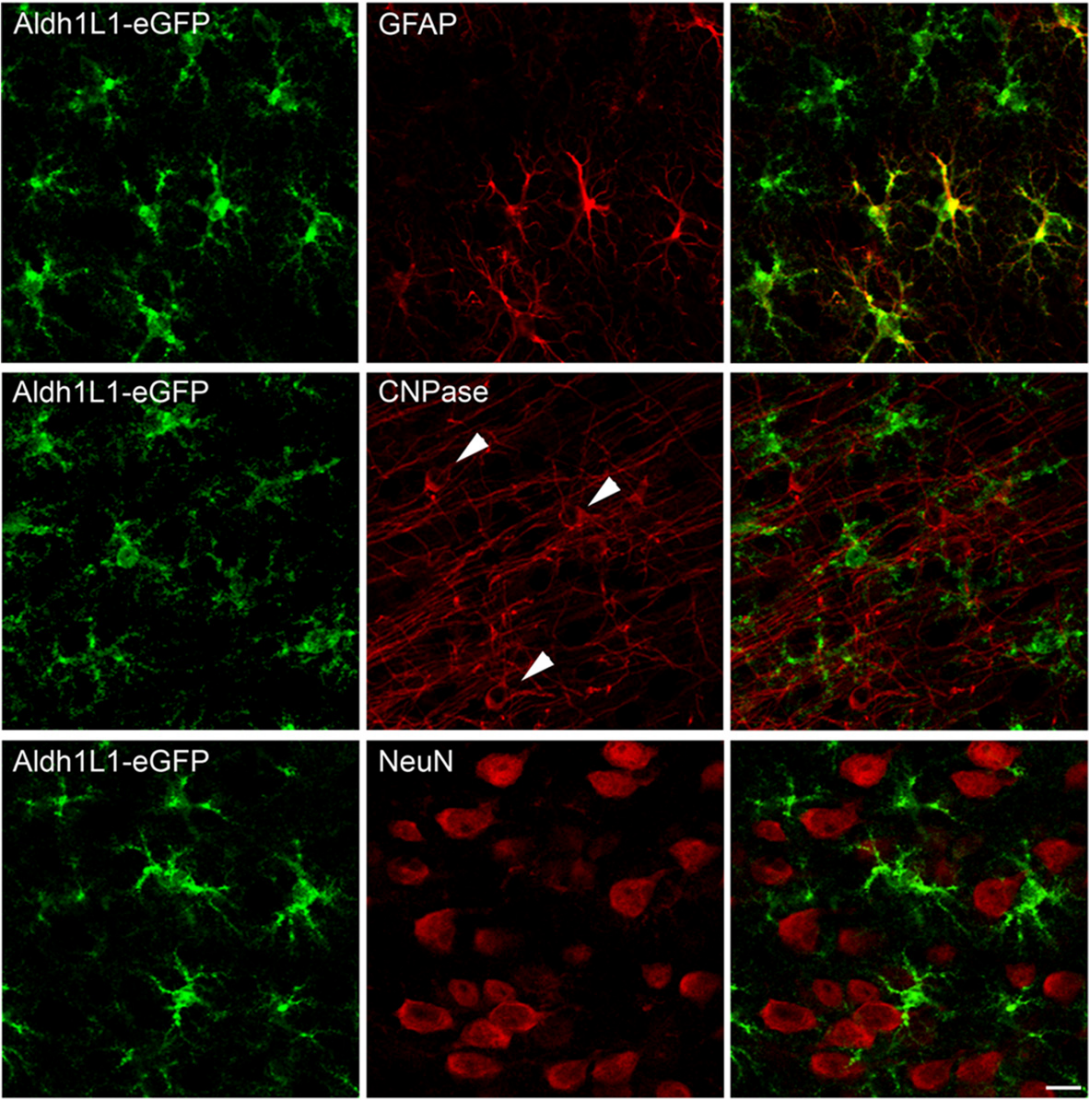
ALDH1L1-eGFP expression is specific for astrocytes. Top panels: Double-labeling studies showing colocalization of ALDH1L1-eGFP (*green*) and the astrocytic marker GFAP (*red*). ALDH1L1 is expressed also in GFAP– astrocytes. Middle and bottom panels: Double-labeling studies showing the absence of colocalization between ALDH1L1-eGFP (*green*) and the oligodendrocyte marker CNP (*red*, *arrows* indicate cell bodies) or the neuronal marker NeuN (*red*). *Scale bar* = 15 μm

The brains of three adult heterozygous ALDH1L1 – eGFP-L10a bacterial artificial chromosome (BAC) transgenic mice were processed for immunocytochemistry using antibodies against enhanced green fluorescent protein (eGFP), an astrocytic marker (anti-GFAP), an oligodendrocytic marker (CNP), and a neuronal marker (anti-NeuN). GFP immunofluorescence was restricted to non-neuronal, non-oligodendrocytic cells (Fig. 1), consistent with previous evidence [13]. Some GFP-labeled cells did not express GFAP, also in line with published data [13, 19, 20]. Thus, these results confirm that ALDH1L1 is a more inclusive astrocytic marker than GFAP.

#### Sleep/wake pattern and response to sleep deprivation are normal in ALDH1L1 – eGFP-L10a mice

**Fig. 2:**
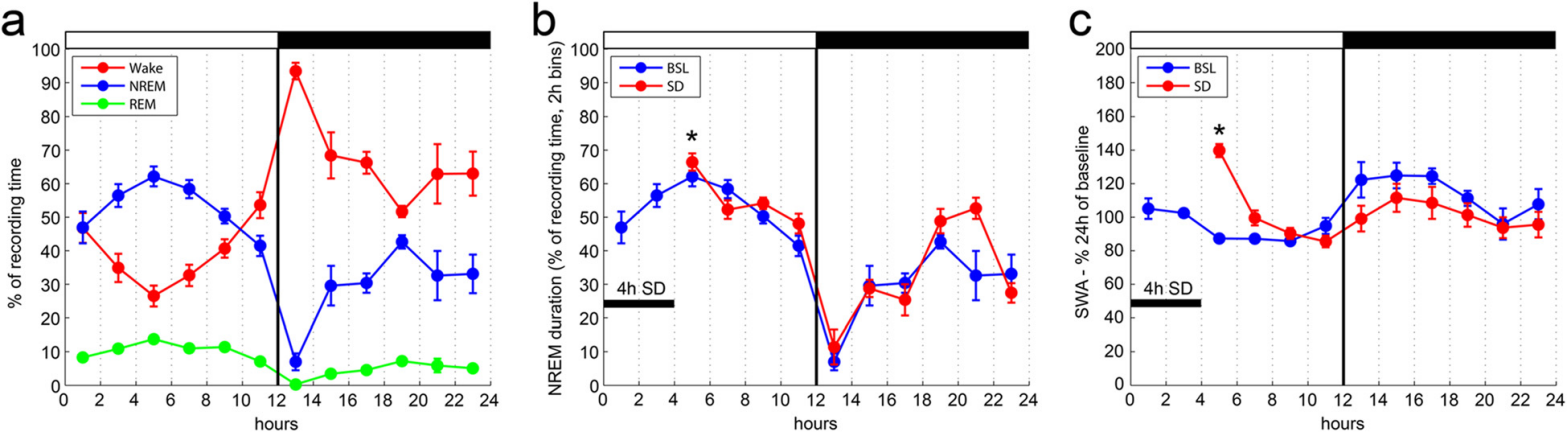
Sleep/wake pattern and response to sleep deprivation in ALDH1L1 – eGFP-L10a mice. **a** Twenty-four hour sleep/wake patterns. **b**, **c** Twenty-four hour time course of NREM duration and SWA for baseline (BSL) and sleep deprivation (SD). *P < 0.05, significant increase during the first 2 hr of recovery sleep after SD relative to the first 2 hr of BSL (paired *t* test). For a-c, values are mean ± standard error of the mean. *White* and *black bars* indicate the light and dark periods, respectively

We first tested whether ALDH1L1 – eGFP-L10a mice showed a normal sleep/wake cycle by making chronic electroencephalographic (EEG) recordings that lasted for several days, including during and after sleep deprivation. Mice (*n* = 5) showed robust entrainment to the light–dark cycle, sleeping mainly during the day and staying awake most of the night, as expected for nocturnal animals. During baseline, NREM sleep accounted for 39.2 ± 1.1 % of the 24-hr cycle, and 50.4 ± 2.3 % of the light period, while the corresponding values for rapid eye movement (REM) sleep were 7.4 ± 0.4 % and 10.4 ± 0.4 % (Fig. 2a). As expected, NREM sleep was predominant in the first part of the light phase relative to the second part (Fig. 2a). The mean duration of NREM and REM episodes, number of brief arousals, and several other sleep parameters were all within the range reported for most mouse lines (e.g. [21–24]).

In mammals, SWA – the EEG power between 0.5 and 4.5 Hz – reflects both the need for sleep and its depth [18]. Indeed, SWA levels increase as a function of prior wake duration and decline in the course of sleep. Moreover, arousal thresholds are higher when SWA is higher. Changes in SWA were studied after ALDH1L1 – eGFP-L10a mice were deprived of sleep during the first 4 hr of the light period. During sleep deprivation, they spent less than 2 % of their time in NREM sleep, and REM sleep was completely abolished. In the first 2 hr of recovery sleep, NREM sleep duration was significantly increased relative to baseline (Fig. 2b), while REM sleep and total sleep amounts did not change (not shown). SWA during the first hour of recovery sleep was increased relative to baseline levels in both the frontal (Fig. 2c) and parietal cortices (not shown). Thus, we conclude that sleep patterns and mechanisms of sleep regulation in ALDH1L1 – eGFP-L10a mice are normal.

#### TRAP enriches for astrocytic-specific genes

Three groups of mice were used in the TRAP study (*n* = 6 per group): awake mice (W) were collected during the dark phase (~3–5 am) at the end of a long period of wake (>1 hr, interrupted by periods of sleep of <5 min), and after spending at least 70 % of the previous 6–7 hr awake. Sleeping mice (S) were collected during the light period (~3–5 pm), at the end of a long period of sleep (>45 min, interrupted by periods of wake of <4 min), and after spending at least 75 % of the previous 6–7 hr asleep. Sleep-deprived mice (SD) were spontaneously awake during most of the dark phase and then kept awake during the first 4 hr of the light period by exposure to novel objects and by tapping on the cage when slow waves appeared on the EEG.

**Fig. 3:**
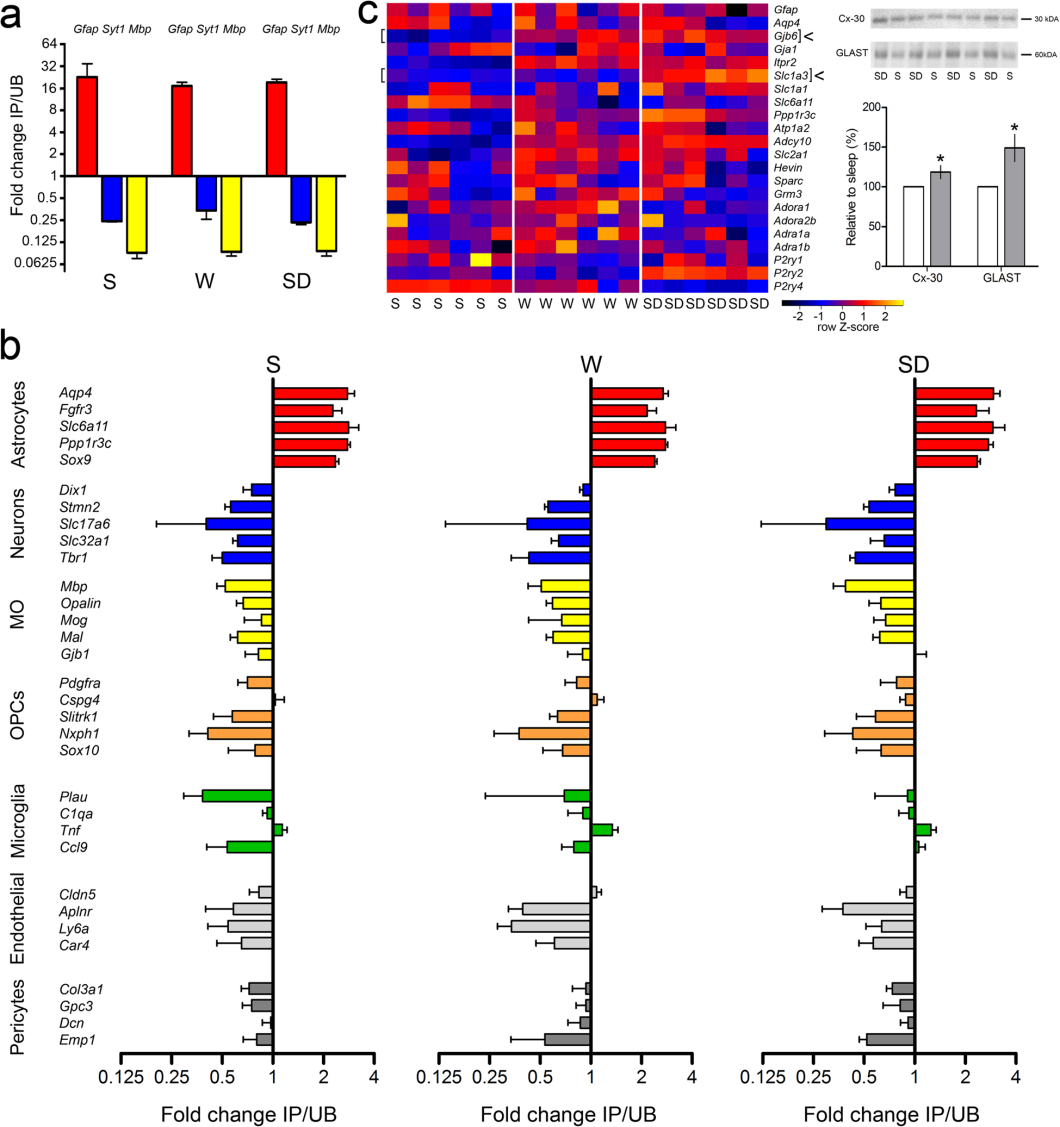
Enrichment analysis of ALDH1L1 – eGFP-L10a IP samples. **a** qPCR expression (mean ± standard deviation, *n* = 6, two per group for IP, *n* = 6, two per group for UB) of the cell-specific marker for astrocytes (*Gfap*) is consistently enriched in the IP RNA across all groups (S, W and SD), whereas the negative controls (*Mbp* for oligodendrocytes, *Syt1* for neurons) are consistently enriched in the UB samples. **b** Histograms showing IP/UB ratios expressed in log_2_ fold change for S, W and SD samples. In all three experimental groups, the genes identified by [25] as specific for astrocytes (*red*) are enriched in IP samples, whereas the genes specific for neurons (*blue*), mature oligodendrocytes (*MO*, *yellow*), oligodendrocyte precursor cells (*OPCs*, *orange*), microglia (*green*), endothelial cells (*light grey*) and pericytes (*dark grey*) are enriched in S, W and SD UB samples. **c** Heat diagram (*left*) showing the expression intensities of several common astrocytic genes in each individual S, W and SD mouse. *Arrows* indicate *gjb6* and *slc1a3*, the two astrocytic genes selected for Western blot analysis (*right*): Cx-30 (+19 ± 8 %, Mann–Whitney (MW), *P* = 0.02), GLAST (+49 ± 17 %, MW, *P* = 0.02) in SD (*n* = 4, *grey bars*) relative to S (*n* = 4, *white bars*). Representative bands from S and SD pools (*n* = 4 mice per pool) are depicted above each bar for Cx-30 and GLAST

We checked whether TRAP accurately enriched for astrocytic-specific genes using two approaches. First, we randomly selected six astrocytic immunoprecipitated samples (IP) and six unbound samples (UB) from the S, W and SD groups (two mice per group) and performed quantitative polymerase chain reaction (qPCR) for the genes coding for the astrocytic marker GFAP, the neuronal marker SYT1 and the oligodendrocyte marker MBP. In all mice, GFAP expression was markedly higher in the IP sample relative to the UB sample, while the opposite was true for MBP and SYT1 (Fig. 3a). We then used the TRAP microarray data to calculate the IP/UB ratio for genes previously identified as enriched in astrocytes, neurons, mature oligodendrocytes (MOs), oligodendrocyte precursor cells (OPCs), microglia, endothelial cells or pericytes [25]. Astrocytic-specific genes were highly expressed in all IP samples, whereas the large majority of genes specific for neurons, MOs, OPCs, microglia, endothelial cells or pericytes were enriched in all UB samples (Fig. 3b). Thus, we can confirm that TRAP consistently enriched for astrocytic-specific genes in all experimental groups. In addition, in the IP samples we measured the expression of several genes involved in pathways known to be important for astrocytic functions [13, 19, 25, 26]. Expression levels of several of these genes were affected by behavioral state (Fig. 3c). This was the case for *Gjb6*, *Ppp1r3c*, and *Adcy10*, which were upregulated in both W and SD relative to S. Other genes, such as *Slc1a3* and *P2ry2*, increased their expression only in SD, while *P2ry4* showed higher expression in S than SD. To test whether changes observed at the transcription level were followed by changes at the protein level, we considered the list of all astrocytic-specific genes that were differentially expressed across behavioral states, and whose corresponding protein was reported to be exclusively expressed in astrocytes. From this list, we selected *Gjb6* and *Slc1a3*, which code, respectively, for the gap-junction subunit connexin30 (Cx-30) and for the glutamate transporter GLAST, two well-characterized astrocytic proteins. Both genes showed increased mRNA levels in SD relative to S (>30 %, *P* < 0.01). Western blotting experiments using cerebral cortex homogenates of S (*n* = 4) and SD (*n* = 4) mice found that the expression of both Cx-30 and GLAST was also increased in SD relative to S (*P* = 0.02 for both proteins, Fig. 3c, right panel).

#### Astrocytic genes are affected by sleep and wake independent of time of day

We compared astrocytic IP samples (18 in total) from S, W and SD mice (*n* = 6 per group) to identify the specific effects of sleep and wake on astrocytic gene expression. To be included in the analysis, probesets needed to be called “present” in at least one sample, which was the case for ~74 % of all probesets (33,255 out of 45,101). All these 33,255 probesets were analyzed, whether or not they were enriched in IP samples relative to UB samples. This is because our primary goal was to identify sleep/wake-dependent genes expressed in astrocytes, independent of whether they were overrepresented in this cell type relative to neurons or oligodendrocytes.

First, we compared IP samples collected at 3 am (W mice) with IP samples collected at 3 pm (S + SD) and found that 152 probesets, representing 139 unique genes, were differentially expressed (*P* < 0.01, W vs S + SD). Thus, the expression levels of ~0.5 % of astrocytic genes changed due to time of day, independently of behavioral state (Additional file 1: Table S1 and Additional file 2: Table S2). Next, we compared IP samples collected after sleep with IP samples collected after spontaneous wake and sleep deprivation, and found that 498 probesets, representing 451 unique genes (~1.4 %), changed their expression because of behavioral state independently of time of day (*P* < 0.01, S vs W + SD, Additional file 3: Table S3 and Additional file 4: Table S4). As in previous work [27], we considered “sleep” genes to be only those that were upregulated in the sleep group (6–7 hr of sleep) relative to both the spontaneous wake group (6–7 hr at night), and the forced wake group (4 hr of sleep deprivation after spending most of the night awake), whereas “wake” genes were those with higher expression in both spontaneous wake and sleep deprivation groups than in the sleep group. Since spontaneous wake occurred at night and sleep deprivation during the day, sleep and wake genes as defined here reflect changes due to behavioral state rather than circadian factors or exposure to light.

**Fig. 4:**
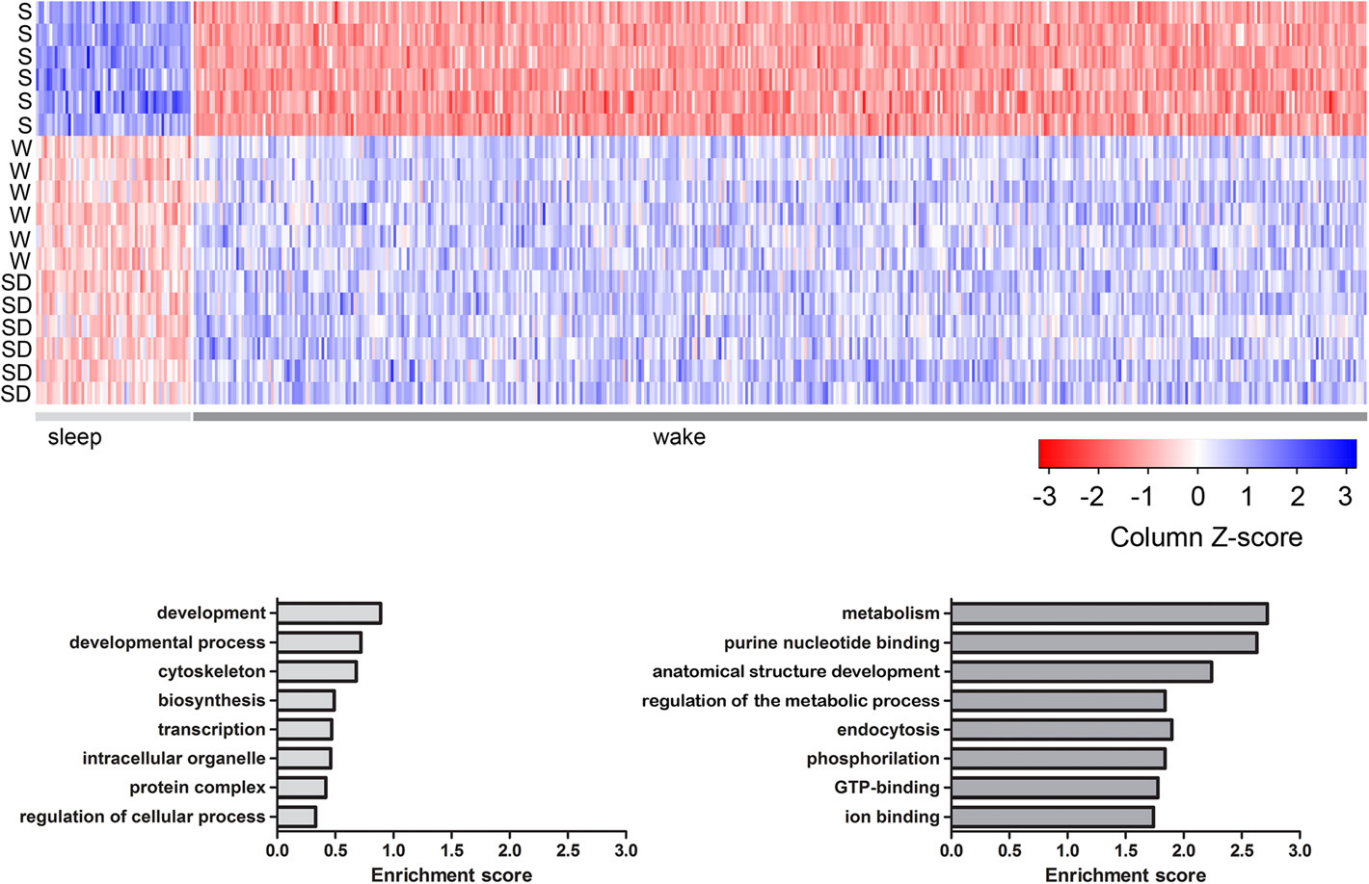
Functional characterization of genes differentially expressed in sleep (S) and wake (W + SD). Top: Heat diagrams show the probeset intensity for each individual animal in the three experimental conditions. Bottom: Functional annotation analysis (DAVID default settings, except for kappa = 4, similarly threshold = 0.7) for S (*n* = 55) and W + SD (*n* = 396) genes. The top eight functional annotation clusters in order of enrichment score are shown for S (*left*) and W + SD (*right*)

The vast majority of genes (396, representing 1.3 % of all analyzed probesets) that changed their expression because of behavioral state were wake genes, while only 55 genes (0.1 % of all analyzed probesets) were sleep genes. A statistical approach using gene annotation enrichment analysis (DAVID) was carried out, together with an extensive analysis of the literature, to elucidate the biological processes, molecular functions and cellular components associated with sleep and wake in astrocytes (Fig. 4, Additional file 5: Table S5 and Additional file 6: Table S6). Despite the short list of sleep genes, we found enrichment of genes involved in cell development and proliferation (*Pax3*, *Pax7*, *Rab38*, *Tsnaxip1*, *Spata4*, *Sphk2* and *Pik3ca*) and biosynthesis (*Fbp1* and *Pigs*). Other sleep genes included *Slc16a1*, which codes for the MCT1 transporter involved in the astrocyte–neuron lactate shuttle [28, 29] and whose activity in the hippocampus is essential for long-term memory formation [30]. By contrast, the functional categories enriched during wake included cell metabolism (*Adcy10*, *Foxf2*, *Cox8a*, *Cox19* and *Ppp1r3c*), nucleotide binding (*Gem*, *Pak3*, *Rhoh*, *Trio*, *Kif15* and *Ttl*), anatomical structure development (*Klf4*, *Cdh6*, *Ptp4a1*, *Uhrf1*, *Trp53*, *Eif2b5* and *Hoxc6*), endocytosis (*Mrc2*, *Ehd2*, *Hip1* and *Mertk*), and ion binding (*Kcnmb2*, *Kcnh5*, *Kcnj1* and *Cacna1a*). Overall, a large category of astrocytic genes modulated by behavioral state was related to the extracellular matrix and/or the cytoskeleton, including sleep genes *Arap3* (involved in actin cytoskeleton remodeling), collagen type IV alpha 4 (*Col4a4*), *Ceacam1*, the actinin-associated LIM protein *Pdlim3*, and actin gamma (*Actg1*). Wake genes included *Trio*, *Gem* and *Synj2*, all of which have been involved in cytoskeleton modifications and the elongation of astrocytic processes, as well as genes coding for integrins *(Itgal*, *Itgb6* and *Itgb1)* and other extracellular matrix proteins *(Efemp2*, *Emilin3* and *Emid2)*, the mannose receptor C2 that binds and internalizes collagen *(Mrc2)*, the proteoglycan syndecan 4 *(Sdc4)*, the endoglycosidase heparanase *(Hpse)*, and others *(Mitd1*, *Ptp4a1*, *Ehd2*, *Nrap* and *Smagp)*.

As discussed before, we applied a conservative criterion to define wake genes, considering only those upregulated in both spontaneous wake and forced wake. When the two wake conditions were compared to each other, we found 584 differentially expressed unique genes, including 200 upregulated in W and 384 upregulated in SD (Additional file 7: Table S7). The number of differentially expressed genes was two- to threefold higher when S was compared to either W or SD: 1014 genes in S vs W (204 upregulated in S, 810 in W) and 1439 in S vs SD (208 upregulated in S, 1231 in SD). This suggests that W and SD are more similar to each other than either wake condition to sleep. The biological processes most enriched in W and SD were cell adhesion and metabolism, respectively (Additional file 8: Figure S1), but the interpretation of these differences is not straightforward, because W and SD differ by many factors, including “quality” of wake (spontaneous vs forced), duration of wake (SD > W by several hours), presence or absence of light, and time of day (3 am vs 3 pm). Of note, most genes differentially expressed between W and SD also differed between S and SD (Additional file 7: Table S7), indicating that their expression reflects the duration of wake and/or the specific presence of forced wake. Only a minority (~30 %) of genes instead differed between W and SD but not in other comparisons (S vs SD, S vs W-SD, 3 am vs 3 pm), including 77 genes known to be upregulated in SD and 41 upregulated in W. These could be “light” genes, responding to the presence or absence of light, respectively (Additional file 7: Table S7).

### Ultrastructural studies

#### Astrocytic coverage of cortical dendritic spines increases after extended wake

**Fig. 5:**
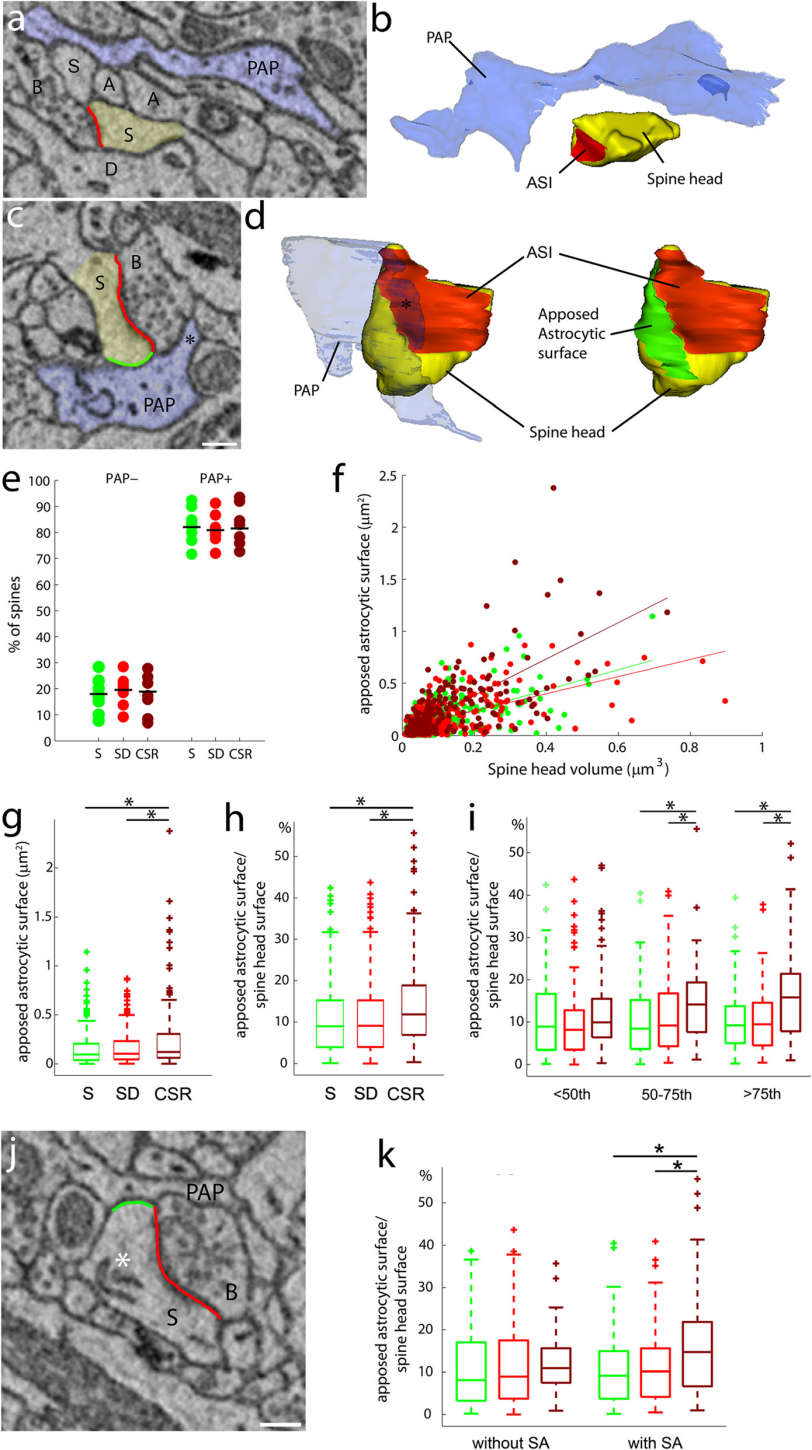
Astrocytic coverage of cortical synapses increases after CSR. **a**–**d** Examples of electron microscope images showing PAP^−^ (**a**) and PAP^+^ (**c**) spines, with their relative 3D reconstructions (**b**, **d**). In (**a**) and (**c**), PAPs are depicted in *light blue* and spine heads (S) are in *yellow. B* = presynaptic bouton; *A* = axon; *D* = dendrite. The ASI is traced in *red* and the apposed astrocytic surface on the spine head is in *green. Scale bar* = 250 nm. In (**d**), the PAP embracing the spine (*) has been made transparent to allow a view of the spine head underneath. Note that the apposed astrocytic surface (*green*) contacts the ASI (*red*) on one side. **e** Percentage of PAP^−^ and PAP^+^ spines per dendrite in layer II cortical fields for S (*n* = 9 dendrites; PAP^−^, 17.95 ± 6.54 %; PAP^+^, 82.05 ± 6.53 %), SD (*n* = 9 dendrites; PAP^−^, 19.16 ± 5.18 %; PAP^+^, 80.84 ± 5.18 %) and CSR (*n* = 8 dendrites; PAP^−^, 18.37 ± 7.07 %, PAP^+^, 81.62 ± 7.07 %). PAP^−^, Kruskal–Wallis, *P* = 0.96; PAP^+^, Kruskal–Wallis, *P* = 0.96. **f** Correlation between area of the apposed astrocytic surface and spine head volume in S (*n* = 250, *green*, *r* = 0.61, *P* < 0.0001), SD (*n* = 300, *red*, *r* = 0.6, *P* < 0.0001) and CSR (*n* = 254, *brown*, *r* = 0.69, *P* < 0.0001). **g** Apposed astrocytic surface area in S (*n* = 250, 0.15 ± 0.17 μm^2^), SD (*n* = 300, 0.16 ± 0.17 μm^2^) and CSR (*n* = 254, 0.23 ± 0.29 μm^2^). SD vs CSR, Mann-Whitney (MW), *P < 0.0001; S vs CSR, MW, *P < 0.0001. **h** Apposed astrocytic surface area to spine head surface area ratio in S (*n* = 250, 10.77 ± 8.59 %), SD (*n* = 300, 10.65 ± 8.38 %) and CSR (*n* = 254, 13.89 ± 9.84 %). SD vs CSR, MW, *P = 0.003, S vs CSR, MW, *P = 0.0005. **i** Apposed astrocytic surface area to spine head surface area ratio for spines with small (head volume between 0th and 50th percentiles), medium (50th to 75th percentiles) and large (>75th percentile) spine head volume in S (*green*), SD (*red*) and CSR (*brown*). Note that CSR showed higher ratios than S and SD for medium (MW; SD vs CSR, *P = 0.03; S vs CSR, *P = 0.027) and large spines (MW; SD vs CSR, *P = 0.0003; S vs CSR, *P = 0.0002). **j** Example of spine head including the spine apparatus (*). *PAP* indicates the astrocytic process, *S* the spine head and *B* the presynaptic bouton. *Scale bar* = 300 nm. **k** Apposed astrocytic surface area to spine head surface area ratio for spines with and without the spine apparatus in S (*green*), SD (*red*) and CSR (*brown*). Note that CSR showed higher ratios than S and SD for spines with spine apparatus (MW; SD vs CSR, *P = 0.03; S vs CSR, *P = 0.0003). All values are mean ± standard deviation

Astrocytes are plastic cells. They are capable of expanding their fine peripheral astrocytic processes (PAPs) in close proximity to synapses in response to increased synaptic activity [4, 31, 32]. In vitro and in vivo experiments have shown that these plastic events can take place in a few hours and have functional consequences for synapses [4, 33]. Our microarray results suggested that astrocytic pathways involved in cytoskeleton modification and elongation of PAPs were modulated by sleep and wake. To test this hypothesis directly, we used SBF-SEM to study PAP dynamics in relation to changes in behavioral state. Specifically, we focused on the interaction between PAPs and dendritic spines in layer II of the prefrontal cortex, because supragranular layers are highly plastic [17] and the SWA of NREM sleep, which reflects the need for sleep and its depth, is largest in frontal areas [18]. The great majority (>95 %) of cortical spines are associated with excitatory synapses [34, 35]. We focused on dendritic branches of similar size (diameter 0.5–1.3 μm, length 9–29.6 μm) and within each segmented portion of the dendrite all spines were analyzed (filopodia were not included in the analysis). The dataset included 317 spines from a sleep group (S, 6–8 hr of sleep, three mice, ~100 spines per mouse), 368 spines from an acute sleep deprivation group (SD, 6–8 hr of sleep deprivation, three mice, ~150 spines per mouse), and 310 spines from a chronic sleep restriction group (CSR, 4 days of chronic sleep restriction with ~70 % of sleep loss, three mice, ~100 spines per mouse). For each spine, the spine head, the axon–spine interface (ASI), i.e. the interfacing surface between the axonal bouton and the spine head, and the PAPs were manually segmented and reconstructed in 3D (Fig. 5a–d; Additional file 9: Table S8).

First, we classified spines depending on whether or not they were reached by PAPs. PAP positive (PAP^+^) spines were defined as those contacted by PAPs in any location of the spine head, independent of the presence of PAPs contacting the spine neck and/or the axonal bouton. Across all animals, ~81 % of spines were PAP^+^ and ~19 % were PAP negative (PAP^−^) (Fig. 5e), consistent with findings in layer IV of the mouse barrel cortex, where only ~10 % of spines lack astrocytic contact [31]. Compared to PAP^+^ spines, PAP^−^ spines had smaller mean head volume (0.053 ± 0.05 μm^3^ vs 0.11 ± 0.12 μm^3^, Kolmogorov–Smirnov, *P* < 0.0001) and smaller mean ASI (0.11 ± 0.13 μm^2^ vs 0.24 ± 0.27 μm^2^, Kolmogorov–Smirnov, *P* < 0.0001; data not shown). Between-group analysis showed that the number of PAP^+^ and PAP^–^ spines did not differ across groups (PAP^+^, *P* = 0.96; PAP^−^, *P* = 0.96; Fig. 5e).

Next, for PAP^+^ spines, we calculated the astrocytic coverage, i.e. the extent of the astrocytic surface apposed to the spine head. In all three groups, we found a positive correlation between spine head volume and apposed astrocytic surface (Fig. 5f). Thus, not only larger spines are more likely to be contacted by astrocytes, but the extent of this contact also increases with spine size. Crucially, we also found that the apposed astrocytic surface differed across groups (Kruskal–Wallis, *P* = 0.0009), being higher in CSR relative to both S (*P* < 0.0001) and SD (*P* < 0.0001; Fig. 5g). We also calculated the ratio between apposed astrocytic surface and the whole spine head surface, and found that this parameter differed across the three groups (Kruskal–Wallis, *P* < 0.0001), again with CSR having higher values than both S (*P* = 0.0005) and SD (*P* = 0.003; Fig. 5h).

We further assessed whether the increased astrocytic coverage observed in CSR was limited to spines with a specific size by subdividing spine heads into three subgroups: small (head volume between 0th and 50th percentiles), medium (50th to 75th percentiles) and large spines (>75th percentile). The increase in apposed astrocytic surface in CSR was significant in medium (SD vs CSR, *P* = 0.03; S vs CSR, *P* = 0.027) and large spines (SD vs CSR, *P* = 0.0003; S vs CSR, *P* = 0.0002; Fig. 5i). Finally, previous work has shown that large mature spines usually contain the spine apparatus, an organelle composed of stacked smooth endoplasmic reticulum [36]. We tested whether the increased coverage observed in CSR mice applied specifically to spines with the spine apparatus, and found that this was the case (SD vs CSR, *P* = 0.03; S vs CSR, *P* = 0.0003; Fig. 5j, k).

#### Wake brings astrocytic processes closer to the synaptic cleft

**Fig. 6:**
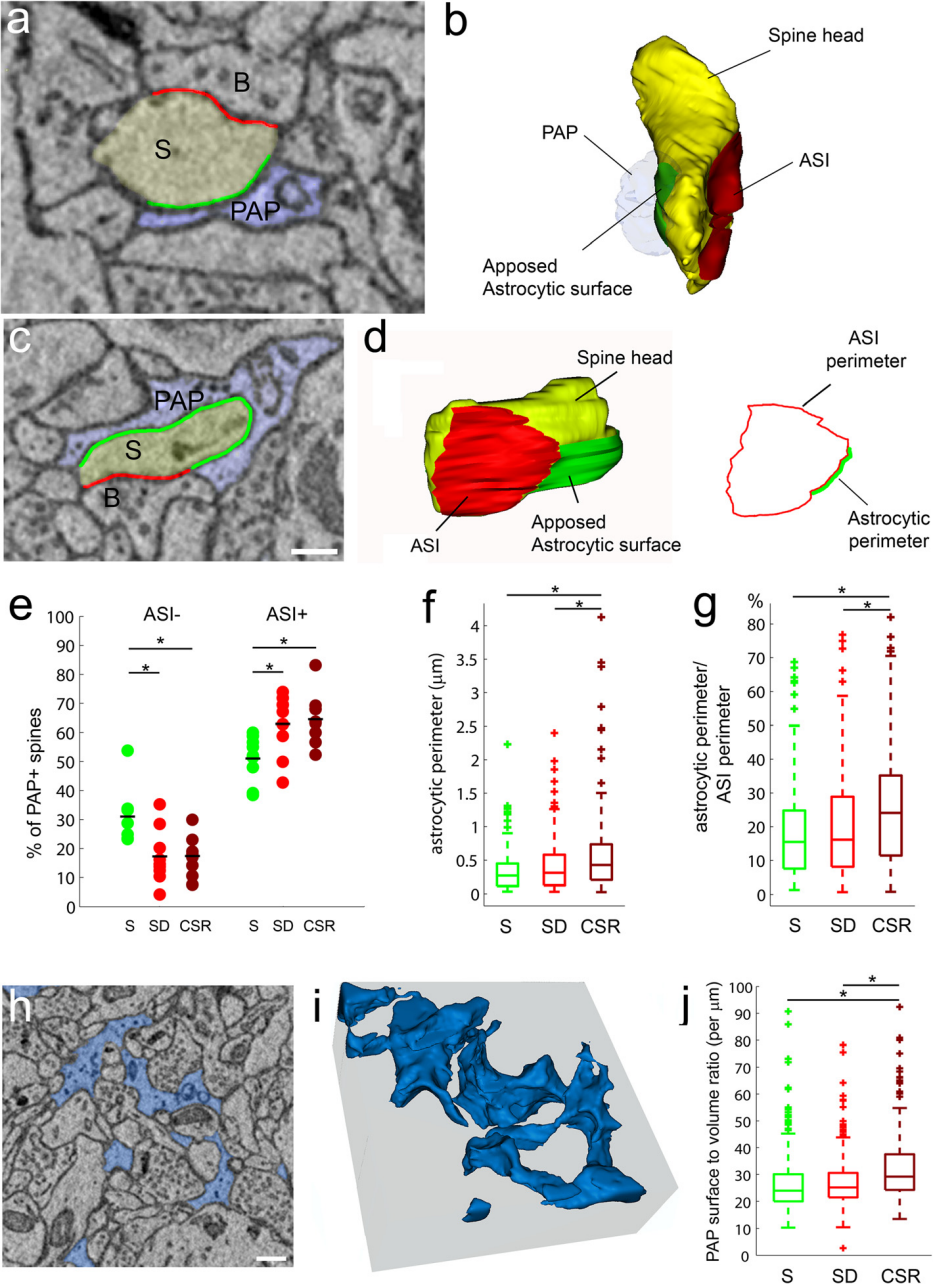
Wake brings astrocytic processes closer to the synaptic cleft. **a**–**d** Examples of electron microscope images showing PAP^+ASI–^ (**a**) and PAP^+ASI+^ (**c**) spines, with their relative tridimensional reconstructions (**b**, **d**). PAPs are depicted in *light blue* and spine heads (S) are in *yellow. B* = presynaptic bouton, *A* = axon, *D* = dendrite. The ASI is traced in *red* and the apposed astrocytic surface on the spine head is in *green. Scale bar* = 250 nm. In (**b**), the PAP embracing the spine (*) has been made transparent to allow a view of the spine head underneath. Note that the apposed astrocytic surface (*green*) does not reach the ASI (*red*). In (**d**), the orientation of the structure shows the contact between the apposed astrocytic surface (*green*) and the ASI. The line drawing on the right shows the ASI perimeter (*red*) and the astrocytic perimeter (*red*) in 3D. **e** Percentage of PAP^+ASI–^ and PAP^+ASI+^ spines per dendrite in layer II cortical fields for S (*n* = 9 dendrites; PAP^+ASI–^, 31.1 ± 9.6 %; PAP^+ASI+^, 51.09 ± 7.88 %), SD (*n* = 9 dendrites; PAP^+ASI–^, 17.38 ± 9.45 %; PAP^+ASI+^, 62.93 ± 10.55 %) and CSR (*n* = 8 dendrites; PAP^+ASI–^, 17.51 ± 7 %; PAP^+ASI+^, 64.53 ± 9.34 %). PAP^+ASI–^, S vs SD, MW, * *P* = 0.01; S vs CSR, MW, *P = 0.001; PAP^+ASI+^, S vs SD, MW, * *P* = 0.02; S vs CSR, MW, *P = 0.0033. **f** Astrocytic perimeter in S (*n* = 148, 0.35 ± 0.32 μm), SD (*n* = 207, 0.42 ± 0.4 μm) and CSR (*n* = 171, 0.59 ± 0.63 μm). SD vs CSR, MW, *P = 0.002; S vs CSR, MW, *P < 0.0001. **g** Astrocytic perimeter to ASI perimeter ratio in S (*n* = 148, 18.66 ± 14.83 %), SD (*n* = 207, 19.77 ± 15.79 %) and CSR (*n* = 171, 25.25 ± 17.08 %). SD vs CSR, MW, *P = 0.0001; S vs CSR, MW, *P = 0.0005. **h**, **i** Example of portion of neuropil occupied by PAP (depicted in *light blue*) and its relative 3D reconstruction. *Scale bar* = 300 nm. **j** PAP surface-to-volume ratio in S (*n* = 314, 27 ± 13 per μm, S vs CSR, MW, *P < 0.0001), SD (*n* = 365, 27 ± 12 per μm, SD vs CSR, MW, *P < 0.0001) and CSR (*n* = 307, 33 ± 13 per μm). All values are mean ± standard deviation

Astrocytes can contact spine heads in two different ways, depending on whether or not the contact includes the area around the synaptic cleft (ASI, Fig. 6a–d). Thus, we subdivided PAP^+^ spines in two subgroups, PAP^+ASI–^ and PAP^+ASI+^, which accounted for ~25 % and ~75 % of all PAP^+^ spines, respectively, and tested whether their number was affected by sleep and wake. We found that this was the case (PAP^+ASI–^, Kruskal–Wallis, *P* = 0.0058; PAP^+ASI+^, Kruskal–Wallis, *P* = 0.012). Specifically, the proportion of PAP^+ASI+^ spines was higher in SD (*P* = 0.02) and CSR (*P* = 0.0033) than in S, while the opposite was true for PAP^+ASI–^ (Fig. 6e). Of note, in all groups large spines were approached by PAPs at the ASI level more frequently than small spines (Kolmogorov–Smirnov, *P* < 0.0001; data not shown). To rule out that the observed PAP changes were unique to conditions of forced wake, such as SD and CSR, we also studied mice sacrificed after at least 6 hr of spontaneous wake (W, three mice, 279 spines). The proportion of PAP^+ASI+^ spines in W was significantly higher than S (68.23 ± 6.26 % vs 51.09 ± 7.88 %, *P* = 0.023; Additional file 10: Figure S2), and comparable to SD (MW, *P* = 0.59) and CSR (MW, *P* = 0.79), indicating that the movement of PAPs toward the synaptic cleft is not caused by the stress of sleep deprivation.

Next, we measured whether the extent of the astrocytic coverage at the level of the synaptic cleft also increased with wake. We calculated the portion of ASI perimeter that was contacted by PAP (astrocytic perimeter) and the ratio between the astrocytic perimeter and the whole ASI perimeter (Fig. 6d). Both parameters increased in CSR relative to both S (astrocytic perimeter, *P* < 0.0001; ratio, *P* = 0.0005) and SD (astrocytic perimeter, *P* = 0.002; ratio, *P* = 0.0001; Fig. 6f, g). Furthermore, since in all groups we found a positive correlation between astrocytic perimeter and ASI (S, *r* = 0.41, *P* < 0.0001; SD, *r* = 0.36, *P* < 0.0001; CSR, *r* = 0.51, *P* < 0.0001), and the size of the ASI is positively correlated with the spine head volume (*P* < 0.0001 in all groups), we subdivided the spines depending on head size or presence of a spine apparatus, as done before. The ratio between astrocytic perimeter and ASI perimeter increased significantly in CSR compared to S in spines of the largest size (MW, *P* = 0.0027), as well as in spines with a spine apparatus (MW, *P* = 0.003), although CSR showed a trend to be higher relative to both S and SD also in spines without spine apparatus (not shown). The ratio between astrocytic perimeter and ASI perimeter also increased in CSR relative to SD in small spines (MW, *P* = 0.002). Overall, the CSR changes at the synaptic interface are consistent with those seen at the level of the entire spine head, with the most significant increases in astrocytic coverage occurring in large spines with the spine apparatus.

Finally, we tested whether the increased extension of the astrocytic coverage in CSR may result from an overall enlargement of the astrocytic profiles in the neuropil. We measured the PAP volume in a portion of neuropil surrounding every spine considered in the previous analysis (Fig. 6h, i). The volume occupied by PAPs did not change across experimental groups, although an increasing trend in CSR was noticeable (S, 8.31 ± 6.55 %; SD, 8.61 ± 8.17 %; CSR, 9.19 ± 6.8 %; S vs CSR, MW, *P* = 0.1; S vs SD, MW, *P* = 0.97; SD vs CSR, MW, *P* = 0.07; not shown). However, the PAP surface-to-volume ratio was significantly higher in CSR than S (*P* < 0.0001) and SD (*P* < 0.0001; Fig. 6j). Since the PAP volume is slightly increased in CSR relative to S and SD, an increase of the PAP surface-to-volume ratio can be explained only by an increase of the astrocytic surface. Thus, extended periods of wake have little effect on PAP volume, but increase the PAP surface in the neuropil.

## Discussion

We recently found that 2.1 % of the genes expressed in oligodendrocytes are modulated by behavioral state, with similar numbers of wake and sleep genes being modulated (404 vs 310, respectively) [37]. The number of state-dependent genes is smaller in astrocytes (1.4 %), and more than sevenfold higher in wake than in sleep (396 vs 55). The 18 mice used in the current study were processed in two independent experiments, and in both cases, the results showed a strong bias towards wake genes. Thus, the imbalance in the number of sleep and wake genes is likely not dependent on technical issues, and may reflect the fact that wake is a highly active state for astrocytes, as suggested also by the enrichment of wake genes related to energy metabolism. A case in point is *Adcy10*, the gene coding for soluble adenylyl cyclase, an enzyme that stimulates glycogen breakdown and the release of lactate in response to neuronal depolarization [38]. The expression of the gene for protein targeting to glycogen (*PTG* and *Ppp1r3c*) also increased with wake, consistent with previous studies that used cell sorting followed by qPCR to measure a few astrocytic genes in a subset of cortical astrocytes (GFAP^+^) [39, 40]. These studies also found a small wake-related upregulation of *Slc2a1*, which codes for the glucose transporter GLUT-1, and *Slc1a2*, which encodes the major glutamate transporter (GLT-1). These genes were not upregulated during wake in our study, most likely because microarrays are not as sensitive as qPCR, although we observed a significant increase of *Slc1a3*, which codes for the second glial glutamate transporter (GLAST), in sleep deprivation relative to sleep.

Among the sleep genes, we found significant enrichment for a few functional categories including development and cell proliferation. The same categories are enriched during sleep in oligodendrocytes, and the rate of proliferation of oligodendrocyte precursors doubles in sleep relative to wake [37]. *Cirp*, coding for cold inducible RNA binding protein, is also upregulated by sleep in astrocytes and oligodendrocytes [37], and was identified as a sleep gene in previous studies that pooled transcripts from all brain cells [41–43]. CIRP is required for high-amplitude circadian gene expression in fibroblasts, but daily oscillations in Cirp mRNA levels are driven by changes in temperature, not by the circadian clock [44]. CIRP binds to the untranslated regions of hundreds of transcripts, including many that promote protein synthesis and cell proliferation and inhibit apoptosis [44]. Thus, *Cirp* induction may be one of the mechanisms by which sleep favors cell proliferation and biosynthetic processes. Another sleep gene codes for the ubiquitin-like modifier-activating enzyme 1 (UBA1). In human cells, UBA1 mediates the repair of DNA double-strand breaks (DSBs) caused by metabolic stress or other insults [45]. We recently found that the number of DSBs increases in cortical neurons after a few hours of exploration, confirming previous evidence [46]. We also found that once induced, DSBs decline when exploratory behavior is followed by sleep but not when it is followed by forced wake (Bellesi et al., unpublished results). However, we currently do not know whether wake-related DSBs also occur in astrocytes, and whether their repair depends on astrocytic *Uba1* induction.

Many genes coding for components of the extracellular matrix and cytoskeleton, including integrins, collagen and syndecan, were modulated by sleep or wake. These genes included *Trio*, *Synj2* and *Gem*, whose upregulation during wake suggested that astrocytic pathways involved in PAP elongation are promoted by wake. *Trio* codes for a guanine nucleotide-exchange factor involved in the activation of the Rac pathway [47], and activation of Rac1, a ras-related GTP-binding protein, is essential for promoting PAP elongation via actin reorganization [33]. *Synj2* codes for synaptojanin 2, a phosphoinositide phosphatase that mediates Rac1-regulated functions and promotes the formation of astrocytic lamellipodia [48]. Finally, *Gem* codes for a GTP-binding protein that interacts with Ezrin, a cytoskeleton linker that has an actin-binding site and contributes to the formation of specialized structures of the plasma membranes [49]. Ezrin is specifically expressed in astrocytes and it is essential for the structural plasticity of PAPs [50].

Using SBF-SEM, we found that independent of behavioral state, a stable number of spines in layer II of the mouse prefrontal cortex lack astrocytic coverage. These “bald” spines represent a minority of all cortical spines (<20 %) and are of small size, and thus may not require perisynaptic astrocytes because they are presumably newly formed and/or less active. On the other hand, the head of most spines is contacted by astrocytes. These spines are of medium or large size and thus presumably more active and in need of active mechanisms for the clearance of glutamate and K^+^ ions. Indeed, previous work showed that PAPs preferentially target large synapses [32, 51, 52] and astrocytic coverage of the spine head correlates with spine size [32]. Even short periods of wake are sufficient to bring astrocytic processes closer to the synaptic cleft, possibly because wake active synapses are in greater need of the housekeeping functions mediated by astrocytes. In line with this hypothesis, overall firing rates in the cortex are higher in wake than in sleep [53], and extracellular levels of glutamate increase during wake and decline during sleep [11, 54]. On the other hand, PAP retraction from the synaptic cleft during sleep might favor glutamate spillover and cross-talk with neighboring synapses [55, 56], thereby contributing to the synchronization of neuronal activity typical of NREM sleep.

CSR not only moves astrocytic processes towards the synaptic cleft but also increases overall astrocytic coverage, including the ASI. In the visual cortex, astrocytic coverage in direct apposition to pre- and postsynaptic elements increases with chronic exposure to a complex environment [57]. In the barrel cortex, whisker stimulation for 24 hr leads to an increase in the density of excitatory and inhibitory synapses on spines, in the number of cortical spines contacted by astrocytes and in the astrocytic coverage at the ASI [31]. An increase in perisynaptic coverage also occurs in the hippocampus after induction of long-term potentiation [32, 58] and after kindling [59]. Of note, in vitro and in vivo experiments suggest that sensory stimulation and LTP-inducing stimuli (LTP) may first displace astrocytic processes away from spines, and then lead to an increase in astrocytic coverage and spine stability 24 hr later [60, 61]. Ongoing experiments are testing whether the expansion of perisynaptic astrocytes after sustained wake results from sustained neuronal activity, and/or is secondary to an overall increase in the number and/or size of synapses. Interestingly, acrobatic training increases both the number of synapses and glial volume in the cerebellum, while forced exercise induces angiogenesis but not additional glia [62]. This suggests that glial hypertrophy, presumably mainly of astrocytes, may reflect learning-related synaptogenesis and not simply increases in synaptic activity [62].

In addition to increased synaptic coverage, CSR is associated with an augmented astrocytic surface in the neuropil. Given their lamellar morphology, astrocytic processes account for most of the cell surface area, even if they contain a small portion of the cytoplasmatic volume. This remarkably large surface-to-volume ratio [63, 64] provides astrocytes with ample space for positioning a large number of ion channels, ligand receptors and uptake transporters that are necessary to sustain glia–neuron interactions [65]. Therefore, the increase in astrocytic surface after CSR may represent the astrocytic reaction to the sustained neuronal activity characterizing extended periods of wake.

It was recently found that the extracellular space enlarges significantly, by ~60 %, in conditions of synchronized cortical activity such as slow-wave sleep and anesthesia [66]. This effect was to a large extent dependent on the locus coeruleus [66] and could be mediated, at least in part, by astrocytes [67]. Our findings are compatible with this hypothesis, because we found that astrocytic processes are closer to synapses in wake than in sleep. However, it is difficult to relate the two studies directly, because the extracellular space is significantly reduced in electron microscopy samples that underwent chemical fixation and dehydration, such as ours. On the other hand, our finding that behavioral states affect the PAP surface-to-volume ratio suggests that during sleep, astrocytic processes may assume a more rounded and less complicated shape, which may promote the exchange of molecules in the extracellular space and their washout through the glymphatic pathway [66, 68].

The mechanisms underlying the sleep/wake effects on astrocytic processes are likely to be complex, because the activity of cortical glutamatergic neurons, as well as that of arousal systems, including the noradrenergic, cholinergic, histaminergic and orexinergic systems, are higher in wake than in sleep [69]. Astrocytes from different brain regions may respond to different combinations of neurotransmitters [2]. In vivo calcium imaging experiments show that locomotion, sensory stimulation and the startle response trigger widespread coordinated increases in astrocytic activity in the cerebral cortex and cerebellum, and that the locus coeruleus, but not the cholinergic system, plays a major role in these responses via activation of α_1_ adrenoceptors [70–72]. The locus coeruleus may therefore provide a major activating signal to astrocytes during wake in the cortex, while the cholinergic system may have a prominent role in the hippocampus [73].

## Conclusions

We show that sleep upregulates select genes, like *Cirp* and *Uba1*, whereas wake upregulates many genes related to metabolism, the extracellular matrix and cytoskeleton. Using SBF-SEM, we find that short wake brings astrocytic processes closer to the synaptic cleft, while CSR also extends the overall astrocytic coverage of the synapse and increases the available astrocytic surface in the neuropil. Wake-related changes likely reflect an increased need for glutamate clearance, and are consistent with an overall increase in synaptic strength when sleep is prevented. The reduced astrocytic coverage during sleep, instead, may favor glutamate spillover, thus promoting neuronal synchronization during NREM sleep.

## Methods

### Molecular studies

#### Animals

Eight-week-old heterozygous ALDH1L1 – eGFP-L10a BAC transgenic mice and wild-type littermates (of either sex, properly balanced) were used in this study. Heterozygous offspring were obtained from homozygous ALDH1L1 - eGFP BAC transgenic mice bred with C57BL/6 wild-type mice. All animal procedures followed the National Institutes of Health Guide for the Care and Use of Laboratory Animals and facilities were reviewed and approved by the institutional animal care and use committee (IACUC) of the University of Wisconsin-Madison, and were inspected and accredited by association for assessment and accreditation of laboratory animal care (AAALAC).

#### Electroencephalographic recordings

Under isoflurane anesthesia (1–1.5 % volume), mice (*n* = 5) were implanted bilaterally for chronic EEG recordings with epidural screw electrodes over the frontal (millimeters from the bregma: anteroposterior, AP +1, mediolateral, ML +1) and parietal cortex (AP −2, ML +2) and cerebellum (reference electrode and ground). Electrodes were fixed to the skull with dental cement. Two stainless steel wires (diameter 0.4 mm) were inserted into neck muscles to record an electromyogram (EMG). Single implanted mice were housed in transparent Plexiglas cages (36.5 × 25 × 46 cm) with continuous access to a running wheel, and kept in soundproof recording boxes for the duration of the experiment (light/dark 12:12, light on at 8 am, 23 ± 1 °C; food and water available ad libitum and replaced daily at 8 am). Recordings started only after the sleep/wake cycle had completely normalized, usually about 1 week post-surgery. Mice were connected by a flexible cable to a commutator (Airflyte) and recorded continuously for 2 weeks using a Grass mod. 8 polygraph (Grass Instruments). EEG and EMG signals were amplified and filtered as follows. The EEG measurements used a high-pass filter at 0.1 Hz and a low-pass filter at 35 Hz. The EMG measurements used a high-pass filter at 10 Hz and a low-pass filter at 100 Hz. All signals were digitalized at 128 Hz and stored on a computer. EEG power spectra were computed by a fast Fourier transform routine for 4-s epochs (0.25 Hz resolution). Wake, NREM sleep and REM sleep were manually scored off-line (SleepSign, Kissei Comtec) in 4-s epochs according to standard criteria. Epochs containing artifacts, predominantly during active wake, were not used for spectral analysis. The vigilance state could always be determined.

#### Video recordings of behavioral states

To avoid possible tissue lesion and inflammation due to the implant of EEG electrodes, the behavioral states in mice used for TRAP/array were determined by continuous monitoring with infrared cameras (OptiView Technologies). We previously found that video monitoring cannot distinguish NREM sleep from REM sleep, but it consistently estimates total sleep time with ≥90 % accuracy [74]. Motor activity was quantified by custom-made video-based motion detection algorithms (MATLAB), as previously described [37]. Sleep/wake criteria for the experimental groups were as follows: awake mice were killed during the dark phase (~3–5 am) at the end of a long period of wake (>1 hr, interrupted by periods of sleep of <5 min), and after spending at least 70 % of the previous 6–7 hr awake. Sleeping mice were killed during the light period (~3–5 pm), at the end of a long period of sleep (>45 min, interrupted by periods of wake of <4 min), and after spending at least 75 % of the previous 6–7 hr asleep. Sleep deprived mice were spontaneously awake during most of the dark phase and then kept awake during the first 4 hr of the light period by exposure to novel objects and by tapping on the cage whenever the animals appeared drowsy. Mice were never disturbed when they were spontaneously awake, feeding or drinking.

#### Immunocytochemistry

Mice were deeply anesthetized with isoflurane (3 % volume) and perfused transcardially with a flush (~30 s) of saline followed by 4 % paraformaldehyde in phosphate buffer (PB). Brains were removed, post-fixed in the same fixative overnight and cut on a Vibratome in 50 μm coronal sections, which were collected in PB in serial groups of five and then used for immunocytochemistry. Sections were rinsed in a blocking solution (normal goat serum [NGS] 1.5 % and 0.3 % TritonX for CNP, NGS 5 % and 0.1 % TritonX for Neun, and GFAP) for 1 hr, and then incubated overnight (4 °C) in the same blocking solution containing anti-GFP (1:500, Invitrogen, A21311) and one of the following primary antibodies (anti-CNP, 1:200, Millipore, MAB326; anti-NeuN, 1:200, Millipore, MAB377; anti-GFAP, 1:500, Sigma, G3893). Sections were then exposed for 1.5 hr to NGS (1.5 %) solution containing a mixture of Alexa Fluor 568 (1:250, Invitrogen) and Alexa Fluor 488 (1:250, Invitrogen) conjugated secondary antibodies. Sections were washed, mounted, air-dried and coverslipped using Vectashield mounting medium (H-1000; Vector), and examined with a confocal microscope (Prairie Technologies). Control experiments with single-labelled sections and sections incubated with two primary antibodies and one secondary antibody or with one primary and two secondary antibodies revealed neither bleed-through nor cross-reactivity. Microscopic fields were randomly selected as 512 × 512 pixel images (pixel size 581 nm) and observed with an UPlan FL N × 40 objective (numerical aperture 1.3). To improve the signal/noise ratio, four frames of each images were averaged. Fields were taken from the frontal neocortex (*n* = 3 mice, three sections per mouse). Image processing was performed on ImageJ and Photoshop CS2 (Adobe Systems).

#### Translating ribosome affinity purification and RNA extraction

Under anesthesia S, SD and W mice (*n* = 6 per group) were decapitated and the forebrain regions (cortex and striatum) were quickly dissected. The protocol has been previously described [37]. Briefly, homogenized tissue was centrifuged to obtain a post-mitochondrial fraction, and GFP^+^ polyribosomes were separated by immunoprecipitation using GFP antibody coated beads. Then, mRNA was extracted from both the precipitated portion (IP) and the supernatant (UB).

#### Quantitative PCR

cDNA was synthesized from 7.5 ng of RNA from six IP and six UB samples isolated from sleeping (*n* = 2), awake (*n* = 2) and SD (*n* = 2) animals with SuperScript VILO cDNA synthesis kit (Invitrogen, 11754–050) and diluted fourfold. Real-time PCR was performed using the ABI 7300 Real Time PCR System (in triplicate) following the manufacturer’s standard protocol, with SYBR Green PCR Master Mix (Applied Biosystems, 4309155) in a final volume of 25 μl. Primers included: *Gapdh* forward 5′-CCAGAAGACTGTGGATGGC-3′; *Gapdh* reverse, 5′-TGAGCTTCCCGTTCAGCTC-3′; *Mbp* forward 5′- ATCCAAGTACCTGG CCACAG-3′; *Mbp* reverse 5′-CCTGTCACCGCTAAAGAAGC-3′; *Gfap* forward, 5′-GTAAAGACTGTGGAGATGCGGGATGGTGAGG-3′; *Gfap* reverse 5′-GTGCTGGTGTGGGTGGGAACTGAG-3′; *Syt1* forward 5′-GGCACTCACCATT TTTGGTT-3′ and *Syt1* reverse 5′-AGCTCCAGCAGAACATCTCG-3′. Expression levels were calculated by the ddCT method via Applied Biosystems software, using GAPDH as the normalizing gene and UB samples as the calibrator.

#### Microarray: labeling, hybridization and data analysis

The protocol has been described in Bellesi et al. [37]. Briefly, mRNA from IP (*n* = 6) and UB (*n* = 2) samples was amplified, fragmented and hybridized to Affymetrix GeneChip Mouse Genome 430 2.0 arrays (one chip per sample and sample loading was randomized). Microarray data were then analyzed using the Bioconductor Limma package. To obtain a measure of the enrichment, the expression intensity of each IP mRNA was compared against its UB expression using Welch’s *t*-test with Benjamini–Hochberg false-discovery-rate multiple test correction. Probesets with IP/UB ratio >2 and *P* < 0.01 were considered enriched, while probesets with IP/UB ratio <2 and *P* < 0.01 were considered depleted. Next, IP/UB ratios for the 200 top genes previously found to be enriched in astrocytes [19] were calculated. Finally, to identify transcripts that were differentially expressed across behavioral states (sleep and wake genes, whether or not enriched in astrocytes), IP samples were compared using Welch’s *t*-test with Benjamini–Hochberg false-discovery-rate multiple test correction. All probesets with fold change >30 % and *P* < 0.01 were selected for functional clusterization (DAVID, Database for Annotation, Visualization and Integrated Discovery [75]. The enrichment score was determined through DAVID. It ranks the significance of each annotation cluster based on relatedness of the terms and the genes associated with them. The gene ontology terms were gathered based on the known annotation of the submitted genes with respect to biological process, molecular function and cellular component.

#### Western blotting

Under anesthesia, sleeping and sleep-deprived mice (four S and four SD) were decapitated and the forebrain regions (cortex and striatum) were quickly collected, immediately frozen on dry ice, and stored at –80 °C. Samples were prepared as previously described [37] and stored unprocessed at –80 °C. Protein concentration was determined by the bicinchoninic acid assay (Pierce). Since the expression of housekeeping proteins such as actin and tubulin is affected by sleep and wake [27, 43, 76], a loading control was not used. Instead, equal amounts of protein were pooled together from each animal from the S and SD groups, loaded onto the same gels in three to six replicates (sample loading was randomized), and experiments were repeated four times. In each experiment, equal amounts (10 μg for Cx-30 and 1 μg for GLAST) of homogenate from the S and SD pools were first separated by tris–HCl gel electrophoresis (BioRad) in 1× tris/glycine/sodium dodecyl sulfate buffer (BioRad), transferred to 0.45-μm-pore-size nitrocellulose membranes (BioRad) in 1× tris base/glycine/methanol blotting buffer, and probed with anti-Cx-30 (1:500, Life Technologies, 712200) or anti-GLAST (1:1000, Thermo Scientific, PA5-19709) antibodies. The linear range for each antibody was determined using a five-point dilution series of homogenate prior to running the experimental samples. After exposure to secondary antibodies (1:2000 horseradish peroxidase-conjugated goat anti-rabbit, Millipore; 12–348), bands were visualized using enhanced chemiluminescence (ECL-Prime, Amersham) and captured by the Typhoon™ 9410 Variable Mode Imager (Amersham; see [37] for details). Optical densities were calculated for each band of interest after performing background correction (by subtracting the value of a band immediately above the band of interest in the same lane) and normalized within each gel to the averaged density of S samples.

### Electron microscopy studies

#### Experimental groups

To maximize the possibility of discovering morphological changes induced by sleep and wake, ultrastructural studies were performed on four groups of 4-week-old mice (three mice per group of either sex), since young mice display higher levels of structural plasticity than adult mice. Of note, 4-week-old mice have sleep and wake patterns comparable to those of young adults [77]. Sleeping mice were sacrificed after 6–8 hr of sleep as assessed using motion detection; spontaneously awake mice were sacrificed after at least 6 hr of wake at night; SD mice were sacrificed after 6–8 hr of sleep deprivation enforced by introducing novel objects and by tapping on the cage whenever the animals appeared drowsy. Chronically sleep-restricted mice were subjected to 4 days of CSR using a protocol optimized in our laboratory. In pilot experiments, we demonstrated that sleep across the 4 days is reduced by ~70 % using this approach, which relies on multiple strategies to disrupt sleep, including exposure to novel objects during the day and forced locomotion on a slowly rotating platform during the night. The platform is located above a tray filled with 2–3 cm of water, and the rotation speed is low enough that mice can easily avoid falling into the water as long as they move continuously. Video cameras and/or direct visual observation were used to monitor the mice at all times while on the platform.

#### Scanning Block Face Electron Microscopy

Under deep anesthesia (3 % isoflurane), mice were perfused intracardially with a solution of 0.05 M phosphate buffered saline followed by 2.5 % glutaraldehyde and 4 % paraformaldehyde dissolved in 0.1 M sodium cacodylate buffer (41 °C and pH 7.4). Brains were removed and kept in the same fixative overnight at 4 °C. Tissue slices (120 μm) were cut on a vibratome and kept in a cryoprotectant solution until the day of processing. Sections were rinsed 3 × 10 min each in cacodylate buffer, and incubated for 1 hr on ice with a solution of 1.5 % potassium ferrocyanide/2 % osmium tetroxide. After three rinses in double distilled water, they were exposed to a solution of 1 % thiocarbohydrazide for 20 min at room temperature. Sections were washed with distilled water and placed in 2 % osmium tetroxide for 30 min, washed again, and incubated overnight with 1 % uranyl acetate at 4 °C. The following day, after washing, the tissue was stained with a solution of lead aspartate for 30 min at 60 °C (pH 5.5), washed again and dehydrated using ice-cold solutions of freshly prepared 35, 50, 75, 80, 90, 95 and 100 % ethanol. Sections were placed in propylene oxide for 10 min and then impregnated with 25, 50, or 75 % Durcupan resin mixed with propylene oxide (2 hr each), followed by 100 % Durcupan overnight and then fresh Durcupan for 2 hr. Sections were flat embedded with ACLAR film and placed in a 60 °C oven for 48–72 hr. After polymerization, small squares of resin-embedded tissue (1 mm^2^) from the frontal cortex (AP 1.85 mm; ML 1.5 mm) were excised under a stereomicroscope and glued onto the tip of a metal pin. The block of tissue was trimmed and coated with silver paint to minimize specimen charging during imaging.

#### Image acquisition

Images were obtained using a ΣIGMA™ VP field emission scanning electron microscope (Carl Zeiss NTS Ltd) equipped with 3View® technology (Gatan Inc.), and a backscattered electron detector. Images were acquired using an aperture of 30 μm, high vacuum, acceleration voltage of 1.2 kV, with image resolution (*xy* plane) between 4 and 6 nm. Serial images were obtained by scanning the face of an unsliced block of tissue placed inside the microscope, then cutting off ultrathin slices using an automated microtome within the instrument. The newly exposed surface of a sliced block was rescanned until a stack of images was obtained. The series of images were processed and analyzed using FIJI. TrakEM2, a FIJI plug-in, was used for segmentation of dendrites, spines and astrocytic profiles. Segmentation was performed manually by three operators blind to the experimental condition. Series were randomly assigned to each operator and their work was supervised and checked for consistency by an expert tracer also blind to the experimental condition. Every spine was checked for the presence or absence of PAPs. PAPs were identified by their irregular shapes interdigitating among neuronal profiles, often making contacts with variable portions of the synapse, and by the presence of granules of glycogen distributed over a relatively clear cytoplasm. Other structures contacting the spine head that did not meet the criteria for PAPs were individually inspected through the z-stack and then identified. There are two interfacing surfaces: one between the axonal bouton and the spine head (ASI), and the other is the apposed astrocytic surface on the spine head. These were traced at the contact of two apposed objects in each individual section using the *area lists brush* suitably set at 1 pixel size. In this way, a quasi two-dimensional sheet-like object representing the interfaced region was created along the *z* dimension. The total surface area was calculated by computing the smoothed upper bound surface, according to the formula

$$ \mathrm{Smoothed}\;\mathrm{upper}\;\mathrm{bound}\;\mathrm{surface}={\displaystyle {\sum}_{k=0}^n\left({P}_s(a)\times \frac{1}{2}T\right)+\left({P}_s(b)\times \frac{1}{2}T\right)}+\left[A(a)-A(b)\right] $$

where *n* is the number of sections, *a* and *b* are the traced elements at the top and bottom of a section *k* of thickness *T*, *P*_*s*_ is the smoothed perimeter, and *A* is the area [78]. Finally, the smoothed upper bound surface values were divided by 2 to get an approximate value of the apposed surface. To validate this approach, apposed surface values of 20 apposed elements were also calculated using the *profile tool*, which allows one-dimensional lines to be traced along the contact region between two elements in each individual section. By doing so, the surface area is then computed by summing the length of all the lines and multiplying this value by the thickness of the section [79]. Quantitative analysis showed that the two methods lead to similar results, with a mean difference of 3.7 %. However, the latter approach, albeit extensively used [51, 79], does not take into account possible changes in the size of the elements within single sections, and therefore can be less precise than the smoothed upper bound surface. Moreover, segmenting the apposed surface with the *area lists brush* is faster and easier than using the *profile tool*. Thus, all the interfacing surfaces were segmented using the *area lists brush* and estimated according to the smoothed upper bound formula. ASI in spines with perforated synapses and spines with synapses obliquely or orthogonally oriented to the cutting plane was not segmented.

The total astrocytic volume in the neuropil was measured by segmenting all the astrocytic profiles in a region of interest (ROI) delineated around each segmented spine. ROIs did not include large dendrites or somata of neurons, glia or endothelial cells. For each ROI, the ROI volume, the astrocytic volume, and the ratio between the astrocytic surface and the astrocytic volume were estimated.

#### Availability of supporting data

The microarray data set supporting the results of this article is available in the NCBI Gene Expression Omnibus (GEO) repository, and accessible through the GEO Series accession number GSE69079 [80]. The SBF-SEM spine data will be publicly released in conjunction with a future publication currently in preparation and focusing on the spine changes.

## Additional files

Additional file 1: Table S1. Astrocyte genes upregulated because of time of day (3 am) (fold change >30 %, *P* < 0.01). (PDF 364 kb) Additional file 2: Table S2. Astrocyte genes upregulated because of time of day (3 pm) (fold change >30 %, *P* < 0.01). (PDF 256 kb) Additional file 3: Table S3. Astrocyte genes upregulated in S vs W + SD (fold change >30 %, *P* < 0.01). (PDF 277 kb) Additional file 4: Table S4. Astrocyte genes upregulated in W + SD vs S (fold change >30 %, *P* < 0.01). (PDF 1105 kb) Additional file 5: Table S5. Functional clusters for sleep genes. (PDF 188 kb) Additional file 6: Table S6. Functional clusters for wake genes. (PDF 256 kb) Additional file 7: Table S7. Astrocyte genes upregulated in SD vs W and W vs SD (fold change >30 %, *P* < 0.01). (PDF 701 kb) Additional file 8: Figure S1. Functional characterization of genes differentially expressed in wake (W) and sleep deprivation (SD). Functional annotation analysis (DAVID, default settings, except for kappa = 4, similarly threshold = 0.75) for W (*n* = 200) and SD (*n* = 384) genes. The top ten functional annotation clusters in order of enrichment score are shown for W (*top*) and SD (*bottom*). (TIFF 137 kb) Additional file 9: Table S8. Raw astrocytic EM measurements in S, SD, CSR, and W. (XLS 243 kb) Additional file 10: Figure S2. PAPs move closer to the synaptic cleft after spontaneous wake. Percentage of PAP^+ASI–^ and PAP^+ASI+^ spines per dendrite in layer II cortical fields for S (*n* = 9 dendrites; PAP^+ASI–^, 31.1 ± 9.6 %; PAP^+ASI+^, 51.09 ± 7.88 %), W (*n* = 8 dendrites; PAP^+ASI–^, 18.2 ± 8.45 %; PAP^+ASI+^, 68.23 ± 6.27 %). PAP^+ASI–^, S vs W, MW, **P* = 0.019; PAP^+ASI+^, S vs W, MW, **P* = 0.0002. All values are mean ± standard deviation. (TIFF 52 kb)

## Abbreviations

ALDH1L1: 10-formyltetrahydrofolate dehydrogenase
ASI: axon–spine interface
BAC: bacterial artificial chromosome
CSR: chronic sleep restriction
Cx-30: connexin30
DSB: double-strand break
EEG: electroencephalographic
eGFP: enhanced green fluorescent protein
EMG: electromyogram
GFAP: glial fibrillary acidic protein
IP: immunoprecipitated
MO: mature oligodendrocyte
MW: Mann–Whitney test
NGS: normal goat serum
NREM: non-rapid eye movement
OPC: oligodendrocyte precursor cell
PAP: peripheral astrocytic process
PB: phosphate buffer
qPCR: quantitative polymerase chain reaction
REM: rapid eye movement
ROI: region of interest
S: sleeping mouse
SBF-SEM: serial block face scanning electron microscopy
SD: sleep-deprived mouse
SWA: slow-wave activity
TRAP: translating ribosome affinity purification
UB: unbound
W: awake mouse
