# Supplementary material for: Effects of sleep and wake on astrocytes: clues from molecular and ultrastructural studies
Source: BMC Biol. 2015 Aug 25;13:66. doi: 10.1186/s12915-015-0176-7 (PMC4548305; doi:10.1186/s12915-015-0176-7)
Supplement: Additional file 1: Table S1. — Astrocyte genes upregulated because of time of day (3 am) (fold change >30 %, P < 0.01). (PDF 364 kb) [file 12915_2015_176_MOESM1_ESM.pdf]

# Table 1. Astrocyte genes upregulated because of time of day (3 am) (fold change>30%, p<0.01)

| | | | W vs. S+SD (3 am vs. 3 pm) | | W vs. S | | | W vs. SD | | |
|---|---|---|---|---|---|---|---|---|---|---|
| **Probeset ID** | **Gene Name** | **Gene Title** | **Average Log Fold Change** | **Average Expression** | **Log Fold Change** | **t** | **adj.P.Val** | **Log Fold Change** | **t** | **adj.P.Val** |
| 1444559_at | Phtf2 | Putative homeodomain transcription factor 2 | -1.79 | 4.06 | -1.80 | -6.62 | 0.0005 | -1.79 | -6.59 | 0.0007 |
| 1447552_s_at | Unknown | Unknown | -1.75 | 3.24 | -1.97 | -6.77 | 0.0004 | -1.53 | -5.24 | 0.0043 |
| 1446761_at | D8Ertd56e | DUnknown segment, Chr 8, ERATO Doi 56, expressed | -1.74 | 3.33 | -1.80 | -8.84 | 0.0000 | -1.68 | -8.23 | 0.0001 |
| 1458620_at | Gm5129 | predicted gene 5129 | -1.65 | 5.38 | -1.69 | -7.26 | 0.0002 | -1.60 | -6.85 | 0.0005 |
| 1422530_at | Prph | peripherin | -1.62 | 4.06 | -1.78 | -6.87 | 0.0003 | -1.45 | -5.58 | 0.0028 |
| 1435560_at | Itgal | integrin alpha L | -1.60 | 3.85 | -2.10 | -12.95 | 0.0000 | -1.10 | -6.79 | 0.0005 |
| 1429955_at | 5031434O11Rik | RIKEN cDNA 5031434O11 gene | -1.57 | 4.48 | -1.12 | -5.72 | 0.0016 | -2.03 | -10.41 | 0.0000 |
| 1445505_at | Ndst1 | N-deacetylase/N-sulfotransferase (heparan glucosaminyl) 1 | -1.47 | 3.59 | -1.64 | -7.27 | 0.0002 | -1.29 | -5.71 | 0.0023 |
| 1444207_at | Alms1 | Alstrom syndrome 1 homolog (human) | -1.46 | 3.63 | -1.67 | -7.03 | 0.0003 | -1.24 | -5.20 | 0.0046 |
| 1440862_at | Unknown | Unknown | -1.42 | 4.48 | -1.46 | -5.96 | 0.0012 | -1.39 | -5.65 | 0.0025 |
| 1459255_at | Unknown | Unknown | -1.39 | 3.17 | -1.44 | -7.70 | 0.0001 | -1.35 | -7.19 | 0.0003 |
| 1438515_at | Zfp207 | zinc finger protein 207 | -1.30 | 4.67 | -1.36 | -5.69 | 0.0017 | -1.25 | -5.24 | 0.0043 |
| 1456681_at | Ptchd1 | patched domain containing 1 | -1.28 | 6.05 | -1.40 | -7.75 | 0.0001 | -1.17 | -6.49 | 0.0008 |
| 1430330_at | Wiz | widely-interspaced zinc finger motifs | -1.27 | 3.53 | -1.33 | -6.76 | 0.0004 | -1.20 | -6.07 | 0.0013 |
| 1446175_at | Unknown | Unknown | -1.22 | 3.48 | -1.32 | -10.50 | 0.0000 | -1.12 | -8.90 | 0.0001 |
| 1425669_at | Mobkl2b | MOB1, Mps One Binder kinase activator-like 2B (yeast) | -1.19 | 3.94 | -0.77 | -4.73 | 0.0063 | -1.61 | -9.92 | 0.0000 |
| 1444269_at | Unknown | Unknown | -1.18 | 3.68 | -0.86 | -4.69 | 0.0067 | -1.49 | -8.11 | 0.0001 |
| 1431362_a_at | Smoc2 | SPARC related modular calcium binding 2 | -1.17 | 4.61 | -1.07 | -5.30 | 0.0028 | -1.27 | -6.29 | 0.0010 |
| 1453395_at | 5330403D14Rik | RIKEN cDNA 5330403D14 gene | -1.17 | 3.82 | -1.10 | -7.28 | 0.0002 | -1.24 | -8.22 | 0.0001 |
| 1437370_at | Sgol2 | shugoshin-like 2 (S. pombe) | -1.17 | 4.15 | -1.16 | -4.97 | 0.0045 | -1.17 | -5.01 | 0.0062 |
| 1421686_at | Npvf | neuropeptide VF precursor | -1.16 | 4.43 | -1.21 | -9.95 | 0.0000 | -1.11 | -9.11 | 0.0000 |
| 1427618_at | Cdh9 | cadherin 9 | -1.15 | 3.61 | -1.07 | -5.59 | 0.0019 | -1.23 | -6.43 | 0.0008 |
| 1458755_at | Unknown | Unknown | -1.14 | 3.04 | -1.29 | -9.21 | 0.0000 | -0.99 | -7.06 | 0.0004 |
| 1444308_at | Unknown | Unknown | -1.13 | 4.78 | -1.17 | -6.17 | 0.0009 | -1.09 | -5.74 | 0.0022 |
| 1441074_at | Unknown | Unknown | -1.12 | 4.40 | -1.05 | -6.86 | 0.0004 | -1.19 | -7.74 | 0.0002 |
| 1460073_at | Unknown | Unknown | -1.09 | 3.90 | -0.89 | -4.54 | 0.0084 | -1.29 | -6.57 | 0.0007 |
| 1420991_at | Ankrd1 | ankyrin repeat domain 1 (cardiac muscle) | -1.08 | 3.67 | -1.36 | -12.30 | 0.0000 | -0.80 | -7.20 | 0.0003 |
| 1435794_at | BC050254 | cDNA sequence BC050254 | -1.08 | 5.84 | -0.98 | -4.77 | 0.0060 | -1.18 | -5.75 | 0.0021 |
| 1427497_at | 2610015P09Rik | RIKEN cDNA 2610015P09 gene | -1.05 | 4.38 | -1.00 | -7.96 | 0.0001 | -1.10 | -8.77 | 0.0001 |
| 1448577_x_at | Syngr2 | synaptogyrin 2 | -1.04 | 7.39 | -0.93 | -4.52 | 0.0086 | -1.15 | -5.61 | 0.0026 |
| 1419717_at | Sema3e | sema domain, immunoglobulin domain (Ig), short basic domain, secreted | -1.03 | 3.23 | -1.16 | -6.24 | 0.0008 | -0.91 | -4.90 | 0.0073 |
| 1440048_at | Smarca5 | SWI/SNF related, matrix associated, actin dependent regulator of chromatin | -1.02 | 3.19 | -1.05 | -7.11 | 0.0003 | -1.00 | -6.72 | 0.0006 |
| 1439929_at | Unknown | Unknown | -1.02 | 2.96 | -1.18 | -11.05 | 0.0000 | -0.87 | -8.16 | 0.0001 |
| 1423473_at | Sept2 | septin 2 | -1.02 | 2.96 | -1.06 | -6.23 | 0.0008 | -0.98 | -5.77 | 0.0020 |
| 1457093_at | Gapvd1 | GTPase activating protein and VPS9 domains 1 | -1.02 | 3.58 | -0.88 | -6.14 | 0.0009 | -1.15 | -7.98 | 0.0002 |
| 1454361_at | 9230106L01Rik | RIKEN cDNA 9230106L01 gene | -1.01 | 3.28 | -0.87 | -5.10 | 0.0038 | -1.16 | -6.81 | 0.0005 |
| 1437326_x_at | Cela3b | chymotrypsin-like elastase family, member 3B | -1.00 | 3.86 | -1.30 | -11.87 | 0.0000 | -0.69 | -6.34 | 0.0009 |
| 1429402_at | Glt8d2 | glycosyltransferase 8 domain containing 2 | -0.99 | 5.41 | -1.12 | -6.26 | 0.0008 | -0.86 | -4.82 | 0.0082 |
| 1431038_at | Rassf4 | Ras association (RalGDS/AF-6) domain family member 4 | -0.97 | 3.34 | -0.98 | -5.66 | 0.0017 | -0.97 | -5.59 | 0.0027 |
| 1447080_at | Aco1 | aconitase 1 | -0.97 | 4.50 | -1.13 | -7.79 | 0.0001 | -0.81 | -5.60 | 0.0027 |
| 1427756_x_at | Igh-6 | Immunoglobulin heavy chain 6 (heavy chain of IgM) | -0.97 | 3.68 | -1.13 | -7.48 | 0.0002 | -0.81 | -5.39 | 0.0035 |
| 1444354_at | Unknown | Unknown | -0.97 | 4.69 | -1.06 | -10.52 | 0.0000 | -0.87 | -8.66 | 0.0001 |
| 1430436_at | Fam115a | family with sequence similarity 115, member A | -0.96 | 3.09 | -0.82 | -5.08 | 0.0038 | -1.10 | -6.78 | 0.0005 |
| 1431564_at | Bcar3 | breast cancer anti-estrogen resistance 3 | -0.95 | 3.60 | -1.02 | -6.53 | 0.0005 | -0.89 | -5.73 | 0.0022 |
| 1446628_at | Unknown | Unknown | -0.95 | 4.08 | -1.08 | -10.53 | 0.0000 | -0.83 | -8.08 | 0.0001 |
| 1449572_at | Trhr | thyrotropin releasing hormone receptor | -0.94 | 3.24 | -1.03 | -5.97 | 0.0012 | -0.85 | -4.92 | 0.0070 |
| 1423714_at | Asf1b | ASF1 anti-silencing function 1 homolog B (S. cerevisiae) | -0.91 | 5.10 | -1.09 | -7.33 | 0.0002 | -0.74 | -5.01 | 0.0061 |
| 1431263_at | Prkag2 | protein kinase, AMP-activated, gamma 2 non-catalytic subunit | -0.91 | 3.09 | -0.84 | -6.35 | 0.0007 | -0.99 | -7.51 | 0.0002 |
| 1440711_at | C630001G18Rik | RIKEN cDNA C630001G18 gene | -0.90 | 3.22 | -0.79 | -5.12 | 0.0036 | -1.01 | -6.58 | 0.0007 |
| 1445149_at | Pvr | Poliovirus receptor | -0.89 | 4.11 | -0.98 | -8.43 | 0.0001 | -0.80 | -6.87 | 0.0005 |
| 1422942_at | Galr2 | galanin receptor 2 | -0.88 | 3.97 | -0.77 | -4.51 | 0.0088 | -0.99 | -5.83 | 0.0019 |
| 1417878_at | E2f1 | E2F transcription factor 1 | -0.88 | 6.82 | -0.87 | -4.58 | 0.0080 | -0.89 | -4.72 | 0.0093 |
| 1447851_x_at | Atp10a | ATPase, class V, type 10A | -0.88 | 4.62 | -0.85 | -5.18 | 0.0033 | -0.90 | -5.50 | 0.0030 |
| 1430299_at | Tsc22d4 | TSC22 domain family, member 4 | -0.87 | 6.75 | -0.94 | -5.88 | 0.0013 | -0.79 | -4.95 | 0.0068 |

| | | | | | | | | | | |
|---|---|---|---|---|---|---|---|---|---|---|
| 1442467_at | Nav2 | neuron navigator 2 | -0.86 | 3.90 | -1.05 | -8.60 | 0.0001 | -0.68 | -5.58 | 0.0028 |
| 1441155_at | Unknown | Unknown | -0.86 | 3.24 | -0.99 | -8.64 | 0.0001 | -0.72 | -6.30 | 0.0010 |
| 1443121_at | Calcoco1 | Calcium binding and coiled coil domain 1 | -0.84 | 4.47 | -1.02 | -7.90 | 0.0001 | -0.67 | -5.24 | 0.0043 |
| 1441441_at | Prrg1 | proline rich Gla (G-carboxyglutamic acid) 1 | -0.84 | 3.33 | -0.78 | -4.75 | 0.0062 | -0.91 | -5.57 | 0.0028 |
| 1447642_x_at | Dmwd | dystrophia myotonica-containing WD repeat motif | -0.84 | 3.13 | -0.94 | -7.20 | 0.0002 | -0.75 | -5.73 | 0.0022 |
| 1446751_s_at | Impact | imprinted and ancient | -0.84 | 6.55 | -0.80 | -5.20 | 0.0032 | -0.89 | -5.81 | 0.0019 |
| 1418286_a_at | Efnb1 | ephrin B1 | -0.82 | 6.97 | -0.93 | -6.29 | 0.0008 | -0.70 | -4.73 | 0.0092 |
| 1442331_at | Unknown | Unknown | -0.81 | 3.72 | -0.73 | -6.67 | 0.0005 | -0.88 | -8.01 | 0.0001 |
| 1432578_at | Naif1 | nuclear apoptosis inducing factor 1 | -0.79 | 5.89 | -0.88 | -7.81 | 0.0001 | -0.71 | -6.30 | 0.0010 |
| 1458058_at | 7030407E18Rik | RIKEN cDNA 7030407E18 gene | -0.79 | 3.76 | -0.90 | -6.64 | 0.0005 | -0.68 | -5.01 | 0.0062 |
| 1449866_at | Syt2 | synaptotagmin II | -0.78 | 4.90 | -0.77 | -5.22 | 0.0031 | -0.78 | -5.27 | 0.0041 |
| 1432626_at | 5730507A11Rik | RIKEN cDNA 5730507A11 gene | -0.77 | 4.00 | -0.75 | -7.43 | 0.0002 | -0.79 | -7.80 | 0.0002 |
| 1424493_s_at | Ugt3a1 | UDP glycosyltransferases 3 family, polypeptide A1 | -0.77 | 4.08 | -0.97 | -9.78 | 0.0000 | -0.56 | -5.61 | 0.0026 |
| 1442081_at | Gm4632 | Predicted gene 4632 | -0.77 | 4.51 | -0.66 | -5.15 | 0.0035 | -0.87 | -6.75 | 0.0005 |
| 1432500_at | 2410006F04Rik | RIKEN cDNA 2410006F04 gene | -0.75 | 2.93 | -0.71 | -6.57 | 0.0005 | -0.79 | -7.27 | 0.0003 |
| 1451534_at | Scgn | secretagogin, EF-hand calcium binding protein | -0.75 | 4.47 | -0.90 | -7.19 | 0.0002 | -0.60 | -4.82 | 0.0082 |
| 1442573_at | Unknown | Unknown | -0.75 | 3.22 | -0.77 | -7.40 | 0.0002 | -0.72 | -6.95 | 0.0004 |
| 1422802_at | Defa3 | defensin, alpha, 3 | -0.75 | 3.77 | -0.73 | -5.29 | 0.0029 | -0.76 | -5.55 | 0.0029 |
| 1432281_a_at | Itgb6 | integrin beta 6 | -0.74 | 2.94 | -0.95 | -9.99 | 0.0000 | -0.53 | -5.56 | 0.0028 |
| 1417852_x_at | Clca1 | chloride channel calcium activated 1 | -0.74 | 2.81 | -0.63 | -5.73 | 0.0016 | -0.85 | -7.79 | 0.0002 |
| 1455838_at | Slfnl1 | schlafen like 1 | -0.74 | 4.99 | -0.96 | -8.81 | 0.0000 | -0.52 | -4.72 | 0.0093 |
| 1451951_at | Igkv14-111 | Immunoglobulin kappa chain variable 14-111 | -0.74 | 3.84 | -0.74 | -7.59 | 0.0001 | -0.73 | -7.46 | 0.0003 |
| 1446813_s_at | D5Ertd560e | DUnknown segment, Chr 5, ERATO Doi 560, expressed | -0.74 | 3.09 | -0.79 | -8.26 | 0.0001 | -0.68 | -7.04 | 0.0004 |
| 1442405_at | Csrp2bp | Cysteine and glycine-rich protein 2 binding protein | -0.73 | 3.62 | -0.84 | -7.73 | 0.0001 | -0.62 | -5.68 | 0.0023 |
| 1442228_at | Unknown | Unknown | -0.73 | 3.45 | -0.44 | -4.45 | 0.0095 | -1.02 | -10.26 | 0.0000 |
| 1436932_at | Grhl3 | grainyhead-like 3 (Drosophila) | -0.71 | 3.55 | -0.72 | -4.90 | 0.0049 | -0.70 | -4.76 | 0.0089 |
| 1459892_at | Psg19 | pregnancy specific glycoprotein 19 | -0.71 | 2.57 | -0.72 | -6.97 | 0.0003 | -0.69 | -6.68 | 0.0006 |
| 1430653_at | A930026B05Rik | RIKEN cDNA A930026B05 gene | -0.71 | 4.61 | -0.61 | -4.49 | 0.0090 | -0.81 | -5.97 | 0.0015 |
| 1444932_at | 4732419C18Rik | RIKEN cDNA 4732419C18 gene | -0.71 | 4.83 | -0.73 | -5.07 | 0.0038 | -0.68 | -4.67 | 0.0100 |
| 1443025_at | Unknown | Unknown | -0.70 | 3.31 | -0.70 | -4.98 | 0.0044 | -0.70 | -4.99 | 0.0064 |
| 1420061_s_at | AA675344 | expressed sequence AA675344 | -0.70 | 2.64 | -0.79 | -6.51 | 0.0006 | -0.62 | -5.09 | 0.0054 |
| 1440124_at | B230334C09Rik | RIKEN cDNA B230334C09 gene | -0.70 | 3.42 | -0.75 | -5.95 | 0.0012 | -0.65 | -5.16 | 0.0049 |
| 1438779_at | Col4a3 | collagen, type IV, alpha 3 | -0.70 | 3.44 | -0.70 | -5.52 | 0.0021 | -0.70 | -5.50 | 0.0030 |
| 1458495_at | Unknown | Unknown | -0.69 | 4.20 | -0.93 | -9.54 | 0.0000 | -0.46 | -4.74 | 0.0091 |
| 1432751_at | 4930403O18Rik | RIKEN cDNA 4930403O18 gene | -0.69 | 3.59 | -0.79 | -6.75 | 0.0004 | -0.59 | -5.04 | 0.0059 |
| 1447245_at | Unknown | Unknown | -0.69 | 4.19 | -0.84 | -7.78 | 0.0001 | -0.54 | -4.96 | 0.0066 |
| 1420784_at | Scn11a | sodium channel, voltage-gated, type XI, alpha | -0.67 | 3.84 | -0.68 | -4.91 | 0.0048 | -0.66 | -4.77 | 0.0087 |
| 1459735_at | Fkbp15 | FK506 binding protein 15 | -0.66 | 4.03 | -0.74 | -6.22 | 0.0008 | -0.58 | -4.82 | 0.0082 |
| 1444747_at | Unknown | Unknown | -0.66 | 2.76 | -0.78 | -8.43 | 0.0001 | -0.54 | -5.87 | 0.0018 |
| 1447134_at | Unknown | Unknown | -0.66 | 3.59 | -0.59 | -4.53 | 0.0085 | -0.72 | -5.51 | 0.0030 |
| 1442629_at | Unknown | Unknown | -0.66 | 3.61 | -0.74 | -6.14 | 0.0009 | -0.58 | -4.78 | 0.0086 |
| 1429858_at | 1700011E24Rik | RIKEN cDNA 1700011E24 gene | -0.62 | 4.39 | -0.57 | -5.66 | 0.0017 | -0.67 | -6.61 | 0.0007 |
| 1444536_at | Unknown | Unknown | -0.61 | 3.65 | -0.68 | -5.91 | 0.0013 | -0.54 | -4.70 | 0.0096 |
| 1446027_at | Unknown | Unknown | -0.61 | 4.45 | -0.56 | -4.48 | 0.0091 | -0.67 | -5.37 | 0.0036 |
| 1450578_at | Sry | sex determining region of Chr Y | -0.60 | 3.14 | -0.65 | -5.85 | 0.0014 | -0.54 | -4.89 | 0.0074 |
| 1442485_at | Unknown | Unknown | -0.58 | 3.45 | -0.45 | -4.54 | 0.0084 | -0.71 | -7.11 | 0.0004 |
| 1449081_at | Ces3 | carboxylesterase 3 | -0.57 | 2.57 | -0.56 | -6.02 | 0.0011 | -0.58 | -6.24 | 0.0011 |
| 1454129_at | 4933416M06Rik | RIKEN cDNA 4933416M06 gene | -0.54 | 4.67 | -0.54 | -4.89 | 0.0049 | -0.53 | -4.84 | 0.0081 |
| 1423147_at | Mat1a | methionine adenosyltransferase I, alpha | -0.53 | 3.38 | -0.62 | -6.92 | 0.0003 | -0.44 | -4.96 | 0.0067 |
