# Supplementary material for: Effects of sleep and wake on astrocytes: clues from molecular and ultrastructural studies
Source: BMC Biol. 2015 Aug 25;13:66. doi: 10.1186/s12915-015-0176-7 (PMC4548305; doi:10.1186/s12915-015-0176-7)
Supplement: Additional file 2: Table S2. — Astrocyte genes upregulated because of time of day (3 pm) (fold change >30 %, P < 0.01). (PDF 256 kb) [file 12915_2015_176_MOESM2_ESM.pdf]

Table 2. Astrocyte genes upregulated because of time of day (3 pm) (fold change>30%, p<0.01)

Probeset ID	Gene Name	Gene Title	S+SD vs. W (3 am vs. 3 pm)		S vs. W			SD vs. W		
			Average Log Fold Change	Average Expression	Log Fold Change	t	adj.P.Val	Log Fold Change	t	adj.P.Val
1456182_x_at	Mela	melanoma antigen	2.31	6.32	1.90	6.14	0.0009	2.73	8.81	0.0001
1424784_at	Gm13139	predicted gene 13139	2.08	4.96	2.19	6.82	0.0004	1.97	6.14	0.0012
1438750_at	Atrx	Alpha thalassemia/mental retardation syndrome X-linked homolog (human)	1.65	3.27	1.38	5.02	0.0042	1.92	6.97	0.0004
1458677_at	Entpd5	ectonucleoside triphosphate diphosphohydrolase 5	1.65	4.66	1.94	8.32	0.0001	1.35	5.80	0.0020
1449143_at	Rtp4	receptor transporter protein 4	1.48	5.31	1.31	5.76	0.0015	1.65	7.24	0.0003
1447224_at	Unknown	Unknown	1.20	3.49	1.29	8.83	0.0000	1.11	7.57	0.0002
1427054_s_at	Abi3bp	ABI gene family, member 3 (NESH) binding protein	1.19	3.03	1.46	7.61	0.0001	0.92	4.81	0.0082
1431046_at	Ppfia3	protein tyrosine phosphatase, receptor type, f polypeptide (PTPRF)	1.16	6.57	1.19	10.36	0.0000	1.12	9.76	0.0000
1439376_x_at	Dmtf1	cyclin D binding myb-like transcription factor 1	1.11	5.06	0.97	4.98	0.0044	1.25	6.40	0.0009
1455520_at	Ppp2r5c	protein phosphatase 2, regulatory subunit B (B56), gamma isoform	1.02	5.18	1.08	7.02	0.0003	0.96	6.25	0.0011
1440926_at	Flt1	FMS-like tyrosine kinase 1	1.02	4.08	0.96	4.80	0.0057	1.07	5.37	0.0036
1445182_at	Zfp672	Zinc finger protein 672	0.99	3.17	0.87	6.17	0.0009	1.11	7.88	0.0002
1446655_at	Scyl3	SCY1-like 3 (S. cerevisiae)	0.99	5.54	0.86	7.88	0.0001	1.11	10.15	0.0000
1431819_at	Unknown	Unknown	0.94	5.76	1.19	8.27	0.0001	0.68	4.73	0.0092
1432672_at	4933407A17Rik	RIKEN cDNA 4933407A17 gene	0.93	3.69	1.20	9.98	0.0000	0.67	5.55	0.0029
1442544_at	Igh-6	Immunoglobulin heavy chain 6 (heavy chain of IgM)	0.92	3.17	0.88	7.86	0.0001	0.97	8.58	0.0001
1422033_a_at	Cntf	ciliary neurotrophic factor	0.92	6.97	0.99	5.94	0.0012	0.84	5.04	0.0059
1445326_at	Sdk1	sidekick homolog 1 (chicken)	0.90	4.64	0.73	4.70	0.0067	1.08	6.98	0.0004
1437906_x_at	Txnl1	thioredoxin-like 1	0.90	3.23	0.54	5.13	0.0036	1.26	12.05	0.0000
1426925_at	Rc3h2	ring finger and CCCH-type zinc finger domains 2	0.89	2.91	0.94	5.91	0.0013	0.84	5.25	0.0043
1439709_at	Unknown	Unknown	0.88	3.15	0.54	5.65	0.0017	1.23	12.90	0.0000
1457789_at	Cln3	ceroid lipofuscinosis, neuronal 3, juvenile (Batten, Spielmeyer-Vogt disease)	0.88	3.87	0.88	5.83	0.0014	0.88	5.79	0.0020
1432424_at	4930505H01Rik	RIKEN cDNA 4930505H01 gene	0.87	5.53	0.74	5.32	0.0028	1.00	7.23	0.0003
1440658_at	Ammecr1l	AMME chromosomal region gene 1-like	0.86	3.32	0.83	4.72	0.0065	0.90	5.08	0.0055
1458556_at	Unknown	Unknown	0.86	2.81	0.90	10.77	0.0000	0.82	9.73	0.0000
1425559_a_at	Acsm3	acyl-CoA synthetase medium-chain family member 3	0.85	6.14	0.97	7.33	0.0002	0.73	5.57	0.0028
1454762_at	Xkrx	X Kell blood group precursor related X linked	0.84	5.42	0.78	5.30	0.0028	0.91	6.15	0.0012
1421173_at	Irf4	interferon regulatory factor 4	0.83	3.56	0.85	5.12	0.0036	0.80	4.82	0.0082
1453841_at	2310050P20Rik	RIKEN cDNA 2310050P20 gene	0.81	2.80	0.52	4.88	0.0051	1.09	10.28	0.0000
1438793_x_at	Ergic1	endoplasmic reticulum-golgi intermediate compartment (ERGIC) 1	0.78	3.24	0.54	5.27	0.0029	1.02	9.90	0.0000
1454262_at	Fbxw27	F-box and WD-40 domain protein 27	0.77	3.74	0.78	7.14	0.0002	0.76	6.96	0.0004
1416155_at	Hmgb3	high mobility group box 3	0.75	10.01	0.65	4.73	0.0064	0.84	6.12	0.0012
1444757_at	Unknown	Unknown	0.74	3.11	0.62	4.98	0.0044	0.86	6.92	0.0004
1440114_x_at	Kank3	KN motif and ankyrin repeat domains 3	0.72	3.71	0.82	7.36	0.0002	0.62	5.63	0.0025
1424410_at	Ttc8	tetratricopeptide repeat domain 8	0.72	8.13	0.71	5.21	0.0032	0.72	5.26	0.0042
1456134_x_at	Yif1a	Yip1 interacting factor homolog A (S. cerevisiae)	0.71	3.51	0.58	6.69	0.0004	0.83	9.59	0.0000
1444642_at	Unknown	Unknown	0.70	4.26	0.54	4.47	0.0092	0.86	7.17	0.0003
1447492_at	Unknown	Unknown	0.69	3.67	0.75	6.28	0.0008	0.63	5.35	0.0037
1430182_a_at	1700006J14Rik	RIKEN cDNA 1700006J14 gene	0.67	2.75	0.71	6.10	0.0010	0.63	5.35	0.0037
1444023_at	Ank2	ankyrin 2, brain	0.65	2.45	0.62	4.69	0.0068	0.69	5.20	0.0046
1459225_at	Gnl3l	Guanine nucleotide binding protein-like 3 (nucleolar)-like	0.64	2.69	0.73	7.99	0.0001	0.55	6.07	0.0013
1450597_at	Olfr870	olfactory receptor 870	0.63	3.35	0.53	4.53	0.0084	0.73	6.16	0.0012
1420164_at	D7Ertd183e	DUnknown segment, Chr 7, ERATO Doi 183, expressed	0.63	3.48	0.51	4.93	0.0047	0.74	7.11	0.0004
1440935_at	Unknown	Unknown	0.59	4.48	0.55	5.90	0.0013	0.63	6.76	0.0005
1429991_at	Fezf1	Fez family zinc finger 1	0.59	4.07	0.66	7.03	0.0003	0.51	5.35	0.0037
1420161_at	AA409749	expressed sequence AA409749	0.56	4.11	0.61	7.07	0.0003	0.52	6.03	0.0014
1437425_at	Gdap1	ganglioside-induced differentiation-associated-protein 1	0.55	4.85	0.49	5.02	0.0042	0.61	6.24	0.0011
1459097_at	Unknown	Unknown	0.55	3.38	0.54	4.95	0.0046	0.56	5.19	0.0047
1445187_at	9430070O13Rik	RIKEN cDNA 9430070O13 gene	0.53	2.60	0.58	5.63	0.0018	0.48	4.69	0.0097
