# Supplementary material for: Effects of sleep and wake on astrocytes: clues from molecular and ultrastructural studies
Source: BMC Biol. 2015 Aug 25;13:66. doi: 10.1186/s12915-015-0176-7 (PMC4548305; doi:10.1186/s12915-015-0176-7)
Supplement: Additional file 3: Table S3. — Astrocyte genes upregulated in S vs W + SD (fold change >30 %, P < 0.01). (PDF 277 kb) [file 12915_2015_176_MOESM3_ESM.pdf]

Table 3. Astrocyte genes upregulated in S *vs* W + SD (fold change>30%, p<0.01)

Probeset ID	Gene Name	Gene Title	S vs. W + SD		S vs. W			S vs. SD		
			Average Log Fold Change	Average Expression	Log Fold Change	t	adj.P.Val	Log Fold Change	t	adj.P.Val
1419728_at	Cxcl5	chemokine (C-X-C motif) ligand 5	1.86	3.93	2.05	8.37	6.23E-05	1.68	6.83	2.41E-04
1419833_s_at	Arap3	ArfGAP with RhoGAP domain, ankyrin repeat and PH domain 3	1.83	4.14	1.57	5.37	2.54E-03	2.09	7.18	1.61E-04
1447962_at	Unknown	Unknown	1.82	3.45	1.75	6.25	8.14E-04	1.89	6.75	0.000269
1441048_at	5930430L01Rik	RIKEN cDNA 5930430L01 gene	1.75	5.23	1.84	5.29	0.002859	1.67	4.82	4.21E-03
1436447_at	A630026N12Rik	RIKEN cDNA A630026N12 gene	1.72	3.48	1.53	6.69	4.41E-04	1.91	8.37	3.60E-05
1429572_at	Nsun7	NOL1/NOP2/Sun domain family, member 7	1.65	4.50	1.40	5.30	2.84E-03	1.91	7.25	1.47E-04
1425772_at	Col4a4	collagen, type IV, alpha 4	1.53	4.28	1.37	7.76	1.22E-04	1.68	9.50	1.16E-05
1442590_at	Tnfrsf22	tumor necrosis factor receptor superfamily, member 22	1.51	3.53	1.91	7.48	0.000165	1.12	4.40	7.88E-03
1416332_at	Cirbp	cold inducible RNA binding protein	1.41	10.34	1.74	10.16	1.19E-05	1.09	6.36	0.00047
1427018_at	Tsnaxip1	translin-associated factor X (Tsnax) interacting protein 1	1.40	4.46	1.59	10.69	8.67E-06	1.20	8.12	5.06E-05
1453402_at	6430500C12Rik	RIKEN cDNA 6430500C12 gene	1.36	3.19	1.63	6.78	0.000391	1.09	4.54	6.31E-03
1417700_at	Rab38	RAB38, member of RAS oncogene family	1.33	4.23	1.06	5.24	0.003039	1.60	7.89	6.62E-05
1425008_a_at	Ifi203	interferon activated gene 203	1.30	3.02	1.42	5.29	0.002868	1.19	4.44	7.38E-03
1445234_at	C130030K03Rik	RIKEN cDNA C130030K03 gene	1.27	3.12	1.25	5.63	0.001762	1.29	5.81	1.00E-03
1446274_at	Slc16a1	solute carrier family 16 (monocarboxylic acid transporters), member 1	1.26	3.34	0.94	7.20	2.28E-04	1.59	12.25	1.31E-06
1445557_at	0610040B10Rik	RIKEN cDNA 0610040B10 gene	1.26	4.63	1.17	7.69	0.000134	1.35	8.90	2.05E-05
1431524_at	4933427I22Rik	RIKEN cDNA 4933427I22 gene	1.25	6.18	1.23	6.12	0.000969	1.27	6.36	4.70E-04
1450494_x_at	Ceacam1	carcinoembryonic antigen-related cell adhesion molecule 1	1.24	5.16	1.35	7.24	2.18E-04	1.14	6.14	0.000633
1428604_at	2610305D13Rik	RIKEN cDNA 2610305D13 gene	1.15	3.13	1.14	5.93	0.001243	1.16	6.08	6.85E-04
1420389_at	Pax3	paired box gene 3	1.13	3.09	1.21	5.31	0.002808	1.05	4.63	5.58E-03
1433814_at	Fbxl14	F-box and leucine-rich repeat protein 14	1.10	3.61	1.01	7.93	0.000102	1.20	9.43	1.25E-05
1430204_at	5033425G24Rik	RIKEN cDNA 5033425G24 gene	1.08	3.09	1.03	4.54	8.37E-03	1.14	5.01	0.003216
1436035_at	3830431G21Rik	RIKEN cDNA 3830431G21 gene	1.06	5.96	1.13	5.74	1.58E-03	0.99	5.05	3.03E-03
1417020_at	Spata4	spermatogenesis associated 4	1.06	2.84	0.99	4.43	0.009812	1.13	5.03	0.003111
1426230_at	Sphk2	sphingosine kinase 2	1.06	11.57	0.92	6.19	8.95E-04	1.19	8.02	5.6E-05
1420444_at	Slc22a3	solute carrier family 22 (organic cation transporter), member 3	1.04	5.03	1.12	5.48	2.18E-03	0.96	4.72	4.89E-03
1418174_at	Dbp	D site albumin promoter binding protein	1.01	11.20	0.98	5.56	1.95E-03	1.04	5.93	8.26E-04
1442767_s_at	Uba1	ubiquitin-like modifier activating enzyme 1	0.97	3.29	0.77	4.84	0.005361	1.18	7.43	1.20E-04
1453797_at	Phospho2	phosphatase, orphan 2	0.96	7.32	1.01	6.02	1.10E-03	0.91	5.47	1.63E-03
1430128_a_at	Reep6	receptor accessory protein 6	0.95	8.04	0.97	4.54	8.37E-03	0.93	4.36	8.35E-03
1449178_at	Pdlim3	PDZ and LIM domain 3	0.95	4.55	1.07	6.57	0.00052	0.83	5.11	2.78E-03
1418438_at	Fabp2	fatty acid binding protein 2, intestinal	0.92	3.11	1.05	8.03	9.33E-05	0.78	5.92	8.33E-04
1448975_s_at	Ren1	renin 1 structural	0.91	2.94	0.97	6.64	0.000471	0.85	5.77	0.001055
1456366_at	1700015F17Rik	RIKEN cDNA 1700015F17 gene	0.89	3.59	1.05	7.10	0.000253	0.74	4.98	3.35E-03
1438211_s_at	Dbp	D site albumin promoter binding protein	0.89	12.17	0.83	4.91	4.83E-03	0.94	5.58	1.40E-03
1432170_at	Tsfm	Ts translation elongation factor, mitochondrial	0.88	3.59	0.79	7.93	0.000102	0.98	9.83	8.08E-06
1440054_at	Pik3ca	phosphatidylinositol 3-kinase, catalytic, alpha polypeptide	0.87	5.53	0.82	5.07	0.003849	0.93	5.74	0.001109
1432672_at	4933407A17Rik	RIKEN cDNA 4933407A17 gene	0.86	3.69	1.20	9.98	1.42E-05	0.53	4.43	7.56E-03
1446473_at	4732418C07Rik	RIKEN cDNA 4732418C07 gene	0.86	2.73	0.93	7.49	0.000165	0.80	6.45	0.000412
1439207_at	Pnma5	paraneoplastic antigen family 5	0.86	3.48	0.75	5.31	0.002808	0.97	6.85	0.000239
1460490_at	Mrpl15	mitochondrial ribosomal protein L15	0.82	2.72	0.83	4.45	0.009554	0.81	4.32	8.86E-03
1444596_at	Pax7	paired box gene 7	0.70	3.23	0.74	4.92	4.78E-03	0.66	4.43	7.56E-03
1439193_at	Prrxl1	paired related homeobox protein-like 1	0.69	3.87	0.81	6.54	5.39E-04	0.57	4.61	5.72E-03
1429905_at	Lhx9	LIM homeobox protein 9	0.69	2.37	0.80	6.19	0.000885	0.57	4.35	8.44E-03
1457640_x_at	Pigs	phosphatidylinositol glycan anchor biosynthesis, class S	0.68	3.76	0.62	5.19	3.27E-03	0.73	6.11	6.54E-04
1434588_x_at	Gm6142	tubulin cofactor a pseudogene	0.68	4.65	0.59	6.04	1.07E-03	0.77	7.89	6.62E-05
1423854_a_at	Rasl11b	RAS-like, family 11, member B	0.67	10.08	0.68	5.23	3.09E-03	0.66	5.04	3.07E-03
1427327_at	Pilra	paired immunoglobin-like type 2 receptor alpha	0.65	3.26	0.73	5.87	0.001344	0.57	4.55	0.006283
1448470_at	Fbp1	fructose bisphosphatase 1	0.65	2.58	0.67	4.64	7.24E-03	0.63	4.34	8.68E-03
1460115_at	Unknown	Unknown	0.64	5.33	0.81	7.36	1.92E-04	0.48	4.39	7.94E-03
1424233_at	Meox2	mesenchyme homeobox 2	0.62	2.72	0.55	6.18	0.000897	0.69	7.83	7.08E-05
1450810_at	Fshr	follicle stimulating hormone receptor	0.62	3.76	0.60	5.00	0.004286	0.63	5.23	0.00233
1450496_a_at	Ska1	spindle and kinetochore associated complex subunit 1	0.60	2.48	0.60	4.44	0.009691	0.60	4.47	7.09E-03
1419626_at	Adam25	a disintegrin and metallopeptidase domain 25 (testase 2)	0.57	2.12	0.55	4.49	0.008988	0.59	4.78	0.004496

1442361_at	Park2	Parkinson disease (autosomal recessive, juvenile) 2, parkin	0.57	3.15	0.64	6.44	6.26E-04	0.49	4.94	0.003527
1415779_s_at	Actg1	actin, gamma, cytoplasmic 1	0.45	12.56	0.38	4.65	0.007229	0.52	6.28	5.21E-04
1446617_at	St8sia4	ST8 alpha-N-acetyl-neuraminide alpha-2,8-sialyltransferase 4	0.44	2.56	0.40	4.77	0.005991	0.48	5.72	1.13E-03
1448012_at	3110053B16Rik	RIKEN cDNA 3110053B16 gene	0.44	2.98	0.49	5.47	2.18E-03	0.39	4.31	9.06E-03
