# Supplementary material for: Effects of sleep and wake on astrocytes: clues from molecular and ultrastructural studies
Source: BMC Biol. 2015 Aug 25;13:66. doi: 10.1186/s12915-015-0176-7 (PMC4548305; doi:10.1186/s12915-015-0176-7)
Supplement: Additional file 4: Table S4. — Astrocyte genes upregulated in W + SD vs S (fold change >30 %, P < 0.01). (PDF 1105 kb) [file 12915_2015_176_MOESM4_ESM.pdf]

Table 4. Astrocyte genes upregulated in W + SD *vs* S (fold change>30%, p<0.01)

			W + SD vs S.		W vs. S			SD vs. S		
Probeset ID	Gene Name	Gene Title	Average Log Fold Change	Average Expression	Log Fold Change	t	adj.P.Val	Log Fold Change	t	adj.P.Val
1427683_at	Egr2	early growth response 2	-3.02	6.46	-2.65	-4.83	0.005435	-3.39	-6.19	0.000587
1418687_at	Arc	activity regulated cytoskeletal-associated protein	-2.52	7.70	-2.32	-6.73	0.000412	-2.72	-7.90	6.6E-05
1435663_at	Esr1	estrogen receptor 1 (alpha)	-2.30	4.04	-1.64	-6.61	0.000492	-2.96	-11.93	1.54E-06
1458582_at	Lin54	lin-54 homolog (C. elegans)	-2.14	5.44	-1.74	-5.20	0.003223	-2.54	-7.56	1.00E-04
1416505_at	Nr4a1	nuclear receptor subfamily 4, group A, member 1	-2.07	7.86	-1.86	-6.76	3.98E-04	-2.28	-8.29	3.99E-05
1445394_at	Unknown	Unknown	-1.97	6.04	-1.87	-5.07	0.003866	-2.07	-5.60	1.36E-03
1418936_at	Maff	v-maf musculoaponeurotic fibrosarcoma oncogene family, protein F (avian)	-1.94	7.15	-1.53	-6.53	0.000539	-2.36	-10.04	7.15E-06
1458361_at	Dclre1c	DNA cross-link repair 1C, PSO2 homolog (S. cerevisiae)	-1.75	4.13	-1.46	-5.49	2.15E-03	-2.05	-7.74	7.77E-05
1445669_at	Spry4	sprouty homolog 4 (Drosophila)	-1.74	6.33	-1.59	-6.22	0.000846	-1.88	-7.34	0.000132
1438805_at	Ccnd3	cyclin D3	-1.68	5.77	-1.85	-8.64	5.06E-05	-1.52	-7.10	1.75E-04
1443475_at	Hist1h3i	histone cluster 1, H3i	-1.67	4.86	-1.79	-6.83	0.000364	-1.56	-5.94	8.17E-04
1417394_at	Klf4	Kruppel-like factor 4 (gut)	-1.66	5.47	-1.52	-4.91	0.004809	-1.80	-5.79	1.02E-03
1439261_x_at	Mitd1	MIT, microtubule interacting and transport, domain containing 1	-1.61	3.88	-2.00	-10.82	8.67E-06	-1.22	-6.60	3.33E-04
1459979_x_at	Zfp68	zinc finger protein 68	-1.58	5.04	-1.36	-4.98	0.004406	-1.81	-6.60	3.34E-04
1445456_at	Kcmf1	potassium channel modulatory factor 1	-1.58	4.12	-1.57	-15.26	3.56E-07	-1.59	-15.47	2.5E-07
1423509_a_at	Iapp	islet amyloid polypeptide	-1.57	4.36	-1.26	-7.62	1.47E-04	-1.89	-11.46	2.23E-06
1445339_at	Cnga4	cyclic nucleotide gated channel alpha 4	-1.56	3.94	-1.79	-6.86	0.000355	-1.33	-5.09	2.84E-03
1435560_at	Itgal	integrin alpha L	-1.55	3.85	-2.10	-12.95	2.65E-06	-1.00	-6.16	6.12E-04
1455721_at	Gspt2	G1 to S phase transition 2	-1.54	3.87	-1.69	-7.66	0.000139	-1.39	-6.30	5.02E-04
1417001_a_at	D4Wsu53e	DNA segment, Chr 4, Wayne State University 53, expressed	-1.53	8.46	-1.33	-7.67	0.000137	-1.73	-10.01	7.27E-06
1419766_at	Sik1	salt inducible kinase 1	-1.52	7.88	-1.11	-7.32	1.98E-04	-1.92	-12.66	1.15E-06
1422001_at	Inhbc	inhibin beta-C	-1.49	3.60	-1.44	-10.92	8.67E-06	-1.54	-11.68	1.74E-06
1460212_at	Gnat1	guanine nucleotide binding protein, alpha transducing 1	-1.49	4.34	-1.35	-8.16	8.09E-05	-1.63	-9.85	8.08E-06
1427345_a_at	Sult1a1	sulfotransferase family 1A, phenol-preferring, member 1	-1.47	9.56	-1.18	-4.60	7.82E-03	-1.76	-6.84	2.40E-04
1430314_at	RIKEN…	RIKEN cDNA 4933437F05 gene	-1.45	3.73	-1.35	-5.54	0.002015	-1.55	-6.34	4.77E-04
1427126_at	Hspa1b	heat shock protein 1B	-1.44	3.26	-1.59	-7.33	0.000198	-1.30	-5.98	7.76E-04
1423753_at	Bambi	BMP and activin membrane-bound inhibitor, homolog (Xenopus laevis)	-1.43	6.88	-1.28	-4.59	0.007875	-1.58	-5.66	1.24E-03
1421080_at	Nr4a3	nuclear receptor subfamily 4, group A, member 3	-1.42	8.25	-1.11	-4.91	0.004809	-1.72	-7.64	9.00E-05
1423026_at	Rad51c	RAD51 homolog c (S. cerevisiae)	-1.41	4.64	-1.49	-5.09	3.75E-03	-1.33	-4.55	0.006264
1432840_at	Galnt11	polypeptide N-acetylgalactosaminyltransferase 11	-1.40	5.43	-1.57	-6.06	0.001036	-1.24	-4.77	0.004563
1439647_at	Gm13298	predicted gene 13298	-1.39	5.52	-1.56	-8.64	5.06E-05	-1.21	-6.69	2.88E-04
1426063_a_at	Gem	GTP binding protein (gene overexpressed in skeletal muscle)	-1.38	8.36	-1.22	-5.46	2.25E-03	-1.55	-6.95	2.10E-04
1429221_at	Wdr85	WD repeat domain 85	-1.38	5.56	-1.44	-7.98	9.76E-05	-1.32	-7.31	1.36E-04
1455398_at	Lrrc8c	leucine rich repeat containing 8 family, member C	-1.38	7.45	-1.14	-6.24	0.000828	-1.62	-8.85	2.18E-05
1458387_at	Ptdss2	phosphatidylserine synthase 2	-1.36	6.40	-1.08	-5.06	0.003893	-1.65	-7.72	7.86E-05
1442428_at	Unknown	Unknown	-1.35	4.66	-1.33	-4.75	0.006223	-1.37	-4.88	0.003837
1417924_at	Pak3	p21 protein (Cdc42/Rac)-activated kinase 3	-1.35	4.61	-1.11	-5.17	3.38E-03	-1.59	-7.41	0.000123
1455472_at	A630071D13Rik	RIKEN cDNA A630071D13 gene	-1.35	4.36	-1.35	-4.57	0.008081	-1.35	-4.56	6.15E-03
1445343_at	Tmed5	transmembrane emp24 protein transport domain containing 5	-1.34	3.87	-0.90	-5.68	0.00169	-1.78	-11.24	2.54E-06
1446844_at	Chchd7	coiled-coil-helix-coiled-coil-helix domain containing 7	-1.33	3.56	-1.01	-4.91	4.81E-03	-1.66	-8.10	5.11E-05
1434741_at	Rreb1	ras responsive element binding protein 1	-1.32	3.88	-1.01	-9.27	2.86E-05	-1.64	-14.96	2.50E-07
1418322_at	Crem	cAMP responsive element modulator	-1.32	7.14	-0.86	-4.87	0.00513	-1.78	-10.15	6.82E-06
1428223_at	Mfsd2a	major facilitator superfamily domain containing 2A	-1.32	9.98	-1.29	-4.86	0.005137	-1.34	-5.06	0.00297
1429437_at	Prpf40a	PRP40 pre-mRNA processing factor 40 homolog A (yeast)	-1.31	3.88	-1.34	-5.79	0.001488	-1.29	-5.57	0.001416
1416811_s_at	Ctla2a	cytotoxic T lymphocyte-associated protein 2 alpha	-1.31	4.33	-1.21	-5.49	2.16E-03	-1.41	-6.37	4.65E-04
1426429_at	Thap7	THAP domain containing 7	-1.30	7.01	-1.21	-4.47	0.009253	-1.39	-5.15	2.61E-03
1457894_at	Mettl11b	Methyltransferase like 11B	-1.28	7.34	-1.17	-4.43	0.009812	-1.39	-5.23	2.32E-03
1429319_at	Rhoh	ras homolog gene family, member H	-1.27	4.66	-1.22	-7.47	1.66E-04	-1.32	-8.09	5.20E-05
1456771_at	Zer1	zer-1 homolog (C. elegans)	-1.27	3.22	-1.09	-7.55	0.000154	-1.45	-10.10	6.86E-06
1444675_at	AL023051	expressed sequence AL023051	-1.27	3.93	-1.10	-6.23	0.000837	-1.44	-8.20	4.59E-05
1440959_s_at	Mynn	myoneurin	-1.26	5.69	-1.08	-4.73	0.00631	-1.44	-6.30	0.000506
1427652_x_at	Synj2	synaptojanin 2	-1.26	8.07	-1.11	-4.80	0.005671	-1.41	-6.13	6.33E-04
1444705_at	App	amyloid beta (A4) precursor protein	-1.25	3.53	-1.28	-10.38	9.17E-06	-1.22	-9.91	7.81E-06
1434618_at	Crebzf	CREB/ATF bZIP transcription factor	-1.23	6.04	-1.23	-4.98	0.004432	-1.23	-4.98	3.36E-03

1449760_at	Ddost	dolichyl-di-phosphooligosaccharide-protein glycotransferase	-1.22	5.03	-1.25	-10.93	8.67E-06	-1.20	-10.43	5.63E-06
1432866_at	6030442K20Rik	RIKEN cDNA 6030442K20 gene	-1.22	3.59	-1.24	-5.61	1.83E-03	-1.20	-5.44	1.69E-03
1448471_a_at	Ctla2a	cytotoxic T lymphocyte-associated protein 2 alpha	-1.22	3.30	-1.17	-6.68	0.00045	-1.26	-7.18	0.000159
1450288_at	Cdh6	cadherin 6	-1.21	5.60	-1.04	-5.14	0.003534	-1.37	-6.74	0.000273
1456696_x_at	Rmi1	RMI1, RecQ mediated genome instability 1, homolog (S. cerevisiae)	-1.19	3.68	-0.95	-8.84	4.25E-05	-1.43	-13.38	6.22E-07
1423100_at	Fos	FBJ osteosarcoma oncogene	-1.19	8.08	-1.18	-4.53	0.008441	-1.20	-4.60	5.82E-03
1443168_at	Unknown	Unknown	-1.18	3.97	-1.22	-5.03	4.11E-03	-1.13	-4.64	5.57E-03
1433106_at	5830438M01Rik	RIKEN cDNA 5830438M01 gene	-1.17	4.42	-1.09	-6.02	0.001106	-1.25	-6.90	2.25E-04
1448594_at	Wisp1	WNT1 inducible signaling pathway protein 1	-1.17	4.13	-0.96	-5.13	0.00355	-1.39	-7.44	1.18E-04
1431844_at	Kcnmb2	potassium large conductance calcium-activated channel, subfamily M, beta member 2	-1.17	3.26	-1.29	-8.54	5.44E-05	-1.04	-6.84	0.00024
1437882_s_at	3110048L19Rik	zinc finger pseudogene	-1.16	4.07	-1.42	-7.43	0.000172	-0.89	-4.69	0.005171
1458034_at	RIKEN…	RIKEN cDNA 1200016B10 gene	-1.15	3.79	-1.27	-12.54	2.99E-06	-1.04	-10.31	6.39E-06
1431651_at	Ccdc151	coiled-coil domain containing 151	-1.15	5.01	-1.30	-7.98	9.76E-05	-1.00	-6.15	6.22E-04
1433099_at	4933428L12Rik	RIKEN cDNA 4933428L12 gene	-1.15	3.38	-1.39	-9.64	2.14E-05	-0.91	-6.31	4.96E-04
1427306_at	Ryr1	ryanodine receptor 1, skeletal muscle	-1.15	4.37	-1.22	-5.65	1.73E-03	-1.07	-4.96	3.44E-03
1444462_at	Unknown	Unknown	-1.15	3.72	-1.01	-7.32	0.000198	-1.28	-9.30	1.42E-05
1447534_at	Unknown	Unknown	-1.14	3.34	-1.14	-7.17	0.000231	-1.14	-7.13	0.000168
1458734_at	C78128	Expressed sequence C78128	-1.14	3.16	-1.19	-8.61	5.10E-05	-1.08	-7.80	7.38E-05
1427127_x_at	Hspa1b	heat shock protein 1B	-1.13	3.93	-1.13	-4.90	0.004891	-1.13	-4.87	3.92E-03
1450749_a_at	Nr4a2	nuclear receptor subfamily 4, group A, member 2	-1.13	7.59	-0.97	-5.15	0.003453	-1.29	-6.90	2.25E-04
1446257_at	Hs6st2	heparan sulfate 6-O-sulfotransferase 2	-1.13	4.21	-1.07	-4.59	0.007865	-1.18	-5.08	2.87E-03
1418220_at	Foxf2	forkhead box F2	-1.13	4.27	-1.31	-7.36	0.000191	-0.95	-5.33	1.99E-03
1430298_at	4930473A06Rik	RIKEN cDNA 4930473A06 gene	-1.11	3.52	-1.01	-7.60	1.47E-04	-1.22	-9.23	1.45E-05
1440716_at	6430604M11Rik	RIKEN cDNA 6430604M11 gene	-1.11	5.68	-1.17	-4.79	0.005785	-1.05	-4.33	0.008841
1442855_at	Dopey1	dopey family member 1	-1.11	4.12	-0.79	-6.88	0.000343	-1.43	-12.52	1.15E-06
1418099_at	Tnfrsf1b	tumor necrosis factor receptor superfamily, member 1b	-1.11	4.05	-0.96	-5.66	1.72E-03	-1.25	-7.33	1.33E-04
1430819_at	Naaa	N-acylethanolamine acid amidase	-1.10	3.50	-1.00	-5.06	0.003945	-1.21	-6.08	6.94E-04
1435998_at	Ccnb1ip1	cyclin B1 interacting protein 1	-1.10	3.42	-0.99	-4.46	0.009339	-1.21	-5.46	1.64E-03
1454428_at	4930467D19Rik	RIKEN cDNA 4930467D19 gene	-1.10	3.58	-1.06	-6.56	0.00052	-1.14	-7.03	1.92E-04
1443405_at	Unknown	Unknown	-1.10	5.38	-1.26	-9.28	2.86E-05	-0.95	-7.00	1.99E-04
1450807_at	Ltb4r2	leukotriene B4 receptor 2	-1.10	4.33	-0.77	-6.30	7.61E-04	-1.43	-11.69	1.74E-06
1427513_at	BC024137	cDNA sequence BC024137	-1.10	6.63	-0.81	-5.02	0.004144	-1.39	-8.64	2.64E-05
1450822_at	Lyz1	lysozyme 1	-1.10	3.49	-1.13	-6.42	0.000647	-1.06	-6.04	7.26E-04
1429777_at	Dnajb6	DnaJ (Hsp40) homolog, subfamily B, member 6	-1.09	4.73	-1.16	-8.71	4.70E-05	-1.02	-7.60	9.46E-05
1456846_at	Zbtb42	zinc finger and BTB domain containing 42	-1.09	5.62	-1.22	-5.80	0.00146	-0.95	-4.54	6.32E-03
1422122_at	Fcer2a	Fc receptor, IgE, low affinity II, alpha polypeptide	-1.08	4.27	-1.11	-9.27	2.86E-05	-1.05	-8.75	2.30E-05
1447804_x_at	BC031781	CDNA sequence BC031781	-1.07	3.30	-1.03	-7.54	0.000156	-1.12	-8.15	4.82E-05
1443008_at	Msi2	Musashi homolog 2 (Drosophila)	-1.07	4.09	-1.00	-7.52	0.000158	-1.13	-8.48	3.14E-05
1417214_at	Rab27b	RAB27b, member RAS oncogene family	-1.07	7.62	-0.95	-6.67	4.53E-04	-1.18	-8.24	4.27E-05
1457956_at	Camta1	calmodulin binding transcription activator 1	-1.07	3.67	-1.30	-9.55	2.32E-05	-0.83	-6.06	7.09E-04
1431906_at	2700068H02Rik	RIKEN cDNA 2700068H02 gene	-1.06	3.97	-1.09	-5.91	0.001279	-1.02	-5.53	1.50E-03
1433047_at	5330430B06Rik	RIKEN cDNA 5330430B06 gene	-1.05	3.57	-1.03	-10.74	8.67E-06	-1.08	-11.29	2.54E-06
1427836_at	0610007P08Rik	RIKEN cDNA 0610007P08 gene	-1.05	6.28	-0.98	-5.66	0.001724	-1.13	-6.55	3.58E-04
1456070_at	Ptprg	protein tyrosine phosphatase, receptor type, G	-1.05	6.82	-0.99	-5.28	0.002907	-1.10	-5.86	9.27E-04
1444318_at	Chchd7	Coiled-coil-helix-coiled-coil-helix domain containing 7	-1.05	5.90	-1.14	-7.79	0.000119	-0.96	-6.52	0.000372
1438099_at	Trio	triple functional domain (PTPRF interacting)	-1.05	8.69	-1.03	-6.07	0.00102	-1.06	-6.24	0.000547
1441742_at	Kcnh5	potassium voltage-gated channel, subfamily H (eag-related), member 5	-1.04	3.75	-1.18	-5.92	1.26E-03	-0.90	-4.51	6.65E-03
1427506_at	Ppil5	peptidylprolyl isomerase (cyclophilin) like 5	-1.03	5.38	-0.97	-8.09	8.64E-05	-1.09	-9.12	1.62E-05
1439613_at	Adcy10	adenylate cyclase 10	-1.03	3.95	-0.90	-8.53	5.44E-05	-1.17	-11.06	2.76E-06
1427880_at	Uqcrq	ubiquinol-cytochrome c reductase, complex III subunit VII	-1.03	4.21	-1.11	-5.42	2.36E-03	-0.96	-4.69	5.12E-03
1457339_at	Ccm2	cerebral cavernous malformation 2 homolog (human)	-1.02	4.33	-1.16	-8.77	4.33E-05	-0.88	-6.69	2.88E-04
1457842_at	Zfp292	zinc finger protein 292	-1.01	3.94	-0.89	-8.13	8.26E-05	-1.12	-10.25	6.58E-06
1445734_at	Tdh	L-threonine dehydrogenase; predicted gene 13929	-1.01	4.11	-0.83	-5.09	0.003754	-1.18	-7.23	0.00015
1443286_at	AU019754	expressed sequence AU019754	-1.00	4.67	-0.99	-9.28	2.86E-05	-1.02	-9.50	1.16E-05
1436090_at	Enpp6	ectonucleotide pyrophosphatase/phosphodiesterase 6	-1.00	5.04	-0.96	-5.66	1.73E-03	-1.04	-6.10	6.68E-04
1460275_at	Gpr3	G-protein coupled receptor 3	-1.00	6.67	-0.92	-5.53	0.002027	-1.08	-6.51	3.77E-04
1433352_at	C430049A07Rik	RIKEN cDNA C430049A07 gene	-0.99	4.24	-1.06	-8.21	7.69E-05	-0.93	-7.18	1.59E-04
1449015_at	Retnla	resistin like alpha	-0.99	3.96	-1.25	-10.45	8.96E-06	-0.73	-6.12	6.48E-04
1432623_at	Supv3l1	suppressor of var1, 3-like 1 (S. cerevisiae)	-0.98	5.52	-1.10	-6.42	0.000646	-0.87	-5.04	0.003073
1433404_at	4930423O20Rik	RIKEN cDNA 4930423O20 gene	-0.98	4.29	-1.07	-8.85	4.25E-05	-0.90	-7.42	0.000122
1447728_x_at	Hspa9	heat shock protein 9	-0.98	3.84	-1.00	-8.81	4.25E-05	-0.95	-8.35	3.67E-05
1429060_at	Malat1	metastasis associated lung adenocarcinoma transcript 1 (non-coding RNA)	-0.98	8.98	-1.04	-5.21	0.003177	-0.91	-4.55	6.26E-03
1454384_at	2900057C01Rik	RIKEN cDNA 2900057C01 gene	-0.98	3.60	-0.88	-8.40	6.23E-05	-1.07	-10.29	6.39E-06

1421044_at	Mrc2	mannose receptor, C type 2	-0.98	4.94	-1.08	-5.57	0.001934	-0.87	-4.45	7.32E-03
1438901_at	BC024814	cDNA sequence BC024814	-0.97	6.31	-0.88	-6.09	0.001003	-1.06	-7.36	1.29E-04
1459337_at	Alg6	asparagine-linked glycosylation 6 homolog (yeast, alpha-1,3,-glucosyltransferase)	-0.97	3.56	-1.11	-10.98	8.67E-06	-0.82	-8.11	5.11E-05
1424589_s_at	Rnpc3	RNA-binding region (RNP1, RRM) containing 3	-0.97	6.48	-0.82	-4.44	0.009648	-1.11	-6.00	0.000762
1420991_at	Ankrd1	ankyrin repeat domain 1 (cardiac muscle)	-0.96	3.67	-1.36	-12.30	3.05E-06	-0.57	-5.10	2.80E-03
1440748_at	Scarf1	scavenger receptor class F, member 1	-0.96	4.88	-0.93	-8.10	8.62E-05	-1.00	-8.69	2.48E-05
1419881_x_at	Unknown	Unknown	-0.96	3.59	-0.98	-5.12	0.003609	-0.94	-4.88	3.87E-03
1442705_at	C76938	expressed sequence C76938	-0.96	4.49	-0.90	-7.78	1.19E-04	-1.02	-8.81	2.23E-05
1437326_x_at	Cela3b	chymotrypsin-like elastase family, member 3B	-0.95	3.86	-1.30	-11.87	4.34E-06	-0.61	-5.53	1.50E-03
1420964_at	Enc1	ectodermal-neural cortex 1	-0.95	9.56	-0.90	-5.11	0.003664	-1.01	-5.77	0.001057
1430345_at	5530402H23Rik	RIKEN cDNA 5530402H23 gene	-0.95	2.99	-1.15	-8.86	4.25E-05	-0.76	-5.86	0.000927
1457984_at	Crh	corticotropin releasing hormone	-0.95	8.41	-0.97	-4.63	0.007394	-0.93	-4.45	7.35E-03
1443525_at	Dbx2	developing brain homeobox 2	-0.95	7.94	-0.95	-4.52	0.008704	-0.95	-4.51	6.67E-03
1447010_at	Zfp609	zinc finger protein 609	-0.95	4.09	-1.08	-10.61	8.77E-06	-0.82	-8.08	5.20E-05
1442014_at	Unknown	Unknown	-0.95	4.17	-1.10	-6.27	0.000788	-0.80	-4.59	5.94E-03
1417431_a_at	Sphk2	sphingosine kinase 2	-0.95	8.58	-0.93	-9.28	2.86E-05	-0.96	-9.54	1.12E-05
1440577_at	Mkln1	muskelin 1, intracellular mediator containing kelch motifs	-0.95	3.78	-0.70	-7.18	0.000229	-1.20	-12.31	1.31E-06
1440276_at	Rfpl4b	ret finger protein-like 4B	-0.95	4.04	-1.07	-9.13	3.07E-05	-0.82	-7.04	0.000191
1446684_at	Ralgapa1	Ral GTPase activating protein, alpha subunit 1	-0.94	3.89	-1.12	-9.16	2.99E-05	-0.77	-6.34	4.78E-04
1416814_at	Tia1	cytotoxic granule-associated RNA binding protein 1	-0.94	5.93	-0.93	-5.54	0.002022	-0.96	-5.71	1.15E-03
1424761_at	Fam115c	family with sequence similarity 115, member C	-0.94	4.49	-1.02	-5.12	0.003644	-0.85	-4.27	9.61E-03
1440111_at	Unknown	Unknown	-0.94	3.14	-0.77	-6.81	0.000378	-1.10	-9.70	9.01E-06
1422818_at	Nedd9	neural precursor cell expressed, developmentally down-regulated gene 9	-0.94	8.31	-0.77	-4.49	0.009019	-1.11	-6.44	4.17E-04
1432110_at	4930402F06Rik	RIKEN cDNA 4930402F06 gene	-0.94	3.83	-1.06	-7.21	2.25E-04	-0.81	-5.49	0.001577
1458509_at	Ankrd49	ankyrin repeat domain 49	-0.94	4.21	-1.22	-9.21	2.93E-05	-0.65	-4.89	0.003825
1449568_at	Klb	klotho beta	-0.94	3.20	-0.74	-5.49	0.002159	-1.13	-8.40	3.43E-05
1419808_at	Cog4	component of oligomeric golgi complex 4	-0.93	3.76	-0.99	-7.17	2.31E-04	-0.88	-6.37	4.68E-04
1447835_at	Fam125b	family with sequence similarity 125, member B	-0.93	5.25	-0.96	-4.74	0.006295	-0.90	-4.47	7.03E-03
1420527_s_at	Tcp10a	t-complex protein 10a	-0.93	4.16	-0.87	-6.58	0.000508	-0.99	-7.52	1.05E-04
1419410_at	Batf	basic leucine zipper transcription factor, ATF-like	-0.93	5.08	-0.96	-5.14	0.003534	-0.89	-4.79	4.45E-03
1448078_at	C76533	expressed sequence C76533	-0.93	3.96	-0.78	-6.17	0.000897	-1.08	-8.55	2.91E-05
1419024_at	Ptp4a1	protein tyrosine phosphatase 4a1	-0.92	6.92	-0.83	-5.27	2.93E-03	-1.02	-6.45	0.000411
1418614_at	Kcnj1	potassium inwardly-rectifying channel, subfamily J, member 1	-0.92	4.32	-0.95	-7.81	0.000118	-0.89	-7.31	1.37E-04
1436965_at	Emilin3	elastin microfibril interfacer 3	-0.91	4.37	-0.85	-6.85	0.000358	-0.98	-7.84	7.08E-05
1418221_at	Csn1s2b	casein alpha s2-like B	-0.91	2.75	-0.83	-7.66	1.39E-04	-1.00	-9.16	1.56E-05
1443202_at	Cyb5b	similar to cytochrome b5 outer mitochondrial membrane precursor; cytochrome b5 type B	-0.91	3.29	-0.69	-5.18	0.003306	-1.14	-8.55	2.92E-05
1458189_at	Emid2	EMI domain containing 2	-0.91	3.40	-0.76	-6.49	0.000574	-1.07	-9.17	1.56E-05
1419863_at	Tsdt2	cDNA sequence BC057893	-0.91	4.22	-0.94	-5.81	0.001446	-0.88	-5.43	0.001717
1441439_at	Ucn3	urocortin 3	-0.91	4.41	-0.84	-5.40	0.002431	-0.99	-6.36	0.000473
1415810_at	Uhrf1	ubiquitin-like, containing PHD and RING finger domains, 1	-0.91	4.85	-0.92	-4.81	5.62E-03	-0.90	-4.75	4.72E-03
1438542_at	Trp53	Transformation related protein 53	-0.91	3.87	-0.93	-7.21	0.000225	-0.89	-6.97	2.07E-04
1431718_at	Kif15	kinesin family member 15	-0.91	4.06	-0.76	-6.28	0.00078	-1.06	-8.84	2.20E-05
1422296_at	Sike1	suppressor of IKBKE 1	-0.91	4.10	-0.80	-8.93	4.03E-05	-1.01	-11.21	2.54E-06
1429911_at	Mcph1	microcephaly, primary autosomal recessive 1	-0.91	5.96	-0.90	-4.53	0.008439	-0.91	-4.58	5.98E-03
1431280_at	AI597468	expressed sequence AI597468	-0.90	7.35	-0.88	-5.77	0.001504	-0.92	-6.05	0.00072
1447188_at	Unknown	Unknown	-0.90	4.10	-0.74	-5.71	0.00162	-1.06	-8.11	5.11E-05
1439930_at	Smarca2	SWI/SNF related, matrix associated, actin dependent regulator of chromatin	-0.90	3.46	-1.13	-7.87	0.000109	-0.67	-4.69	5.18E-03
1422940_x_at	Serpinb3b	serine (or cysteine) peptidase inhibitor, clade B (ovalbumin), member 3B	-0.90	3.99	-0.77	-4.71	6.51E-03	-1.03	-6.34	4.77E-04
1444507_at	Usp53	ubiquitin specific peptidase 53	-0.90	8.20	-1.02	-5.71	0.001634	-0.77	-4.32	8.86E-03
1456016_x_at	BC005624	cDNA sequence BC005624	-0.90	3.58	-0.82	-6.14	0.000941	-0.97	-7.25	1.47E-04
1459823_at	Ehd2	EH-domain containing 2	-0.89	3.50	-0.59	-4.60	7.79E-03	-1.19	-9.27	1.44E-05
1444282_at	Samd10	Sterile alpha motif domain containing 10	-0.89	4.23	-0.75	-5.59	0.001883	-1.04	-7.74	7.77E-05
1421532_at	Rxfp2	relaxin/insulin-like family peptide receptor 2	-0.89	3.76	-0.84	-4.75	0.006223	-0.94	-5.30	0.002106
1442492_at	DXErtd11e	DNA segment, Chr X, ERATO Doi 11, expressed	-0.88	3.90	-0.87	-6.31	0.000757	-0.90	-6.58	3.42E-04
1438308_at	Agbl4	ATP/GTP binding protein-like 4	-0.88	4.03	-0.91	-8.32	6.61E-05	-0.85	-7.73	7.77E-05
1448830_at	Dusp1	dual specificity phosphatase 1	-0.88	10.72	-0.79	-5.57	1.94E-03	-0.97	-6.78	2.57E-04
1457111_at	AA415038	expressed sequence AA415038	-0.88	2.94	-0.92	-8.80	4.25E-05	-0.83	-7.95	6.14E-05
1454287_at	4931428A05Rik	RIKEN cDNA 4931428A05 gene	-0.87	4.04	-0.97	-7.49	0.000165	-0.77	-5.95	8.11E-04
1420004_s_at	Pom121	nuclear pore membrane protein 121	-0.87	7.91	-0.79	-6.04	1.07E-03	-0.96	-7.29	0.000139
1450645_at	Mt4	metallothionein 4	-0.87	4.04	-0.78	-6.56	0.00052	-0.97	-8.17	4.71E-05
1430679_at	4933404O12Rik	RIKEN cDNA 4933404O12 gene	-0.87	5.74	-0.99	-7.44	0.000171	-0.75	-5.61	1.33E-03
1458409_at	C86595	expressed sequence C86595	-0.87	3.62	-0.91	-8.62	5.1E-05	-0.83	-7.89	6.62E-05
1420503_at	Slc6a14	solute carrier family 6 (neurotransmitter transporter), member 14	-0.87	4.56	-1.01	-9.48	2.45E-05	-0.73	-6.81	2.47E-04

1439061_at	Ccdc84	coiled-coil domain containing 84	-0.87	6.20	-0.88	-5.18	3.32E-03	-0.85	-4.99	3.29E-03
1442360_at	Unknown	Unknown	-0.86	2.98	-0.97	-9.16	2.99E-05	-0.75	-7.08	1.81E-04
1450460_at	Aqp3	aquaporin 3	-0.86	4.53	-0.84	-5.16	0.003398	-0.89	-5.47	0.001628
1425918_at	Egln3	EGL nine homolog 3 (C. elegans)	-0.86	4.09	-0.63	-5.69	1.66E-03	-1.10	-9.90	7.81E-06
1454577_at	Cdc20b	cell division cycle 20 homolog B (S. cerevisiae)	-0.86	2.98	-0.48	-5.56	0.001953	-1.24	-14.31	3.47E-07
1431348_at	Ppifos	peptidylprolyl isomerase F, opposite strand transcription unit	-0.86	3.78	-0.90	-7.90	0.000104	-0.82	-7.20	1.57E-04
1433584_at	Gm5620	predicted gene 5620	-0.86	3.69	-0.69	-5.21	0.003177	-1.03	-7.76	7.69E-05
1429344_at	9130022E09	hypothetical 9130022E09	-0.86	3.53	-0.94	-6.01	0.00112	-0.77	-4.95	3.49E-03
1459490_at	Fam198b	RIKEN cDNA 1110032E23 gene	-0.85	3.95	-0.98	-7.19	2.29E-04	-0.73	-5.33	1.98E-03
1439386_x_at	Mat2a	methionine adenosyltransferase II, alpha	-0.85	9.54	-0.53	-4.43	0.009852	-1.17	-9.80	8.11E-06
1432687_at	4833406M21Rik	RIKEN cDNA 4833406M21 gene	-0.85	2.87	-0.86	-7.45	0.00017	-0.84	-7.34	0.000132
1436409_at	Cox8a	cytochrome c oxidase, subunit VIIIa	-0.85	3.48	-1.05	-8.00	9.76E-05	-0.65	-4.94	3.55E-03
1455261_at	Luc7l	Luc7 homolog (S. cerevisiae)-like	-0.85	6.97	-0.83	-4.97	0.004485	-0.87	-5.21	2.40E-03
1441639_at	Zcchc8	zinc finger, CCHC domain containing 8	-0.85	3.83	-0.90	-5.60	0.001841	-0.80	-4.94	3.52E-03
1441372_at	5930405F01Rik	RIKEN cDNA 5930405F01 gene	-0.85	3.86	-0.94	-8.19	7.78E-05	-0.75	-6.54	3.59E-04
1427862_at	LOC100047311	similar to protein translocation complex beta subunit	-0.85	5.63	-0.89	-6.52	0.000552	-0.81	-5.94	8.20E-04
1417950_a_at	Apoa2	apolipoprotein A-II	-0.85	6.63	-0.66	-4.54	8.37E-03	-1.03	-7.14	0.000168
1442911_at	Riok2	RIO kinase 2 (yeast)	-0.84	3.81	-0.81	-4.58	0.008035	-0.88	-4.99	0.003295
1449685_s_at	Oxsm	3-oxoacyl-ACP synthase, mitochondrial	-0.84	7.40	-0.81	-4.70	0.006584	-0.88	-5.08	2.86E-03
1452344_at	Synj2	synaptojanin 2	-0.84	8.00	-0.84	-5.16	3.45E-03	-0.84	-5.18	2.50E-03
1448804_at	Cyp11a1	cytochrome P450, family 11, subfamily a, polypeptide 1	-0.84	5.00	-0.92	-5.28	0.002879	-0.76	-4.36	8.40E-03
1429368_at	Lrig3	leucine-rich repeats and immunoglobulin-like domains 3	-0.84	3.39	-0.88	-7.97	9.76E-05	-0.80	-7.21	1.53E-04
1425258_at	Cst11	cystatin 11	-0.84	3.70	-1.01	-9.31	2.86E-05	-0.66	-6.06	7.09E-04
1422978_at	Cybb	cytochrome b-245, beta polypeptide	-0.84	3.87	-0.90	-6.47	0.00059	-0.77	-5.53	0.001503
1452676_a_at	Pnpt1	polyribonucleotide nucleotidyltransferase 1	-0.83	7.47	-0.76	-4.98	4.43E-03	-0.91	-5.96	0.000794
1428664_at	Vip	vasoactive intestinal polypeptide	-0.83	8.25	-0.88	-5.77	0.00151	-0.78	-5.09	2.83E-03
1457015_at	A230070E04Rik	RIKEN cDNA A230070E04 gene	-0.83	4.77	-0.92	-5.41	0.002415	-0.75	-4.40	7.88E-03
1457797_at	AI605517	expressed sequence AI605517	-0.83	3.11	-0.77	-4.97	4.45E-03	-0.89	-5.77	1.06E-03
1425025_at	Tmem106a	transmembrane protein 106A	-0.83	4.12	-0.65	-4.56	0.008153	-1.01	-7.09	1.79E-04
1452993_at	5430416O09Rik	RIKEN cDNA 5430416O09 gene	-0.83	4.59	-0.79	-7.00	0.000291	-0.86	-7.63	9.00E-05
1453503_at	Spink12	serine peptidase inhibitor, Kazal type 11	-0.83	3.74	-0.68	-5.33	0.002731	-0.98	-7.72	7.86E-05
1446350_at	Itga4	integrin alpha 4	-0.83	3.91	-0.97	-7.59	0.000147	-0.69	-5.41	0.001755
1420333_at	Txndc8	thioredoxin domain containing 8	-0.83	4.58	-0.78	-5.63	1.76E-03	-0.87	-6.28	5.21E-04
1433214_x_at	2700008E08Rik	RIKEN cDNA 2700008E08 gene	-0.82	4.93	-0.79	-5.08	0.003801	-0.86	-5.54	1.47E-03
1431481_at	4930443O20Rik	RIKEN cDNA 4930443O20 gene	-0.82	4.41	-0.85	-6.72	0.000425	-0.79	-6.23	5.52E-04
1432204_at	4930570B17Rik	RIKEN cDNA 4930570B17 gene	-0.82	3.23	-0.59	-5.11	3.65E-03	-1.05	-9.14	1.60E-05
1448539_a_at	Acy3	aspartoacylase (aminoacylase) 3	-0.82	7.64	-0.70	-4.92	0.004772	-0.94	-6.59	3.40E-04
1454482_at	5830474E16Rik	RIKEN cDNA 5830474E16 gene	-0.82	3.24	-0.92	-6.80	0.000381	-0.72	-5.35	0.001908
1436707_x_at	Ncaph	non-SMC condensin I complex, subunit H	-0.81	3.48	-0.72	-6.96	0.000305	-0.91	-8.82	2.21E-05
1458363_at	Zdhhc17	zinc finger, DHHC domain containing 17	-0.81	3.95	-0.65	-5.68	0.001679	-0.98	-8.57	2.88E-05
1419981_at	Riok3	RIO kinase 3 (yeast)	-0.81	8.21	-0.93	-6.18	8.97E-04	-0.70	-4.61	5.73E-03
1421314_at	Cttn	cortactin	-0.81	4.76	-0.73	-5.73	0.001585	-0.89	-6.94	2.13E-04
1441148_at	Fktn	fukutin	-0.81	4.46	-0.81	-4.87	0.005102	-0.80	-4.81	4.28E-03
1447668_x_at	Efemp2	epidermal growth factor-containing fibulin-like extracellular matrix protein 2	-0.81	6.08	-0.88	-5.99	1.15E-03	-0.74	-5.01	3.18E-03
1436654_at	Gen1	Gen homolog 1, endonuclease (Drosophila)	-0.81	4.49	-0.86	-5.18	0.003344	-0.76	-4.56	0.006147
1459932_at	D8Ertd28e	DNA segment, Chr 8, ERATO Doi 28, expressed	-0.80	3.28	-0.45	-4.76	0.006132	-1.15	-12.07	1.38E-06
1453618_at	4933424N20Rik	RIKEN cDNA 4933424N20 gene	-0.80	5.26	-0.87	-5.99	0.001144	-0.73	-5.03	3.08E-03
1433009_at	9030607J07Rik	RIKEN cDNA 9030607J07 gene	-0.80	4.21	-0.68	-5.48	0.00217	-0.93	-7.51	1.07E-04
1425442_at	Oscar	osteoclast associated receptor	-0.80	3.88	-0.82	-5.60	1.83E-03	-0.79	-5.44	1.69E-03
1456629_at	Kank3	KN motif and ankyrin repeat domains 3	-0.80	3.46	-0.81	-6.78	0.000391	-0.79	-6.59	3.36E-04
1452155_a_at	Ddx17	DEAD (Asp-Glu-Ala-Asp) box polypeptide 17	-0.80	9.55	-0.62	-5.54	0.00202	-0.98	-8.69	2.48E-05
1443235_at	Eif2ak4	eukaryotic translation initiation factor 2 alpha kinase 4	-0.80	3.14	-0.53	-4.42	1.00E-02	-1.06	-8.77	2.27E-05
1456313_x_at	Mrpl28	mitochondrial ribosomal protein L28	-0.79	4.60	-0.52	-5.49	0.00214	-1.07	-11.28	2.54E-06
1450061_at	Enc1	ectodermal-neural cortex 1	-0.79	10.26	-0.76	-5.40	0.002447	-0.82	-5.80	1.01E-03
1440826_s_at	2610002I17Rik	RIKEN cDNA 2610002I17	-0.79	8.32	-0.79	-4.71	0.006507	-0.79	-4.72	4.91E-03
1444879_at	Unknown	Unknown	-0.79	2.87	-0.67	-6.80	0.000381	-0.91	-9.34	1.38E-05
1446219_at	D930015E06Rik	RIKEN cDNA D930015E06 gene	-0.79	4.20	-0.79	-5.37	2.58E-03	-0.79	-5.33	2.00E-03
1460272_at	Ubl4b	ubiquitin-like 4B	-0.79	3.56	-0.71	-4.91	0.004809	-0.87	-5.99	7.70E-04
1427424_at	Galnt6	polypeptide N-acetylgalactosaminyltransferase 6	-0.79	4.56	-0.80	-5.97	0.001171	-0.77	-5.72	0.001132
1445910_at	C79999	expressed sequence C79999	-0.78	3.72	-0.56	-5.27	2.94E-03	-1.00	-9.44	1.25E-05
1432631_at	Prrc1	proline-rich coiled-coil 1	-0.78	5.18	-0.74	-5.75	0.001543	-0.82	-6.43	4.28E-04
1439672_at	Synj1	synaptojanin 1	-0.78	2.73	-0.94	-7.21	0.000225	-0.62	-4.71	5.03E-03
1459956_at	dock10	dedicator of cytokinesis 10	-0.78	3.23	-0.95	-8.38	6.23E-05	-0.61	-5.42	1.74E-03

1442201_at	Unknown	Unknown	-0.78	4.29	-0.74	-8.09	8.64E-05	-0.81	-8.91	2.04E-05
1445886_at	Elk3	ELK3, member of ETS oncogene family	-0.77	4.80	-0.92	-8.59	5.11E-05	-0.63	-5.95	8.15E-04
1445908_at	Eif2b5	Eukaryotic translation initiation factor 2B, subunit 5 epsilon	-0.77	3.60	-0.97	-9.07	3.27E-05	-0.58	-5.37	0.00185
1429863_at	Lonrf3	LON peptidase N-terminal domain and ring finger 3	-0.77	8.84	-0.64	-5.09	0.003754	-0.90	-7.15	0.000164
1431629_at	9130221D24Rik	RIKEN cDNA 9130221D24 gene	-0.77	4.87	-0.94	-8.14	8.26E-05	-0.60	-5.18	2.48E-03
1445540_at	Dnm3	dynamin 3	-0.77	3.74	-0.78	-6.00	0.001132	-0.76	-5.87	9.15E-04
1432743_at	4930443G03Rik	RIKEN cDNA 4930443G03 gene	-0.77	3.80	-0.68	-4.82	0.005527	-0.86	-6.04	7.26E-04
1438429_at	2610319H10Rik	RIKEN cDNA 2610319H10 gene	-0.77	2.86	-0.99	-8.39	6.23E-05	-0.55	-4.65	5.48E-03
1434525_at	Pkn3	protein kinase N3	-0.77	6.65	-0.74	-4.92	0.004785	-0.79	-5.26	2.20E-03
1458333_x_at	Utf1	undifferentiated embryonic cell transcription factor 1	-0.77	3.31	-0.63	-4.83	5.46E-03	-0.90	-6.93	0.000215
1444696_at	BC059841	CDNA sequence BC059841	-0.77	6.19	-0.77	-4.46	0.009436	-0.76	-4.42	0.007659
1453424_at	Fyco1	FYVE and coiled-coil domain containing 1	-0.76	4.28	-0.68	-5.78	0.001488	-0.85	-7.24	1.49E-04
1423464_at	D2Ertd750e	DNA segment, Chr 2, ERATO Doi 750, expressed	-0.76	5.12	-0.70	-6.37	6.88E-04	-0.83	-7.55	1.01E-04
1442681_at	Tsen2	tRNA splicing endonuclease 2 homolog (S. cerevisiae)	-0.76	5.24	-0.62	-4.67	0.006956	-0.91	-6.82	2.43E-04
1449819_at	Dmc1	DMC1 dosage suppressor of mck1 homolog	-0.76	4.00	-0.78	-5.48	0.002174	-0.74	-5.22	2.36E-03
1439136_at	Ssbp3	single-stranded DNA binding protein 3	-0.76	3.71	-0.91	-7.44	0.000171	-0.62	-5.06	2.97E-03
1433597_at	Arglu1	arginine and glutamate rich 1	-0.76	10.15	-0.71	-6.09	0.000998	-0.81	-6.89	0.000227
1444868_at	Lrrc9	Leucine rich repeat containing 9	-0.76	4.38	-0.77	-5.27	2.94E-03	-0.75	-5.13	0.002685
1442421_at	RIKEN...	RIKEN cDNA 2900083I11 gene	-0.76	4.99	-0.82	-6.10	0.000998	-0.70	-5.20	2.43E-03
1450631_x_at	Defa24	defensin, alpha, 24	-0.76	4.71	-0.67	-5.40	0.002431	-0.84	-6.72	2.82E-04
1459549_at	Dph5	DPH5 homolog (S. cerevisiae)	-0.76	5.07	-0.82	-5.78	1.49E-03	-0.70	-4.92	3.62E-03
1439310_at	A630012P03Rik	RIKEN cDNA A630012P03 gene	-0.75	3.99	-0.62	-6.31	0.000757	-0.88	-8.93	2.04E-05
1421253_at	Nrap	nebulin-related anchoring protein	-0.75	6.38	-0.50	-4.90	0.004903	-1.00	-9.70	9.01E-06
1427433_s_at	Hoxa3	homeobox A3	-0.75	3.23	-0.84	-7.06	0.00027	-0.66	-5.57	0.00142
1425108_a_at	Smagp	small cell adhesion glycoprotein	-0.75	5.15	-0.72	-4.45	0.009518	-0.78	-4.80	0.004345
1437603_at	BC050777	cDNA sequence BC050777	-0.75	3.61	-0.52	-5.86	1.36E-03	-0.98	-11.15	2.67E-06
1454906_at	Rarb	retinoic acid receptor, beta	-0.75	6.29	-0.67	-4.72	0.006449	-0.83	-5.84	9.59E-04
1445406_at	C77494	expressed sequence C77494	-0.75	3.81	-0.89	-6.38	0.000682	-0.60	-4.30	9.11E-03
1440309_at	RIKEN...	RIKEN cDNA 4833430A08 gene	-0.75	3.77	-0.57	-4.93	4.71E-03	-0.92	-7.91	6.54E-05
1439731_at	E130309F12Rik	RIKEN cDNA E130309F12 gene	-0.75	4.85	-0.92	-6.85	0.000356	-0.57	-4.27	9.60E-03
1423307_s_at	Tgoln1	trans-golgi network protein	-0.75	9.44	-0.82	-6.77	0.000391	-0.67	-5.53	0.001496
1430086_at	Chrna9	cholinergic receptor, nicotinic, alpha polypeptide 9	-0.74	3.42	-0.82	-8.40	6.23E-05	-0.67	-6.87	0.000233
1426608_at	Gm7120	predicted gene 7120	-0.74	3.28	-0.86	-6.89	0.000342	-0.62	-4.98	3.34E-03
1446908_at	Unknown	Unknown	-0.74	4.24	-0.70	-5.70	1.65E-03	-0.78	-6.37	4.68E-04
1441454_at	Zhx3	zinc fingers and homeoboxes 3	-0.74	10.76	-0.62	-6.54	0.000535	-0.86	-9.01	1.86E-05
1443730_at	Fam129b	family with sequence similarity 129, member B	-0.74	4.61	-0.67	-4.53	0.008441	-0.81	-5.49	1.58E-03
1429989_at	1700008A04Rik	RIKEN cDNA 1700008A04 gene	-0.74	5.08	-0.58	-4.68	6.88E-03	-0.89	-7.17	1.62E-04
1460421_at	Zfp133	zinc finger protein 133	-0.74	3.82	-0.75	-4.74	0.006236	-0.73	-4.59	0.005878
1431186_at	Dlg5	discs, large homolog 5 (Drosophila)	-0.74	4.65	-0.70	-4.51	0.00876	-0.77	-4.95	0.003485
1421187_at	Ccr2	chemokine (C-C motif) receptor 2	-0.74	2.88	-0.85	-6.77	0.000391	-0.63	-4.99	3.29E-03
1431536_at	4933432K03Rik	RIKEN cDNA 4933432K03 gene	-0.74	3.45	-0.61	-4.65	0.00717	-0.86	-6.54	3.58E-04
1419563_at	Birc6	baculoviral IAP repeat-containing 6	-0.73	5.80	-0.78	-7.12	2.48E-04	-0.69	-6.35	4.77E-04
1417542_at	Rps6ka2	ribosomal protein S6 kinase, polypeptide 2	-0.73	8.15	-0.77	-5.27	0.002942	-0.70	-4.84	4.10E-03
1446999_at	Unknown	Unknown	-0.73	4.05	-0.64	-4.63	0.007412	-0.83	-5.98	7.75E-04
1427084_a_at	Map4k5	mitogen-activated protein kinase kinase kinase kinase 5	-0.73	8.50	-0.71	-4.53	8.56E-03	-0.75	-4.78	0.004505
1417679_at	Gfi1	growth factor independent 1	-0.73	3.26	-0.72	-6.55	0.000527	-0.74	-6.76	0.000262
1441397_at	Pard3	par-3 (partitioning defective 3) homolog (C. elegans)	-0.73	3.66	-0.70	-6.28	0.000781	-0.76	-6.88	2.31E-04
1453945_at	2310005E17Rik	RIKEN cDNA 2310005E17 gene	-0.73	3.39	-0.69	-5.53	0.002033	-0.77	-6.21	5.70E-04
1458407_s_at	Unknown	Unknown	-0.73	6.07	-0.74	-5.16	0.003438	-0.71	-4.98	3.34E-03
1444527_at	Unknown	Unknown	-0.72	3.50	-0.59	-5.04	4.00E-03	-0.86	-7.35	1.30E-04
1443226_at	Sike1	Suppressor of IKBKE 1	-0.72	2.83	-0.69	-6.47	0.000597	-0.76	-7.16	1.64E-04
1430408_at	Cacna1a	calcium channel, voltage-dependent, P/Q type, alpha 1A subunit	-0.72	5.36	-0.57	-5.09	0.0038	-0.88	-7.91	6.54E-05
1416812_at	Tia1	cytotoxic granule-associated RNA binding protein 1	-0.72	7.89	-0.66	-5.65	1.74E-03	-0.79	-6.70	0.000284
1449114_at	Stk3	serine/threonine kinase 3 (Ste20, yeast homolog)	-0.72	4.34	-0.69	-5.09	0.0038	-0.75	-5.50	1.56E-03
1419815_at	Mett11d1	methyltransferase 11 domain containing 1	-0.72	5.90	-0.57	-4.80	0.005704	-0.87	-7.37	1.29E-04
1447383_at	Zfp810	zinc finger protein 810	-0.72	4.19	-0.52	-5.71	0.001631	-0.92	-10.15	6.82E-06
1433415_at	Rfx2	regulatory factor X, 2 (influences HLA class II expression)	-0.72	4.87	-0.67	-5.23	0.003078	-0.77	-6.03	7.27E-04
1416067_at	Ifrd1	interferon-related developmental regulator 1	-0.72	11.41	-0.63	-4.56	8.15E-03	-0.80	-5.77	1.05E-03
1447964_at	Ttl	tubulin tyrosine ligase	-0.71	5.11	-0.66	-5.14	0.003534	-0.76	-5.93	0.000832
1451711_at	Wnt9b	wingless-type MMTV integration site 9B	-0.71	5.23	-0.82	-6.91	0.000325	-0.60	-5.05	0.00301
1416916_at	Elf3	E74-like factor 3	-0.71	5.07	-0.64	-5.71	1.62E-03	-0.78	-6.91	2.23E-04
1425215_at	Ffar2	free fatty acid receptor 2	-0.71	4.43	-0.75	-7.03	0.000283	-0.67	-6.21	5.70E-04
1433930_at	Hpse	heparanase	-0.70	3.18	-0.73	-5.64	0.001747	-0.68	-5.22	2.33E-03

1433052_at	Elavl2	ELAV (embryonic lethal, abnormal vision, Drosophila)-like 2 (Hu antigen B)	-0.70	2.70	-0.60	-5.49	0.002159	-0.80	-7.30	1.38E-04
1458898_at	Unknown	Unknown	-0.70	3.80	-0.70	-5.61	0.001823	-0.71	-5.71	1.14E-03
1449182_at	Retn	resistin	-0.70	6.21	-0.61	-4.65	7.17E-03	-0.79	-5.99	0.00077
1442592_at	Ppapdc3	phosphatidic acid phosphatase type 2 domain containing 3	-0.70	5.50	-0.59	-4.71	0.006507	-0.81	-6.44	0.000418
1427503_at	AI324046	expressed sequence AI324046	-0.70	5.54	-0.70	-5.24	0.003039	-0.70	-5.19	2.47E-03
1441247_at	Innpp5j	inositol polyphosphate 5-phosphatase J	-0.70	3.41	-0.75	-5.27	2.94E-03	-0.65	-4.61	5.72E-03
1458495_at	Unknown	Unknown	-0.70	4.20	-0.93	-9.54	2.32E-05	-0.47	-4.80	4.38E-03
1434557_at	Hip1	huntingtin interacting protein 1	-0.70	8.54	-0.67	-5.26	0.002961	-0.72	-5.71	1.14E-03
1440943_at	B230208H17Rik	RIKEN cDNA B230208H17 gene	-0.69	3.99	-0.61	-5.66	0.001724	-0.78	-7.16	1.63E-04
1427454_at	Hoxc6	homeobox C6	-0.69	5.46	-0.79	-8.37	6.23E-05	-0.60	-6.34	0.000478
1443491_at	Ptprk	protein tyrosine phosphatase, receptor type, K	-0.69	3.15	-0.91	-9.20	2.93E-05	-0.47	-4.73	0.004837
1421406_at	Entpd7	ectonucleoside triphosphate diphosphohydrolase 7	-0.69	3.83	-0.64	-5.02	0.004158	-0.74	-5.79	1.03E-03
1439318_at	Mast4	microtubule associated serine/threonine kinase family member 4	-0.69	3.47	-0.63	-6.00	0.001126	-0.75	-7.08	1.81E-04
1447217_at	Uhrf2	ubiquitin-like, containing PHD and RING finger domains 2	-0.69	5.91	-0.71	-4.97	4.48E-03	-0.67	-4.72	4.93E-03
1445705_x_at	Dpp8	dipeptidylpeptidase 8	-0.69	5.40	-0.69	-5.35	0.002634	-0.69	-5.34	1.97E-03
1432281_a_at	Itgb6	integrin beta 6	-0.69	2.94	-0.95	-9.99	1.42E-05	-0.42	-4.43	7.49E-03
1441199_at	Ptprm	protein tyrosine phosphatase, receptor type, M	-0.69	4.33	-0.80	-6.51	0.000558	-0.57	-4.59	0.005869
1431367_at	March1	membrane-associated ring finger (C3HC4) 1	-0.69	4.17	-0.67	-4.96	0.0045	-0.70	-5.14	0.002647
1458052_at	Unknown	Unknown	-0.68	5.01	-0.82	-7.52	1.58E-04	-0.55	-5.06	2.98E-03
1441432_at	C820005J03Rik	RIKEN cDNA C820005J03 gene	-0.68	3.72	-0.47	-4.64	0.007345	-0.89	-8.79	2.24E-05
1456635_at	Gm2397	predicted gene 2397	-0.68	5.54	-0.58	-5.46	0.002252	-0.79	-7.39	1.27E-04
1447951_at	RIKEN…	RIKEN cDNA 5730403B10 gene	-0.68	5.31	-0.78	-6.61	4.88E-04	-0.58	-4.96	3.41E-03
1450256_at	Cer1	cerberus 1 homolog (Xenopus laevis)	-0.68	4.08	-0.62	-4.58	0.008037	-0.74	-5.49	1.58E-03
1448409_at	Lrmp	lymphoid-restricted membrane protein	-0.68	4.52	-0.68	-5.48	0.002174	-0.68	-5.43	0.001717
1421627_at	Evx1	even skipped homeotic gene 1 homolog	-0.68	4.53	-0.69	-5.84	0.001393	-0.66	-5.56	0.001443
1443592_at	AA617406	expressed sequence AA617406	-0.68	4.50	-0.72	-6.13	0.00096	-0.63	-5.36	1.89E-03
1424295_at	Dppa3	developmental pluripotency-associated 3	-0.68	5.14	-0.83	-6.92	3.23E-04	-0.52	-4.38	8.16E-03
1440828_x_at	Phf7	PHD finger protein 7	-0.68	3.29	-0.75	-6.25	0.000814	-0.60	-4.96	3.42E-03
1456832_at	Atrx	alpha thalassemia/mental retardation syndrome X-linked homolog (human)	-0.67	4.39	-0.59	-4.78	0.005911	-0.76	-6.16	6.13E-04
1437091_at	Accn4	amiloride-sensitive cation channel 4, pituitary	-0.67	5.31	-0.66	-6.27	7.88E-04	-0.69	-6.51	3.77E-04
1460029_at	Gtpbp5	GTP binding protein 5	-0.67	5.66	-0.72	-7.30	0.0002	-0.62	-6.32	0.000489
1421113_at	Pga5	pepsinogen 5, group I	-0.67	4.03	-0.57	-4.96	0.004526	-0.78	-6.77	0.000262
1459244_at	AU014756	expressed sequence AU014756	-0.67	3.01	-0.58	-5.87	0.001355	-0.76	-7.65	8.88E-05
1442446_at	Zfp523	Zinc finger protein 523	-0.67	5.21	-0.51	-4.78	0.00591	-0.83	-7.81	7.20E-05
1419762_at	Ubd	ubiquitin D	-0.67	4.53	-0.73	-6.18	8.97E-04	-0.61	-5.11	2.77E-03
1442085_at	Whsc1l1	Wolf-Hirschhorn syndrome candidate 1-like 1 (human)	-0.67	6.14	-0.71	-5.92	0.001257	-0.63	-5.28	2.15E-03
1437954_at	RIKEN…	RIKEN cDNA 2410016O06 gene	-0.67	5.18	-0.62	-4.69	0.006779	-0.72	-5.48	1.61E-03
1458125_at	Unknown	Unknown	-0.67	3.80	-0.55	-4.96	4.50E-03	-0.78	-6.98	0.000207
1443519_at	Gm11203	predicted gene 11203	-0.67	3.30	-0.80	-6.97	0.000302	-0.53	-4.64	0.005558
1449755_at	D7Ertd183e	DNA segment, Chr 7, ERATO Doi 183, expressed	-0.66	4.31	-0.58	-5.14	0.003523	-0.75	-6.67	2.99E-04
1445753_at	Aox3l1	aldehyde oxidase 3-like 1	-0.66	3.13	-0.66	-6.69	0.00044	-0.67	-6.84	2.40E-04
1433050_at	5730478J17Rik	RIKEN cDNA 5730478J17 gene	-0.66	4.00	-0.65	-5.40	0.002431	-0.68	-5.64	1.29E-03
1434923_at	Cox19	COX19 cytochrome c oxidase assembly homolog (S. cerevisiae)	-0.66	9.22	-0.69	-5.42	2.38E-03	-0.63	-4.94	3.52E-03
1451739_at	Klf5	Kruppel-like factor 5	-0.66	3.29	-0.81	-6.93	0.000322	-0.51	-4.35	8.51E-03
1434803_a_at	Sycn	syncollin	-0.66	5.93	-0.63	-5.67	0.001709	-0.69	-6.23	0.000552
1419621_at	Ankrd2	ankyrin repeat domain 2 (stretch responsive muscle)	-0.66	5.70	-0.68	-5.16	3.41E-03	-0.63	-4.77	0.004511
1432793_at	4930401B11Rik	RIKEN cDNA 4930401B11 gene	-0.66	3.56	-0.72	-6.41	0.000649	-0.59	-5.23	2.32E-03
1421725_at	Slc26a5	solute carrier family 26, member 5	-0.65	4.81	-0.81	-7.11	0.000253	-0.49	-4.31	9.10E-03
1446003_at	Unknown	Unknown	-0.65	4.50	-0.61	-5.08	0.0038	-0.70	-5.84	9.58E-04
1443625_at	Naa40	N(alpha)-acetyltransferase 40, NatD catalytic subunit, homolog	-0.65	3.53	-0.68	-5.54	0.002023	-0.63	-5.11	2.78E-03
1451712_at	8030411F24Rik	RIKEN cDNA 8030411F24 gene	-0.65	3.67	-0.55	-5.21	3.21E-03	-0.75	-7.06	1.86E-04
1452019_at	Cyyr1	cysteine and tyrosine-rich protein 1	-0.65	4.90	-0.63	-4.59	0.007875	-0.67	-4.92	0.003625
1425883_at	Smg6	Smg-6 homolog, nonsense mediated mRNA decay factor (C. elegans)	-0.65	3.45	-0.61	-4.81	0.005629	-0.69	-5.49	0.001577
1418475_at	Scnn1b	sodium channel, nonvoltage-gated 1 beta	-0.65	4.69	-0.66	-6.08	1.01E-03	-0.64	-5.83	9.63E-04
1429960_at	4930438A08Rik	RIKEN cDNA 4930438A08 gene	-0.65	4.87	-0.79	-8.45	6.05E-05	-0.50	-5.36	1.89E-03
1444898_at	Unknown	Unknown	-0.65	4.54	-0.64	-5.13	0.003606	-0.65	-5.24	2.30E-03
1449495_at	Reg3a	regenerating islet-derived 3 alpha	-0.64	4.01	-0.57	-5.73	0.001585	-0.72	-7.25	1.47E-04
1448397_at	Gjb6	gap junction protein, beta 6	-0.64	11.27	-0.59	-5.79	0.001486	-0.70	-6.79	2.56E-04
1445865_at	Unknown	Unknown	-0.64	3.31	-0.75	-6.07	1.02E-03	-0.53	-4.28	0.00941
1444237_at	LOC100045758	hypothetical protein LOC100045758; similar to EMO2	-0.64	4.75	-0.55	-5.30	0.002813	-0.73	-6.97	0.000207
1452349_x_at	Ifi205	interferon activated gene 205	-0.64	2.63	-0.71	-5.76	0.001518	-0.57	-4.58	6.00E-03
1419654_at	Tle3	transducin-like enhancer of split 3, homolog of Drosophila E(spl)	-0.64	9.00	-0.58	-4.66	7.03E-03	-0.69	-5.52	1.53E-03
1430922_at	5530401A14Rik	RIKEN cDNA 5530401A14 gene	-0.63	3.03	-0.68	-5.53	0.002027	-0.58	-4.74	4.77E-03

1446930_at	Unknown	Unknown	-0.63	2.82	-0.52	-4.64	0.007282	-0.75	-6.71	2.83E-04
1438163_x_at	Rhbdd2	rhomboid domain containing 2	-0.63	4.31	-0.53	-4.93	0.004708	-0.73	-6.76	2.62E-04
1442392_at	C1qtnf7	C1q and tumor necrosis factor related protein 7	-0.63	3.34	-0.64	-5.20	0.003228	-0.62	-5.04	0.003073
1433191_at	A430105P17Rik	RIKEN cDNA A430105P17 gene	-0.63	3.00	-0.67	-4.92	4.77E-03	-0.59	-4.36	0.008373
1444109_at	C130009A20Rik	RIKEN cDNA C130009A20 gene	-0.63	3.38	-0.66	-4.80	0.005744	-0.60	-4.34	8.61E-03
1437452_x_at	Vdac1	voltage-dependent anion channel 1	-0.63	5.99	-0.52	-5.53	0.002033	-0.73	-7.76	7.72E-05
1441086_at	4930451I11Rik	RIKEN cDNA 4930451I11 gene	-0.62	5.34	-0.62	-4.73	6.34E-03	-0.63	-4.81	4.27E-03
1422869_at	Mertk	c-mer proto-oncogene tyrosine kinase	-0.62	10.64	-0.61	-4.85	0.00523	-0.63	-5.02	3.15E-03
1442699_at	ENSMUSG…	ENSMUSG00000084048	-0.62	5.12	-0.57	-5.29	0.002859	-0.67	-6.29	5.14E-04
1440214_at	RIKEN…	RIKEN cDNA A630001G21 gene	-0.62	3.58	-0.60	-4.54	0.008372	-0.64	-4.86	0.003935
1442197_at	AI480624	expressed sequence AI480624	-0.62	4.72	-0.66	-5.80	0.001459	-0.58	-5.10	0.002809
1428002_at	Saal1	serum amyloid A-like 1	-0.62	3.52	-0.57	-5.65	1.74E-03	-0.66	-6.50	3.83E-04
1432308_at	1700058C13Rik	RIKEN cDNA 1700058C13 gene	-0.61	3.24	-0.76	-7.31	0.0002	-0.46	-4.45	7.35E-03
1456510_x_at	Higd1c	HIG1 domain family, member 1C	-0.61	8.38	-0.74	-7.25	0.000215	-0.49	-4.76	4.60E-03
1428634_at	Twistnb	TWIST neighbor	-0.61	8.23	-0.61	-4.68	6.83E-03	-0.61	-4.73	4.81E-03
1422274_at	Gja8	gap junction protein, alpha 8	-0.61	5.43	-0.55	-5.72	0.001596	-0.67	-6.95	2.10E-04
1433396_at	4921504P13Rik	RIKEN cDNA 4921504P13 gene	-0.61	4.55	-0.57	-4.60	0.007784	-0.65	-5.22	0.002341
1448793_a_at	Sdc4	syndecan 4	-0.61	12.04	-0.59	-5.83	0.001415	-0.62	-6.18	0.000589
1431305_at	Mcoln1	mucolipin 1	-0.60	5.92	-0.61	-5.06	0.003896	-0.60	-5.00	3.27E-03
1429565_s_at	Lce1m	late cornified envelope 1M	-0.60	5.51	-0.65	-5.44	2.30E-03	-0.55	-4.62	5.71E-03
1445222_at	Cd22	CD22 antigen; hypothetical protein LOC100047973	-0.60	4.07	-0.52	-4.51	0.008762	-0.68	-5.82	9.79E-04
1454321_at	1500005C15Rik	RIKEN cDNA 1500005C15 gene	-0.60	4.32	-0.59	-4.70	0.006664	-0.61	-4.89	3.80E-03
1416436_a_at	Uqcc	ubiquinol-cytochrome c reductase complex chaperone, CBP3 homolog	-0.60	8.66	-0.56	-4.93	4.71E-03	-0.64	-5.66	1.24E-03
1443115_at	Tgfbr2	transforming growth factor, beta receptor II	-0.60	3.69	-0.55	-4.44	0.009699	-0.65	-5.22	0.002341
1459420_at	Unknown	Unknown	-0.60	5.06	-0.57	-4.74	0.006238	-0.62	-5.14	0.002647
1432041_at	4930519A11Rik	RIKEN cDNA 4930519A11 gene	-0.59	2.96	-0.48	-4.82	0.005555	-0.70	-6.95	2.10E-04
1442323_at	Slc10a7	solute carrier family 10 (sodium/bile acid cotransporter family), member 7	-0.59	3.05	-0.63	-4.87	0.005089	-0.55	-4.27	9.69E-03
1422700_at	Alox12	arachidonate 12-lipoxygenase	-0.59	4.13	-0.54	-5.66	1.73E-03	-0.64	-6.69	2.89E-04
1436977_at	Unknown	Unknown	-0.59	4.85	-0.60	-5.11	0.00365	-0.58	-5.01	3.23E-03
1458914_at	Eral1	Era (G-protein)-like 1 (E. coli)	-0.59	2.86	-0.61	-4.74	0.006295	-0.57	-4.47	7.09E-03
1456619_at	Liph	lipase, member H	-0.59	3.50	-0.62	-5.70	1.65E-03	-0.56	-5.11	0.002779
1431941_at	Slc35b4	solute carrier family 35, member B4	-0.58	5.10	-0.66	-5.78	0.001499	-0.51	-4.53	0.006431
1445999_at	D19Ertd132e	DNA segment, Chr 19, ERATO Doi 132, expressed	-0.58	3.26	-0.62	-5.88	0.001328	-0.54	-5.15	2.61E-03
1446564_at	D18Ertd169e	DNA segment, Chr 18, ERATO Doi 169, expressed	-0.58	3.38	-0.43	-4.89	0.004931	-0.73	-8.26	4.16E-05
1450176_at	Ern1	endoplasmic reticulum (ER) to nucleus signalling 1	-0.57	4.77	-0.55	-5.06	0.003896	-0.59	-5.42	1.74E-03
1441812_at	Cyp2u1	cytochrome P450, family 2, subfamily u, polypeptide 1	-0.57	4.15	-0.62	-6.46	6.05E-04	-0.52	-5.46	1.66E-03
1432275_at	Ikbkb	inhibitor of kappaB kinase beta	-0.57	4.20	-0.65	-5.82	0.00143	-0.49	-4.45	7.35E-03
1441414_at	Sfi1	Sfi1 homolog, spindle assembly associated (yeast)	-0.57	5.32	-0.54	-4.71	0.006507	-0.60	-5.20	0.002421
1447045_at	Unknown	Unknown	-0.57	2.39	-0.62	-5.82	1.42E-03	-0.51	-4.81	0.004272
1447328_at	Unknown	Unknown	-0.56	4.66	-0.51	-4.60	0.00786	-0.62	-5.61	1.33E-03
1456920_at	Unknown	Unknown	-0.56	4.48	-0.55	-5.48	0.002171	-0.58	-5.75	1.09E-03
1427771_x_at	Itgb1	integrin beta 1 (fibronectin receptor beta)	-0.56	4.16	-0.55	-4.44	0.009699	-0.57	-4.60	5.82E-03
1431567_at	1700018A04Rik	RIKEN cDNA 1700018A04 gene	-0.56	2.95	-0.50	-4.48	0.009099	-0.62	-5.50	1.56E-03
1435782_at	Gm12617	Uba52 pseudogene	-0.56	7.07	-0.57	-5.08	3.80E-03	-0.55	-4.97	3.39E-03
1420152_at	Unknown	Unknown	-0.56	3.20	-0.61	-5.25	0.002971	-0.50	-4.31	0.009102
1419699_at	Scgb3a1	secretoglobin, family 3A, member 1	-0.56	4.80	-0.61	-5.75	0.001543	-0.50	-4.74	0.004803
1440078_at	Brd4	bromodomain containing 4	-0.56	3.54	-0.53	-4.79	5.78E-03	-0.59	-5.33	1.98E-03
1425678_a_at	Snrk	SNF related kinase	-0.55	8.79	-0.54	-4.50	0.008946	-0.56	-4.67	5.30E-03
1445403_at	Unknown	Unknown	-0.54	3.87	-0.56	-5.02	0.004158	-0.53	-4.78	4.50E-03
1435736_x_at	Ift27	intraflagellar transport 27 homolog (Chlamydomonas)	-0.54	10.53	-0.54	-4.71	0.006507	-0.55	-4.83	4.13E-03
1425915_at	Slc26a8	solute carrier family 26, member 8	-0.54	5.78	-0.54	-4.62	0.007493	-0.54	-4.62	5.69E-03
1432253_at	4921523P09Rik	RIKEN cDNA 4921523P09 gene	-0.54	2.81	-0.51	-4.74	6.29E-03	-0.57	-5.21	0.002382
1446184_at	Unknown	Unknown	-0.54	3.07	-0.51	-4.42	0.009965	-0.57	-4.89	0.003826
1442776_at	Unknown	Unknown	-0.54	4.89	-0.51	-4.58	0.008044	-0.56	-4.95	3.49E-03
1446807_at	Usp8	ubiquitin specific peptidase 8	-0.54	3.85	-0.50	-4.49	8.98E-03	-0.58	-5.22	2.34E-03
1445006_at	Unknown	Unknown	-0.53	3.31	-0.61	-6.12	0.000969	-0.45	-4.46	7.13E-03
1454117_at	5330439B14Rik	RIKEN cDNA 5330439B14 gene	-0.53	3.75	-0.60	-5.82	0.00142	-0.46	-4.53	6.41E-03
1421247_at	Pax9	paired box gene 9	-0.53	5.69	-0.53	-4.48	0.009087	-0.52	-4.38	8.08E-03
1444442_at	Oraov1	oral cancer overexpressed 1	-0.52	4.67	-0.48	-4.49	0.009019	-0.56	-5.32	0.002036
1426003_at	Ntrk3	neurotrophic tyrosine kinase, receptor, type 3	-0.50	4.15	-0.47	-5.46	2.22E-03	-0.53	-6.21	0.000567
1432551_at	1700031F10Rik	RIKEN cDNA 1700031F10 gene	-0.49	5.12	-0.48	-4.59	0.00786	-0.50	-4.75	4.67E-03
1444912_at	Unknown	Unknown	-0.48	4.95	-0.48	-4.57	0.008115	-0.48	-4.60	5.85E-03
1447273_x_at	Atp10a	ATPase, class V, type 10A	-0.48	3.17	-0.50	-4.78	5.88E-03	-0.46	-4.40	7.88E-03

1445928_at	March6	membrane-associated ring finger (C3HC4) 6	-0.48	4.80	-0.49	-4.89	0.004926	-0.47	-4.64	5.57E-03
1443056_at	RIKEN…	RIKEN cDNA A230006I23 gene	-0.47	2.56	-0.48	-4.92	0.004772	-0.45	-4.62	5.69E-03
1433691_at	Ppp1r3c	protein phosphatase 1, regulatory (inhibitor) subunit 3C	-0.47	11.42	-0.46	-5.48	0.002172	-0.48	-5.69	0.001174
1417789_at	Ccl11	chemokine (C-C motif) ligand 11	-0.47	3.55	-0.46	-4.62	0.007604	-0.47	-4.72	0.00494
1433010_at	5530400N10Rik	RIKEN cDNA 5530400N10 gene	-0.45	3.58	-0.48	-4.92	4.77E-03	-0.42	-4.30	9.11E-03
1449332_at	Fhl5	four and a half LIM domains 5	-0.43	4.29	-0.45	-5.38	0.002509	-0.42	-5.07	2.93E-03
1428958_at	Paqr8	progestin and adipoQ receptor family member VIII	-0.43	11.17	-0.41	-4.74	0.006295	-0.44	-5.15	2.61E-03
1440580_at	Efr3b	EFR3 homolog B (S. cerevisiae)	-0.42	5.15	-0.41	-4.64	7.30E-03	-0.42	-4.75	4.72E-03
