# Supplementary material for: Effects of sleep and wake on astrocytes: clues from molecular and ultrastructural studies
Source: BMC Biol. 2015 Aug 25;13:66. doi: 10.1186/s12915-015-0176-7 (PMC4548305; doi:10.1186/s12915-015-0176-7)
Supplement: Additional file 5: Table S5. — Functional clusters for sleep genes. (PDF 188 kb) [file 12915_2015_176_MOESM5_ESM.pdf]

Table 5. Functional clusters for sleep genes

GO:0032502~ development, developmental process		GO:0005856~ cytoskeleton		GO:0044249~ cellular biosynthetic process		GO:0003700~ transcription factor activity	
1426230_AT	sphk2	1442361_AT	park2	1438211_S_AT	dbp	1438211_S_AT	dbp
1439193_AT	prrxl1	1434588_X_AT	tbca	1426230_AT	sphk2	1439193_AT	prrxl1
1429905_AT	lhx9	1419833_S_AT	arap3	1448470_AT	fbp1	1429905_AT	lhx9
1448975_S_AT	ren1	1450496_A_AT	ska1	1439193_AT	prrxl1	1420389_AT	pax3
1420389_AT	pax3	1415779_S_AT	actg1	1446617_AT	st8sia4	1418174_AT	dbp
1427018_AT	tsnaxip1	1449178_AT	pdlim3	1420389_AT	pax3	1424233_AT	meox2
1415779_S_AT	actg1			1418174_AT	dbp	1444596_AT	pax7
1449178_AT	pdlim3			1432170_AT	tsfm		
1424233_AT	meox2			1428604_AT	2610305d13rik		
1419626_AT	adam25			1460490_AT	mrpl15		
1444596_AT	pax7			1457640_X_AT	pigs		
				1444596_AT	pax7		

GO:0043229~ intracellular organelle		GO:0043234~ protein complex		GO:0050794~ regulation of cellular process	
1442361_AT	park2	1442361_AT	park2	1442361_AT	park2
1425008_A_AT	ifi203	1434588_X_AT	tbca	1450810_AT	fshr
1438211_S_AT	dbp	1450496_A_AT	ska1	1427327_AT	pilra
1417700_AT	rab38	1420389_AT	pax3	1438211_S_AT	dbp
1450496_A_AT	ska1	1440054_AT	pik3ca	1417700_AT	rab38
1419833_S_AT	arap3	1415779_S_AT	actg1	1450494_X_AT	ceacam1
1446617_AT	st8sia4	1444596_AT	pax7	1423854_A_AT	rasl11b
1418174_AT	dbp			1450496_A_AT	ska1
1432170_AT	tsfm			1419833_S_AT	arap3
1449178_AT	pdlim3			1448975_S_AT	ren1
1457640_X_AT	pigs			1418174_AT	dbp
1415779_S_AT	actg1			1424233_AT	meox2
1460490_AT	mrpl15			1444596_AT	pax7
1424233_AT	meox2			1426230_AT	sphk2
1444596_AT	pax7			1439193_AT	prrxl1
1434588_X_AT	tbca			1429905_AT	lhx9
1439193_AT	prrxl1			1420389_AT	pax3
1429905_AT	lhx9			1440054_AT	pik3ca
1420389_AT	pax3			1428604_AT	2610305d13rik
1428604_AT	2610305d13rik				
1446274_AT	slc16a1				
1416332_AT	cirbp				
