# Supplementary material for: Effects of sleep and wake on astrocytes: clues from molecular and ultrastructural studies
Source: BMC Biol. 2015 Aug 25;13:66. doi: 10.1186/s12915-015-0176-7 (PMC4548305; doi:10.1186/s12915-015-0176-7)
Supplement: Additional file 6: Table S6. — Functional clusters for wake genes. (PDF 256 kb) [file 12915_2015_176_MOESM6_ESM.pdf]

Table 6. Functional clusters for wake genes

GO:0008152~metabolic process		GO:0017076~ purine nucleotide binding		GO:0048856~ anatomical structure development		GO:0031323~ regulation of cellular metabolic process	
1451739_AT	klf5	1418614_AT	kcnj1	1445908_AT	eif2b5	1445908_AT	eif2b5
1458387_AT	ptdss2	1419766_AT	sik1	1451739_AT	klf5	1451739_AT	klf5
1443625_AT	naa40	1447964_AT	ttl	1418687_AT	arc	1418099_AT	tnfrsf1b
1446807_AT	usp8	1438099_AT	trio	1427683_AT	egr2	1427683_AT	egr2
1427683_AT	egr2	1449114_AT	stk3	1447964_AT	ttl	1419410_AT	batf
1427433_S_AT	hoxa3	1449819_AT	dmc1	1427433_S_AT	hoxa3	1427433_S_AT	hoxa3
1418322_AT	crem	1429319_AT	rhoh	1449819_AT	dmc1	1418322_AT	crem
1421247_AT	pax9	1445540_AT	dnm3	1421247_AT	pax9	1419654_AT	tle3
1428634_AT	twistnb	1433584_AT	tuba1a	1429319_AT	rhoh	1416812_AT	tia1
1434557_AT	hip1	1420527_S_AT	tcp10	1435782_AT	rps27a	1421247_AT	pax9
1417542_AT	rps6ka2	1443235_AT	eif2ak4	1438542_AT	trp53	1443235_AT	eif2ak4
1457956_AT	camta1	1438542_AT	trp53	1444705_AT	app	1434557_AT	hip1
1457984_AT	crh	1417542_AT	rps6ka2	1419762_AT	ubd	1438542_AT	trp53
1430408_AT	cacna1a	1431718_AT	kif15	1457984_AT	crh	1457956_AT	camta1
1439613_AT	adcy10	1426063_A_AT	gem	1430408_AT	cacna1a	1444705_AT	app
1447273_X_AT	atp10a	1439613_AT	adcy10	1423100_AT	fos	1430408_AT	cacna1a
1426429_AT	thap7	1447273_X_AT	atp10a	1450256_AT	cer1	1423100_AT	fos
1416505_AT	nr4a1	1425678_A_AT	snrk	1448804_AT	cyp11a1	1441454_AT	zhx3
1425678_A_AT	snrk	1422869_AT	mertk	1417394_AT	klf4	1426429_AT	thap7
1445734_AT	tdh	1417431_A_AT	sphk2	1417431_A_AT	sphk2	1434618_AT	crebzf
1458361_AT	dclre1c	1450176_AT	ern1	1458361_AT	dclre1c	1440959_S_AT	mynn
1452349_X_AT	ifi205	1434525_AT	pkn3	1427771_X_AT	itgb1	1416505_AT	nr4a1
1437954_AT	2410016o06rik	1432623_AT	supv3l1	1417789_AT	ccl11	1456846_AT	zbtb42
1434525_AT	pkn3	1459956_AT	dock10	1440748_AT	scarf1	1458582_AT	lin54
1417950_A_AT	apoa2	1417214_AT	rab27b	1429777_AT	dnajb6	1417394_AT	klf4
1438308_AT	agbl4	1435736_X_AT	rabl4	1454906_AT	rarb	1417431_A_AT	sphk2
1449568_AT	klb	1452155_A_AT	ddx17	1435998_AT	ccnb1ip1	1437954_AT	2410016o06rik
1441812_AT	cyp2u1	1427126_AT	hspa1l	1418220_AT	foxf2	1452349_X_AT	ifi205
1452344_AT	synj2	1423026_AT	rad51c	1421187_AT	ccr2	1450176_AT	ern1
1425883_AT	smg6	1443115_AT	tgfbr2	1443115_AT	tgfbr2	1417950_A_AT	apoa2
1454906_AT	rarb	1459823_AT	ehd2	1435663_AT	esr1	1458333_X_AT	utf1
1418220_AT	foxf2	1427084_A_AT	map4k5	1441397_AT	pard3	1429777_AT	dnajb6
1423026_AT	rad51c	1427127_X_AT	hspa1l	1421080_AT	nr4a3	1454906_AT	rarb
1427126_AT	hspa1l	1445753_AT	aox3l1	1448397_AT	gjb6	1441439_AT	ucn3
1443115_AT	tgfbr2	1419981_AT	riok3	1445886_AT	elk3	1418220_AT	foxf2
1427127_X_AT	hspa1l	1427836_AT	0610007p08rik	1416067_AT	ifrd1	1443115_AT	tgfbr2
1419981_AT	riok3	1440943_AT	b230208h17rik	1427454_AT	hoxc6	1435663_AT	esr1
1441639_AT	zcchc8	1439930_AT	smarca2	1451711_AT	wnt9b	1445669_AT	spry4
1458363_AT	zdhhc17	1460212_AT	gnat1	1450288_AT	cdh6	1447383_AT	zfp810
1442085_AT	whsc1l1	1455721_AT	gspt2	1460212_AT	gnat1	1420991_AT	ankrd1
1427836_AT	0610007p08rik	1442911_AT	riok2	1419563_AT	birc6	1446684_AT	ralgapa1
1421080_AT	nr4a3	1432275_AT	ikbkb	1422274_AT	gja8	1442085_AT	whsc1l1
1418936_AT	maff	1439318_AT	mast4	1441247_AT	inpp5j	1421080_AT	nr4a3
1443202_AT	cyb5b	1445339_AT	cnga4	1446350_AT	itga4	1418936_AT	maff
1459549_AT	dph5	1426003_AT	ntrk3	1450749_A_AT	nr4a2	1445886_AT	elk3
1430819_AT	naaa	1422978_AT	cybb	1417679_AT	gfi1	1442446_AT	zfp523
1445886_AT	elk3	1447728_X_AT	hspa9	1421532_AT	rxfp2	1439136_AT	ssbp3
1448539_A_AT	acy3	1458914_AT	eral1	1426003_AT	ntrk3	1437882_S_AT	6330416l07rik
1456619_AT	liph	1456832_AT	atrx	1416916_AT	elf3	1415810_AT	uhrf1
1419024_AT	ptp4a1	1460029_AT	gtpbp5	1456832_AT	atrx	1427454_AT	hoxc6
1421406_AT	entpd7	1439386_X_AT	mat2a	1430086_AT	chrna9	1456313_X_AT	mrpl28
1437882_S_AT	6330416l07rik	1417924_AT	pak3	1421627_AT	evx1	1439930_AT	smarca2
1439136_AT	ssbp3					1416814_AT	tia1
1441199_AT	ptprm					1456771_AT	zer1
1415810_AT	uhrf1					1441247_AT	inpp5j
1419563_AT	birc6					1432275_AT	ikbkb

1442911_AT	riok2
1442681_AT	tsen2
1432275_AT	ikbkb
1422978_AT	cybb
1416916_AT	elf3
1436090_AT	enpp6
1456832_AT	atrx
1439386_X_AT	mat2a
1417924_AT	pak3
1445908_AT	eif2b5
1436654_AT	gen1
1419766_AT	sik1
1448830_AT	dusp1
1419410_AT	batf
1447964_AT	ttl
1438099_AT	trio
1419654_AT	tle3
1456510_X_AT	higd1c
1437326_X_AT	cela3b
1421113_AT	pga5
1449114_AT	stk3
1449819_AT	dmc1
1449760_AT	ddost
1443235_AT	eif2ak4
1420527_S_AT	tcp10
1452676_A_AT	pnpt1
1450061_AT	enc1
1445705_X_AT	dpp8
1449332_AT	fhl5
1435782_AT	rps27a
1445456_AT	kcmf1
1438542_AT	trp53
1444705_AT	app
1419762_AT	ubd
1439672_AT	synj1
1443491_AT	ptprk
1425918_AT	egln3
1441454_AT	zhx3
1431367_AT	march1
1448804_AT	cyp11a1
1434618_AT	crebzf
1440959_S_AT	mynn
1420964_AT	enc1
1433691_AT	ppp1r3c
1458582_AT	lin54
1422869_AT	mertk
1417431_A_AT	sphk2
1417394_AT	klf4
1450176_AT	ern1
1458333_X_AT	utf1
1444507_AT	usp53
1440078_AT	brd4
1427880_AT	uqcrq
1435663_AT	esr1
1427084_A_AT	map4k5
1447383_AT	zfp810
1445753_AT	aox3l1
1427345_A_AT	sult1a1
1445928_AT	march6
1456070_AT	ptprg
1442446_AT	zfp523
1427652_X_AT	synj2
1449685_S_AT	oxsm

1450749_A_AT	nr4a2
1417679_AT	gfi1
1421532_AT	rxfp2
1443525_AT	dbx2
1441742_AT	kcnh5
1416916_AT	elf3
1434741_AT	rreb1
1456832_AT	atrx
1453503_AT	spink12
1433415_AT	rfx2
1459979_X_AT	zfp68
1421627_AT	evx1

1450822_AT	lyz2
1427454_AT	hoxc6
1419815_AT	mett11d1
1429863_AT	lonrf3
1429437_AT	prpf40a
1456696_X_AT	rmi1
1455721_AT	gspt2
1456771_AT	zer1
1459337_AT	alg6
1439318_AT	mast4
1450749_A_AT	nr4a2
1417679_AT	gfi1
1447217_AT	uhrf2
1422700_AT	alox12
1426003_AT	ntrk3
1430314_AT	4933437f05rik
1447728_X_AT	hspa9
1434741_AT	rreb1
1433415_AT	rfx2
1459979_X_AT	zfp68
1432110_AT	4930402f06rik

GO:0006897~ endocytosis		GO:0016310~ phosphorylation		GO:0005525~ GTP binding		GO:0043167~ ion binding	
1418687_AT	arc	1419766_AT	sik1	1440943_AT	b230208h1[illegible]	1451739_AT	klf5
1434557_AT	hip1	1443115_AT	tgfbr2	1459823_AT	ehd2	1436654_AT	gen1
1443115_AT	tgfbr2	1438099_AT	trio	1460212_AT	gnat1	1418614_AT	kcnj1
1459823_AT	ehd2	1427084_A_AT	map4k5	1455721_AT	gspt2	1419766_AT	sik1
1444705_AT	app	1419981_AT	riok3	1429319_AT	rhoh	1427683_AT	egr2
1421314_AT	cttn	1449114_AT	stk3	1445540_AT	dnm3	1447964_AT	ttl
1445540_AT	dnm3	1425678_A_AT	snrk	1433584_AT	tuba1a	1449114_AT	stk3
1421044_AT	mrc2	1422869_AT	mertk	1459956_AT	dock10	1449332_AT	fhl5
1422869_AT	mertk	1443235_AT	eif2ak4	1417214_AT	rab27b	1442323_AT	slc10a7
1439672_AT	synj1	1420527_S_AT	tcp10	1435736_X_AT	rabl4	1435782_AT	rps27a
		1442911_AT	riok2	1458914_AT	eral1	1445456_AT	kcmf1
		1439318_AT	mast4	1460029_AT	gtpbp5	1438542_AT	trp53
		1432275_AT	ikbkb	1426063_A_AT	gem	1417542_AT	rps6ka2
		1450176_AT	ern1			1447668_X_AT	efemp2
		1434525_AT	pkn3			1427306_AT	ryr1
		1417542_AT	rps6ka2			1433930_AT	hpse
		1444705_AT	app			1444705_AT	app
		1426003_AT	ntrk3			1455261_AT	luc7l
		1440078_AT	brd4			1421253_AT	nrap
		1417924_AT	pak3			1430408_AT	cacna1a
						1439613_AT	adcy10
						1447273_X_AT	atp10a
						1425918_AT	egln3
						1440828_X_AT	phf7
						1441454_AT	zhx3
						1426429_AT	thap7
						1453424_AT	fyco1
						1431367_AT	march1
						1448804_AT	cyp11a1
						1416505_AT	nr4a1
						1440959_S_AT	mynn
						1456846_AT	zbtb42
						1431305_AT	mcoln1
						1425678_A_AT	snrk
						1447010_AT	zfp609
						1437091_AT	accn4
						1417394_AT	klf4

1450176_AT	ern1
1458361_AT	dclre1c
1438308_AT	agbl4
1441812_AT	cyp2u1
1449568_AT	klb
1425883_AT	smg6
1454906_AT	rarb
1450645_AT	mt4
1443115_AT	tgfbr2
1435663_AT	esr1
1459823_AT	ehd2
1447383_AT	zfp810
1445753_AT	aox3l1
1441639_AT	zcchc8
1445928_AT	march6
1458363_AT	zdhhc17
1442085_AT	whsc1l1
1421080_AT	nr4a3
1457842_AT	zfp292
1443202_AT	cyb5b
1418475_AT	scnn1b
1435560_AT	itgal
1442446_AT	zfp523
1448539_A_AT	acy3
1421406_AT	entpd7
1437882_S_AT	6330416l07rik
1415810_AT	uhrf1
1419815_AT	mett11d1
1450288_AT	cdh6
1429863_AT	lonrf3
1439318_AT	mast4
1446350_AT	itga4
1450749_A_AT	nr4a2
1417679_AT	gfi1
1447217_AT	uhrf2
1422700_AT	alox12
1432840_AT	galnt11
1441742_AT	kcnh5
1422978_AT	cybb
1434741_AT	rreb1
1456832_AT	atrx
1459979_X_AT	zfp68
1421044_AT	mrc2
1439386_X_AT	mat2a
1417924_AT	pak3
