# Supplementary material for: Effects of sleep and wake on astrocytes: clues from molecular and ultrastructural studies
Source: BMC Biol. 2015 Aug 25;13:66. doi: 10.1186/s12915-015-0176-7 (PMC4548305; doi:10.1186/s12915-015-0176-7)
Supplement: Additional file 7: Table S7. — Astrocyte genes upregulated in SD vs W and W vs SD (fold change >30 %, P < 0.01). (PDF 701 kb) [file 12915_2015_176_MOESM7_ESM.pdf]

Table 7. Astrocyte genes upregulated in SD vs W and W vs SD (fold change>30%, p<0.01)

Note: "X" indicate those genes that were also differentially expressed (up- or downregulated) in the comparison S vs SD, S vs W+SD, and 3m vs 3pm, respectively

Probeset ID	Gene Name	S/SD	S/W-SD	3am/3pm	LogFC	AveExpr	t	adj.P.Val
upregulated in SD								
1435595_at	1810011O10Rik	X			-2.90	3.90	-5.89	0.0017481
1456182_x_at	Mela			X	-2.73	6.32	-8.81	6.40E-05
1424638_at	Cdkn1a	X			-2.29	8.32	-6.25	0.0010507
1421679_a_at	Cdkn1a	X			-2.28	9.00	-5.35	0.0037332
1431501_at	1700037C18Rik	X			-2.19	5.05	-5.19	0.0046996
**1422088_at**	**Mycl1**				-2.11	5.68	-5.41	0.0034251
1434856_at	Ankrd44	X			-2.08	4.66	-6.13	0.0012092
1439632_at	Unknown	X			-2.06	5.65	-7.42	0.0002742
1439072_at	Slc1a3	X			-2.03	6.43	-9.22	4.30E-05
1441206_at	Synpo2	X			-1.99	3.25	-9.88	2.38E-05
1424784_at	Gm13139			X	-1.97	4.96	-6.14	0.0011942
1438750_at	Atrx			X	-1.92	3.27	-6.97	0.0004182
**1437401_at**	**Igf1**				-1.91	4.47	-5.37	0.003608
**1446011_at**	**Unknown**				-1.89	4.03	-4.90	0.0073771
**1431664_at**	**Luc7l**				-1.83	3.46	-7.01	0.0004065
**1436574_at**	**1700029I01Rik**				-1.79	5.17	-7.29	0.0003099
1442025_a_at	Unknown	X			-1.75	7.52	-5.34	0.0037687
1417357_at	Emd	X			-1.72	10.14	-10.92	1.42E-05
1440000_at	E330013P04Rik	X			-1.65	4.48	-6.93	0.0004235
1449143_at	Rtp4			X	-1.65	5.31	-7.24	0.000328
1432913_at	Lrrc52	X			-1.63	3.31	-5.18	0.0047935
1457584_at	AI848100	X			-1.59	3.81	-5.97	0.0015107
1442026_at	Unknown	X			-1.58	7.83	-5.48	0.0031302
1444423_at	Unknown	X			-1.57	3.36	-10.31	1.92E-05
1457871_at	Colec10	X			-1.57	3.49	-9.80	2.40E-05
1437953_at	Gpcpd1	X			-1.55	3.35	-6.94	0.0004235
1424157_at	Ehd2	X			-1.55	6.32	-7.41	0.0002742
1437085_at	D630039A03Rik	X			-1.54	6.79	-4.75	0.0089279
1451453_at	Dapk2	X			-1.54	6.08	-7.07	0.0003851
1453626_at	3930402G23Rik	X			-1.54	5.09	-6.44	0.0008376
1451069_at	Pim3	X			-1.52	8.01	-7.99	0.0001513
1447061_at	Hif3a	X			-1.50	8.50	-4.89	0.0074405
**1429920_at**	**Ifltd1**				-1.48	5.13	-4.79	0.008561
1454354_at	8030476L19Rik	X			-1.45	3.73	-7.54	0.000246
1455056_at	Lmo7	X			-1.41	7.56	-6.37	0.0009077
**1440670_at**	**Unknown**				-1.41	5.37	-5.11	0.0051633
1456101_at	4930403N07Rik	X			-1.39	3.55	-14.15	1.25E-06
**1444866_at**	**E230006M18Rik**				-1.36	4.04	-5.91	0.0016897
1424524_at	Dram1	X			-1.36	4.56	-5.12	0.0051313

1458677_at	Entpd5			X	-1.35	4.66	-5.80	0.0019622
**1426412_at**	**Neurod1**				-1.35	5.65	-5.14	0.0050483
**1433823_at**	**Ptpdc1**				-1.34	4.32	-5.16	0.0048971
1435663_at	Esr1	X	X		-1.32	4.04	-5.32	0.0038809
1443169_at	Unknown	X			-1.31	3.92	-9.66	2.50E-05
1443918_at	2700050L05Rik	X			-1.27	5.32	-6.37	0.0008992
1443071_at	AI839979	X			-1.27	4.14	-9.82	2.40E-05
1432438_at	4930597L12Rik	X			-1.26	3.47	-8.95	5.78E-05
1425281_a_at	Tsc22d3	X			-1.26	10.56	-5.39	0.0035145
1437906_x_at	Txnl1	X		X	-1.26	3.23	-12.05	5.64E-06
1440482_at	Vps13a	X			-1.26	4.32	-8.16	0.0001295
1426734_at	Fam43a	X			-1.26	7.79	-5.41	0.0034251
1436723_at	Cenpi	X			-1.25	2.74	-5.86	0.0018159
1439376_x_at	Dmtf1			X	-1.25	5.06	-6.40	0.0008756
**1441241_at**	**9630013D21Rik**				-1.24	2.86	-6.03	0.0013946
1446521_at	Unknown	X			-1.24	2.92	-11.09	1.42E-05
1432712_at	4933425M03Rik	X			-1.23	4.01	-5.26	0.0041782
**1430217_at**	**Lrguk**				-1.23	3.28	-5.68	0.0023485
1439709_at	Unknown	X		X	-1.23	3.15	-12.90	2.83E-06
**1428504_at**	**Xrcc6**				-1.21	3.88	-7.93	0.0001635
**1437609_at**	**Ube2u**				-1.20	4.10	-4.68	0.0098553
1441279_at	C430002E04Rik	X			-1.19	3.79	-7.21	0.0003341
1426369_at	Far1	X			-1.19	7.60	-4.88	0.0074683
**1434558_at**	**Wdr47**				-1.19	7.96	-6.95	0.0004223
1432501_at	1700092E16Rik	X			-1.18	4.09	-8.91	5.88E-05
1448378_at	Fscn1	X			-1.18	9.98	-6.25	0.0010507
**1423778_at**	**Usp20**				-1.18	5.83	-5.44	0.0032564
1453778_at	2810407C02Rik	X			-1.18	2.88	-10.88	1.42E-05
1444400_at	Rb1	X			-1.18	3.36	-5.34	0.0037637
**1422012_at**	**Crhr2**				-1.17	4.22	-6.01	0.0014411
1446253_at	Unknown	X			-1.17	4.12	-7.54	0.000246
1429009_at	Snrnp70	X			-1.16	4.47	-4.76	0.0088824
**1435266_at**	**Scai**				-1.15	4.05	-6.15	0.0011856
**1435706_at**	**Btbd9**				-1.15	6.32	-5.31	0.0039207
1430069_at	4921517L17Rik	X			-1.14	4.44	-4.77	0.008819
1425638_at	Adap2	X			-1.14	6.65	-7.00	0.0004065
1443366_at	Napg	X			-1.14	3.88	-7.41	0.0002747
**1434376_at**	**Cd44**				-1.13	5.51	-5.45	0.0032345
1455164_at	Arhgap31	X			-1.13	8.05	-4.69	0.0097827
1421172_at	Adam12	X			-1.13	4.90	-5.83	0.0018994
1431046_at	Ppfia3			X	-1.12	6.57	-9.76	2.41E-05
**1455275_at**	**E530001K10Rik**				-1.11	5.60	-4.97	0.0065815
1445182_at	Zfp672			X	-1.11	3.17	-7.88	0.0001635
1446655_at	Scyl3			X	-1.11	5.54	-10.15	1.94E-05
1447224_at	Unknown			X	-1.11	3.49	-7.57	0.0002404
1420150_at	Spsb1	X			-1.10	8.59	-4.88	0.007506
1419519_at	Igf1	X			-1.10	4.81	-4.91	0.0071555

1441578_at	Ccdc86	X		-1.10	5.48	-5.10	0.0052572
**1439477_at**	**Ube2b**			-1.10	3.31	-4.70	0.0096383
1453841_at	2310050P20Rik	X	X	-1.09	2.80	-10.28	1.92E-05
**1445141_at**	**Unknown**			-1.09	3.53	-5.57	0.0028064
**1429661_at**	**Rhobtb3**			-1.08	4.70	-5.67	0.0023793
1445326_at	Sdk1		X	-1.08	4.64	-6.98	0.0004123
1440926_at	Flt1		X	-1.07	4.08	-5.37	0.0036393
**1432601_at**	**Mll5**			-1.07	3.26	-4.90	0.0072832
**1451490_at**	**Lyplal1**			-1.07	7.96	-5.04	0.0058868
**1450605_at**	**Tas2r108**			-1.06	2.98	-6.98	0.0004123
1449752_at	Spsb1	X		-1.06	9.47	-4.75	0.0089279
1418494_at	Ebf2	X		-1.06	4.03	-4.70	0.0096498
1446679_at	Unknown	X		-1.05	3.14	-8.11	0.0001335
**1454392_at**	**9230112J17Rik**			-1.05	4.34	-4.76	0.0088917
1458145_at	Unknown	X		-1.03	4.74	-5.88	0.0017563
1445148_at	Unknown	X		-1.03	4.00	-8.49	9.15E-05
1431969_at	4930402D18Rik	X		-1.02	3.40	-10.21	1.92E-05
**1423192_at**	**Pspc1**			-1.02	8.98	-5.15	0.0049772
1432115_a_at	Pign	X		-1.02	6.19	-7.34	0.0003009
1438793_x_at	Ergic1	X	X	-1.02	3.24	-9.90	2.38E-05
1432137_at	4933434P08Rik	X		-1.01	3.10	-6.28	0.0010026
1431324_at	Ecm2	X		-1.01	3.09	-7.23	0.0003293
1436855_at	Ptprs	X		-1.00	3.00	-7.79	0.0001773
1432424_at	4930505H01Rik		X	-1.00	5.53	-7.23	0.0003293
1441858_at	Unknown	X		-1.00	3.23	-8.14	0.0001298
1425500_x_at	Unknown	X		-1.00	2.83	-7.91	0.0001635
1449516_a_at	Rgs3	X		-1.00	5.95	-6.43	0.0008376
1450883_a_at	Cd36	X		-1.00	3.81	-5.88	0.0017662
**1439962_at**	**2310010J17Rik**			-0.99	8.09	-6.57	0.0006747
1429977_at	9030425L15Rik	X		-0.99	3.32	-9.00	5.75E-05
**1444988_at**	**Unknown**			-0.99	3.45	-6.31	0.0009798
**1457245_at**	**Dirc2**			-0.99	4.23	-5.04	0.0058868
1449802_x_at	Rpusd2	X		-0.98	2.71	-4.79	0.0085301
**1457647_x_at**	**1600023N17Rik**			-0.98	4.69	-5.55	0.002862
1460079_at	Ttc5	X		-0.98	4.27	-5.17	0.0047987
**1421708_a_at**	**Stat6**			-0.98	6.40	-7.24	0.000328
**1441038_at**	**Unknown**			-0.98	3.63	-6.72	0.0005624
1436776_x_at	Slc7a4	X		-0.97	3.13	-6.27	0.0010202
1449037_at	Crem	X		-0.97	8.33	-6.39	0.000891
1426368_at	LOC100044115	X		-0.97	9.75	-5.28	0.0040629
**1459031_at**	**Ttll4**			-0.97	5.92	-5.47	0.0031672
1442544_at	Igh-6		X	-0.97	3.17	-8.58	8.04E-05
**1458827_at**	**Unknown**			-0.96	4.57	-5.70	0.0022708
1455520_at	Ppp2r5c		X	-0.96	5.18	-6.25	0.001051
1447331_at	Unknown	X		-0.96	2.47	-7.05	0.0003933
1440129_at	Aurka	X		-0.96	3.62	-6.79	0.0005096
1443508_at	Dlgap1	X		-0.95	3.07	-8.69	7.21E-05

**1434681_at**	**4932441K18Rik**				-0.94	4.99	-5.27	0.0041069
1419476_at	Adamdec1	X			-0.94	3.16	-7.24	0.000328
**1428458_at**	**Pop1**				-0.94	6.26	-5.48	0.0031155
**1437100_x_at**	**Pim3**				-0.94	10.84	-4.82	0.0082431
1441604_at	Esd	X			-0.94	3.68	-6.30	0.0009848
1457271_at	Cym	X			-0.93	3.72	-7.88	0.0001635
1458478_at	9230110F11Rik	X			-0.93	3.27	-7.53	0.0002465
**1432878_at**	**4930544N03Rik**				-0.93	3.44	-5.98	0.0015107
1418322_at	Crem	X	X		-0.93	7.14	-5.28	0.0040552
1418093_a_at	Egf	X			-0.93	3.83	-9.64	2.50E-05
**1421802_at**	**Ear1**				-0.93	3.11	-6.84	0.0004784
1447269_at	Fam184b	X			-0.93	4.49	-7.42	0.0002742
1418653_at	Cyp2c50	X			-0.93	3.69	-7.00	0.0004065
1425906_a_at	Sema3e	X			-0.92	4.58	-6.04	0.0013785
1427054_s_at	Abi3bp			X	-0.92	3.03	-4.81	0.0082489
1446764_at	Unknown	X			-0.92	3.50	-6.57	0.0006747
1437404_at	Mast4	X			-0.92	6.75	-4.74	0.0090922
1459596_at	Unknown	X			-0.92	4.70	-10.31	1.92E-05
1429180_at	Gmpr2	X			-0.92	3.76	-5.88	0.0017679
1433195_at	9530006O14Rik	X			-0.91	3.72	-8.31	0.0001178
1433126_at	5330422M15Rik	X			-0.91	3.82	-6.43	0.0008376
**1445952_at**	**Unknown**				-0.91	2.62	-7.17	0.0003341
**1447789_x_at**	**Ddx6**				-0.91	3.03	-7.17	0.0003341
1454762_at	Xkrx			X	-0.91	5.42	-6.15	0.0011856
**1441618_at**	**Arhgap29**				-0.91	4.31	-6.71	0.0005677
1446285_at	Unknown	X			-0.90	3.83	-7.82	0.0001751
**1418518_at**	**Furin**				-0.90	8.40	-5.09	0.0053255
**1426370_at**	**Far1**				-0.90	8.36	-5.23	0.0043925
1444800_at	Unknown	X			-0.90	3.97	-8.16	0.0001295
1440658_at	Ammecr1l			X	-0.90	3.32	-5.08	0.0054674
1438596_at	1500017E21Rik	X			-0.90	2.84	-5.28	0.0040629
1448086_at	D1Ertd164e	X			-0.89	3.88	-7.31	0.0003076
**1460134_at**	**9330175E14Rik**				-0.89	3.69	-7.07	0.0003851
**1444680_at**	**Med15**				-0.89	3.97	-6.35	0.0009323
**1453878_at**	**Ubr2**				-0.88	3.83	-4.67	0.0099941
1452158_at	Eprs	X			-0.88	9.50	-6.78	0.0005155
1435901_at	Usp40	X			-0.88	7.42	-5.45	0.0032345
1445343_at	Tmed5	X	X		-0.88	3.87	-5.56	0.0028145
1457789_at	Cln3			X	-0.88	3.87	-5.79	0.0019742
1457258_at	1700017L05Rik	X			-0.87	4.44	-7.89	0.0001635
1444849_at	C76872	X			-0.87	3.66	-6.95	0.0004207
**1443495_at**	**Atp5j2**				-0.87	3.33	-6.08	0.0013035
**1420195_at**	**Unknown**				-0.87	3.36	-6.04	0.0013785
1425973_at	Lyst	X			-0.86	4.11	-6.28	0.0010107
1444642_at	Unknown			X	-0.86	4.26	-7.17	0.0003341
1436527_at	Unknown	X			-0.86	5.27	-4.81	0.0082489
1445238_at	Unknown	X			-0.86	2.92	-5.90	0.0017331

1444757_at	Unknown			X	-0.86	3.11	-6.92	0.0004241
1426713_s_at	Eprs	X			-0.86	9.74	-7.61	0.0002328
1458033_at	Ubr1	X			-0.85	2.53	-8.23	0.0001263
**1444850_at**	**Gm12169**				-0.85	3.85	-5.79	0.0019803
1443090_at	Unknown	X			-0.85	3.28	-8.96	5.78E-05
**1436033_at**	**BC031353**				-0.84	8.42	-6.57	0.0006747
1422033_a_at	Cntf			X	-0.84	6.97	-5.04	0.0058794
1416155_at	Hmgb3			X	-0.84	10.01	-6.12	0.0012327
1426925_at	Rc3h2			X	-0.84	2.91	-5.25	0.0042807
1446172_at	Unknown	X			-0.84	3.87	-6.11	0.0012527
1426371_at	Far1	X			-0.84	7.04	-4.99	0.0063925
1428048_at	LOC269472	X			-0.84	3.07	-6.67	0.0005947
1456134_x_at	Yif1a			X	-0.83	3.51	-9.59	2.59E-05
1454227_at	Htatip2	X			-0.83	3.74	-6.07	0.0013035
1433232_at	4933426I03Rik	X			-0.83	3.06	-9.52	2.79E-05
1438386_x_at	Mat2a	X			-0.83	10.23	-5.57	0.0027851
**1452761_a_at**	**Rbms3**				-0.82	3.80	-5.05	0.0058145
1427541_x_at	Hmmr	X			-0.82	3.72	-6.55	0.0007008
**1418196_at**	**Tep1**				-0.82	6.39	-5.07	0.0055431
1432564_at	1700066C05Rik	X			-0.82	2.68	-6.70	0.0005724
1458556_at	Unknown			X	-0.82	2.81	-9.73	2.41E-05
1458803_at	Slfn9	X			-0.82	5.07	-5.37	0.003608
1421469_a_at	Stat5a				-0.82	7.53	-5.27	0.0041069
1419766_at	Sik1	X	X		-0.81	7.88	-5.34	0.0037637
1419807_at	D4Ertd335e				-0.81	4.00	-4.88	0.007523
1421173_at	Irf4			X	-0.80	3.56	-4.82	0.0082489
**1440791_x_at**	**Tcea2**				-0.80	5.73	-5.33	0.0037814
**1449909_at**	**2010005H15Rik**				-0.80	4.92	-4.69	0.0097814
1429855_at	1700023I07Rik	X			-0.80	3.55	-6.32	0.0009526
1438249_at	Usp7	X			-0.80	2.91	-7.17	0.0003341
1438590_at	Rapgef3	X			-0.80	4.62	-5.85	0.0018426
1440401_at	Unknown	X			-0.80	4.00	-4.90	0.0072853
**1445543_at**	**Zmym5**				-0.79	2.51	-4.77	0.008819
1420109_at	Unknown	X			-0.79	3.94	-6.85	0.0004773
1460417_at	AB041803	X			-0.79	4.05	-7.31	0.0003076
**1444699_at**	**Sf4**				-0.79	3.99	-5.80	0.0019622
1419882_at	Unknown	X			-0.79	2.53	-6.20	0.0011126
**1445693_at**	**Araf**				-0.79	3.57	-4.76	0.0089279
1429086_at	Gm16136	X			-0.78	4.36	-5.52	0.0029829
**1440023_at**	**Pcdh12**				-0.78	3.14	-5.95	0.0015634
1445773_at	Unknown	X			-0.78	3.02	-6.33	0.0009485
**1459810_at**	**1110008F13Rik**				-0.78	4.21	-4.95	0.0068185
**1431767_at**	**4930442G15Rik**				-0.78	4.31	-5.71	0.0022446
1460093_at	Unknown	X			-0.78	3.06	-6.60	0.0006648
**1449102_at**	**Ebf2**				-0.78	2.85	-5.15	0.0049504
**1437982_x_at**	**Cox15**				-0.77	7.42	-4.76	0.0088917
**1458256_at**	**Isy1**				-0.77	3.31	-4.99	0.0063343

1424927_at	Glipr1	X			-0.77	4.03	-4.75	0.0089279
1426955_at	Col18a1	X			-0.77	5.33	-4.82	0.0082485
1443335_at	Unknown	X			-0.77	3.07	-7.21	0.0003341
1447285_at	Unknown	X			-0.77	3.35	-4.70	0.0096383
1452157_at	Eprs	X			-0.77	9.41	-5.48	0.0031126
1455316_x_at	BC094435	X			-0.76	11.29	-5.79	0.0019887
1444043_at	Unknown	X			-0.76	3.17	-5.45	0.0032345
**1438689_at**	**Zfp784**				-0.76	8.12	-5.00	0.0063231
1454577_at	Cdc20b	X	X		-0.76	2.98	-8.74	6.77E-05
1454262_at	Fbxw27			X	-0.76	3.74	-6.96	0.0004203
1449027_at	Rhou	X			-0.76	11.22	-4.98	0.0064637
1459025_at	1700001J04Rik	X			-0.76	3.92	-6.18	0.0011424
1459468_at	C79743	X			-0.75	3.64	-7.30	0.0003076
1432274_at	4930543N07Rik	X			-0.75	3.18	-5.44	0.0032864
**1420193_at**	**Krt17**				-0.75	3.06	-4.76	0.0088917
1417597_at	Cd28	X			-0.75	2.81	-7.00	0.0004065
1459352_at	Unknown	X			-0.75	3.57	-7.30	0.0003076
1430428_at	2310066F23Rik	X			-0.75	2.78	-5.47	0.0031342
**1418892_at**	**Rhoj**				-0.75	9.42	-5.16	0.0048628
**1422529_s_at**	**Casq2**				-0.74	4.29	-6.43	0.0008376
1449777_at	Dus4l	X			-0.74	4.41	-7.49	0.0002539
1431888_s_at	Psg21	X			-0.74	2.86	-5.45	0.0032345
1458972_at	9330112F22Rik	X			-0.74	2.87	-6.43	0.0008376
1456702_x_at	Mat2a	X			-0.74	10.10	-4.74	0.0090959
1430490_at	Unknown	X			-0.74	3.37	-5.53	0.0029816
1432784_at	4930556A17Rik	X			-0.74	3.22	-5.66	0.0024106
1420164_at	D7Ertd183e			X	-0.74	3.48	-7.11	0.0003677
1450222_x_at	Klk1b4	X			-0.74	3.81	-5.86	0.0018156
1440266_at	5830428M24Rik	X			-0.74	3.78	-6.58	0.0006747
1432117_at	4933422A05Rik	X			-0.73	3.43	-6.70	0.0005751
1425559_a_at	Acsm3			X	-0.73	6.14	-5.57	0.0027908
1454126_at	0710001A04Rik	X			-0.73	3.16	-5.16	0.0048628
1447144_at	Unknown	X			-0.73	4.04	-5.16	0.0048628
**1430814_at**	**Cyp2d40**				-0.73	4.03	-5.44	0.0032481
1450597_at	Olfr870			X	-0.73	3.35	-6.16	0.0011762
1422140_at	Gm7609	X			-0.72	2.49	-6.36	0.0009206
**1447757_x_at**	**Inpp5f**				-0.72	6.17	-6.04	0.0013785
1426847_at	Sirt4	X			-0.72	7.01	-5.15	0.0049382
1424410_at	Ttc8			X	-0.72	8.13	-5.26	0.0041805
**1453669_at**	**4930578C19Rik**				-0.72	2.71	-5.05	0.0058109
**1441876_x_at**	**Gm11677**				-0.72	4.95	-5.25	0.0042476
**1456526_at**	**C130034I24Rik**				-0.71	3.06	-5.12	0.0051633
1433034_at	2310007H11Rik	X			-0.71	3.81	-6.98	0.0004123
**1441865_at**	**Unknown**				-0.71	3.20	-5.98	0.0015107
1440398_at	Unknown	X			-0.71	3.73	-5.21	0.0045367
1438489_at	Smn1	X			-0.71	3.76	-7.43	0.0002735
1459265_at	Myo19	X			-0.71	3.81	-6.47	0.0007931

**1459937_at**	**Ass1**				-0.71	3.60	-5.12	0.005129
**1428297_at**	**Map4k2**				-0.71	5.58	-5.69	0.0023401
**1446648_at**	**Unknown**				-0.71	2.83	-5.28	0.0040552
**1437951_at**	**Dis3l2**				-0.71	3.42	-5.98	0.0015107
**1459782_x_at**	**Abcc5**				-0.71	4.19	-5.49	0.0030659
**1455595_at**	**Ugt2b36**				-0.70	3.34	-4.87	0.0076401
1429812_at	2610002D18Rik	X			-0.70	4.36	-6.19	0.0011142
1459932_at	D8Ertd28e	X	X		-0.70	3.28	-7.31	0.0003076
1423306_at	2010002N04Rik	X			-0.70	10.80	-4.88	0.0075506
**1447282_at**	**Gng13**				-0.70	5.17	-6.21	0.0011041
1423226_at	Ms4a1	X			-0.69	3.13	-5.83	0.0018775
1440235_at	Itga10	X			-0.69	3.13	-5.15	0.0049375
1430215_at	2610020H08Rik	X			-0.69	4.10	-4.79	0.0085301
**1452231_x_at**	**Mndal**				-0.69	2.40	-4.68	0.0099462
1421556_at	Serpina3a	X			-0.69	3.34	-5.26	0.0041889
1444023_at	Ank2			X	-0.69	2.45	-5.20	0.0045926
1425782_at	Plcb1	X			-0.69	3.16	-5.88	0.0017695
1431392_at	5730596B20Rik	X			-0.69	3.64	-7.08	0.0003851
1453695_at	Vmn1r90	X			-0.69	4.65	-6.18	0.0011368
1445931_at	Unknown	X			-0.69	3.30	-5.29	0.0040552
**1431215_at**	**Dnajc6**				-0.68	2.77	-4.71	0.0093922
1449919_at	Krtap6-2	X			-0.68	3.92	-5.72	0.0021958
1436652_at	5830418K08Rik	X			-0.68	2.77	-5.86	0.0018061
1431819_at	Unknown			X	-0.68	5.76	-4.73	0.0092122
1442693_at	Trim5	X			-0.68	2.90	-6.99	0.0004123
1432683_at	5530400K19Rik	X			-0.68	4.37	-7.02	0.0004065
**1445134_at**	**Mkl2**				-0.67	8.96	-5.76	0.0021038
1438708_x_at	Ywhab	X			-0.67	2.96	-7.89	0.0001635
**1442456_at**	**Spata5**				-0.67	2.69	-5.64	0.0024863
1445943_at	Unknown	X			-0.67	3.24	-5.48	0.0031044
1432672_at	4933407A17Rik	X	X	X	-0.67	3.69	-5.55	0.0028604
1431282_at	Med24	X			-0.67	3.99	-6.81	0.0004967
1433776_at	Lhfp	X			-0.66	9.87	-5.06	0.0057513
**1426141_at**	**Mrgpra1**				-0.66	2.82	-4.91	0.007271
1450807_at	Ltb4r2	X	X		-0.66	4.33	-5.39	0.0035145
1454563_at	4930573C08Rik	X			-0.66	3.45	-6.16	0.0011725
1430264_at	2610030P05Rik	X			-0.66	3.38	-5.58	0.0027707
**1449639_at**	**Unknown**				-0.65	2.34	-8.26	0.0001263
1421707_at	Tmc2	X			-0.65	2.92	-7.74	0.0001902
1415852_at	Impdh2	X			-0.65	8.92	-5.30	0.0039915
**1450947_at**	**2610528J11Rik**				-0.65	2.84	-6.65	0.0006242
1457312_at	Unknown	X			-0.65	4.78	-5.45	0.0032345
1442855_at	Dopey1	X	X		-0.64	4.12	-5.64	0.0024921
1439386_x_at	Mat2a	X	X		-0.64	9.54	-5.37	0.0036057
1420424_at	1700054O13Rik	X			-0.64	3.47	-6.20	0.0011041
1438586_at	Tbx22	X			-0.64	2.19	-5.67	0.0023694
1444362_at	Neurod2	X			-0.64	4.24	-5.48	0.0031044

1440211_at	Cyp2j11	X			-0.64	3.34	-6.09	0.0012769
**1445043_at**	**Unknown**				-0.64	3.25	-6.07	0.0013047
1447492_at	Unknown			X	-0.63	3.67	-5.35	0.0037428
1460069_at	Smc6	X			-0.63	2.59	-5.68	0.0023458
**1425596_at**	**AI317395**				-0.63	2.57	-4.97	0.0065953
1457596_at	Unknown	X			-0.63	3.01	-4.82	0.0082431
1440935_at	Unknown			X	-0.63	4.48	-6.76	0.0005295
1444445_at	C77648	X			-0.63	3.50	-6.00	0.0014542
1445376_at	Unknown	X			-0.63	3.57	-4.88	0.0074683
**1443764_x_at**	**Rab27b**				-0.63	5.02	-4.98	0.0064998
1418550_x_at	Defa-rs1	X			-0.63	6.07	-4.97	0.0065953
1452486_a_at	Cryaa	X			-0.63	6.06	-5.02	0.0061157
**1443234_at**	**BC088983**				-0.63	3.14	-6.16	0.0011762
**1446405_at**	**Myst3**				-0.63	2.77	-4.91	0.0071555
1430182_a_at	1700006J14Rik			X	-0.63	2.75	-5.35	0.0037332
1427893_a_at	Pmvk	X			-0.62	11.76	-5.90	0.00171
1432795_at	2310058F05Rik	X			-0.62	2.73	-4.81	0.0082489
1440114_x_at	Kank3			X	-0.62	3.71	-5.63	0.0025246
1450550_at	Il5	X			-0.62	2.48	-7.49	0.0002539
1444775_at	Gpr107	X			-0.62	3.68	-4.83	0.0081351
**1454502_at**	**2900022B07Rik**				-0.62	3.34	-6.51	0.0007421
1434741_at	Rreb1	X	X		-0.62	3.88	-5.68	0.0023458
1447512_at	Unknown	X			-0.62	3.32	-6.30	0.0009848
1428589_at	Mrpl41	X			-0.62	11.95	-4.74	0.0090377
1447799_x_at	1700001P01Rik	X			-0.62	3.22	-6.39	0.000891
1428471_at	Sorbs1	X			-0.62	11.16	-4.99	0.0063343
**1426064_at**	**Cyp3a44**				-0.61	2.34	-6.15	0.0011836
1437425_at	Gdap1			X	-0.61	4.85	-6.24	0.0010628
1428588_a_at	Mrpl41	X			-0.61	12.17	-6.24	0.0010564
1442259_at	Unknown	X			-0.61	2.90	-4.85	0.007988
**1449154_at**	**Col11a1**				-0.61	2.48	-4.73	0.0092543
**1458895_at**	**Naa11**				-0.61	3.33	-4.93	0.0070017
**1442897_at**	**Fam13b**				-0.60	3.06	-5.43	0.003318
1459150_at	Unknown	X			-0.60	2.50	-7.05	0.0003933
1459491_at	D12Ertd673e	X			-0.60	4.02	-4.78	0.008669
**1444782_at**	**4930417H01Rik**				-0.60	3.95	-4.76	0.0089279
**1445869_at**	**Unknown**				-0.60	2.20	-4.97	0.0065831
**1451855_at**	**Bcl7a**				-0.60	2.73	-5.81	0.0019417
**1456450_at**	**Unknown**				-0.60	3.24	-5.13	0.005129
1447990_at	C76332	X			-0.60	3.09	-5.39	0.0035218
1421629_at	Gabre	X			-0.59	3.58	-4.84	0.0080095
**1443296_at**	**Cdk16**				-0.59	4.09	-4.78	0.008669
1442864_at	Esrra	X			-0.59	4.48	-4.78	0.0087047
1445947_at	Unknown	X			-0.59	2.47	-6.07	0.0013035
1426435_at	Tmem135	X			-0.59	9.27	-5.63	0.002544
1425933_a_at	Nt5c2	X			-0.59	9.22	-5.29	0.0040453
1458942_at	C230037E05Rik	X			-0.59	2.75	-5.41	0.0034579

1429166_s_at	Clmn	X			-0.58	6.71	-4.96	0.0066182
1447286_at	Unknown	X			-0.58	2.56	-5.39	0.0035145
**1459926_at**	**C77068**				-0.58	2.32	-4.69	0.0097058
**1447639_x_at**	**Unknown**				-0.57	2.34	-5.31	0.0039344
**1446389_at**	**Unknown**				-0.57	3.05	-5.74	0.0021492
1449159_at	Gnb3	X			-0.57	5.48	-5.35	0.0037332
**1421372_at**	**Klk1b4**				-0.57	3.45	-4.79	0.0085247
1443301_at	Unknown	X			-0.56	2.18	-6.59	0.0006711
**1451708_at**	**Gpr33**				-0.56	3.16	-5.10	0.0052869
**1430916_at**	**Fancd2**				-0.56	3.14	-6.21	0.0011041
1459097_at	Unknown			X	-0.56	3.38	-5.19	0.0046707
**1444672_at**	**Unknown**				-0.56	2.13	-5.29	0.0040552
**1459358_at**	**Unknown**				-0.56	4.19	-4.84	0.0080138
**1433298_at**	**5830461L22Rik**				-0.56	3.00	-5.12	0.0051313
1421770_a_at	Tlx2	X			-0.56	5.73	-5.12	0.005129
**1422928_at**	**Elane**				-0.55	2.87	-4.84	0.0080138
1459225_at	Gnl3l			X	-0.55	2.69	-6.07	0.0013035
1447241_at	Unknown	X			-0.55	5.02	-5.83	0.0018775
1456313_x_at	Mrpl28	X	X		-0.55	4.60	-5.79	0.0019803
**1431077_at**	**Alpi**				-0.53	3.20	-4.75	0.0089279
1418645_at	Hal	X			-0.53	4.28	-4.82	0.0082431
1447598_x_at	Polr1a	X			-0.52	2.02	-5.51	0.0030064
**1444619_x_at**	**Psmb8**				-0.52	3.00	-4.68	0.0098536
1432064_at	Ubox5	X			-0.52	3.38	-5.51	0.0030068
1433049_at	5730478J17Rik	X			-0.52	2.41	-6.22	0.0010938
1420161_at	AA409749			X	-0.52	4.11	-6.03	0.0013842
1433438_x_at	Mela	X			-0.51	2.75	-5.55	0.0028604
1447599_x_at	Epn2	X			-0.51	3.47	-5.31	0.0039207
**1446417_at**	**Unknown**				-0.51	2.16	-4.81	0.0082712
1426308_at	Mbd3l1	X			-0.51	3.79	-4.82	0.0082431
1429991_at	Fezf1			X	-0.51	4.07	-5.35	0.0037332
1435889_at	Mark2	X			-0.50	9.68	-4.75	0.0089279
1446491_at	Unknown	X			-0.50	3.35	-5.18	0.0047944
1446683_at	Eps15l1	X			-0.50	5.41	-4.94	0.006861
**1422280_at**	**Gzmk**				-0.50	3.46	-4.94	0.0068433
**1431845_at**	**Gm4640**				-0.50	3.14	-4.86	0.007779
1440577_at	Unknown	X	X		-0.50	3.78	-5.12	0.005129
**1420206_at**	**Unknown**				-0.50	2.59	-5.20	0.0045926
1421253_at	Nrap	X	X		-0.49	6.38	-4.80	0.0083937
1459138_at	Unknown	X			-0.49	2.58	-4.68	0.0099322
1427793_at	Unknown	X			-0.49	4.23	-4.75	0.0089279
1449322_at	Gm13363	X			-0.49	10.57	-4.69	0.0097407
**1445993_at**	**Unknown**				-0.49	2.66	-5.67	0.0023728
1433789_at	Snhg3	X			-0.49	4.80	-4.71	0.0095593
1445187_at	9430070O13Rik			X	-0.48	2.60	-4.69	0.0097023
1449992_at	Prss29	X			-0.48	5.32	-4.94	0.0068433
1458804_at	Unknown	X			-0.48	2.74	-5.40	0.0035145

1417299_at	Nek2	X			-0.47	3.10	-4.69	0.0097407
1437603_at	BC050777	X	X		-0.47	3.61	-5.28	0.0040552
**1454435_at**	**Unknown**				-0.46	4.99	-4.75	0.008978
1426221_at	Vwa5a	X			-0.46	8.34	-4.68	0.0098204
	upregulated in W							
1423147_at	Mat1a			X	0.44	3.38	4.96	0.0067016
1458495_at	Unknown	X	X	X	0.46	4.20	4.74	0.0090516
**1448684_at**	**Ppp1r2**				0.49	9.80	5.17	0.0048299
1455838_at	Slfnl1			X	0.52	4.99	4.72	0.009312
**1431410_at**	**D16Ertd472e**				0.52	4.47	4.83	0.0081831
1432281_a_at	Itgb6	X	X	X	0.53	2.94	5.56	0.0028493
1454129_at	4933416M06Rik			X	0.53	4.67	4.84	0.0081036
1447245_at	Unknown			X	0.54	4.19	4.96	0.0066194
1433513_x_at	Ndufa12	X			0.54	12.44	6.10	0.0012696
1444747_at	Unknown			X	0.54	2.76	5.87	0.0017796
1444536_at	Unknown			X	0.54	3.65	4.70	0.0095861
1450578_at	Sry			X	0.54	3.14	4.89	0.0074134
**1432030_at**	**4930434J06Rik**				0.55	2.99	5.38	0.0035892
1424493_s_at	Ugt3a1			X	0.56	4.08	5.61	0.0026134
**1433245_at**	**6720475M21Rik**				0.56	3.79	5.51	0.0030064
1429796_at	Kalrn	X			0.56	9.71	5.45	0.0032345
1449081_at	Ces3			X	0.58	2.57	6.24	0.0010628
1459735_at	Fkbp15			X	0.58	4.03	4.82	0.0082485
1442629_at	Unknown			X	0.58	3.61	4.78	0.0086254
1435825_at	Acvrl1	X			0.58	4.39	4.91	0.0071555
1432751_at	4930403O18Rik			X	0.59	3.59	5.04	0.0058868
1451534_at	Scgn			X	0.60	4.47	4.82	0.0082431
**1432074_at**	**4930517E11Rik**				0.61	3.81	4.75	0.0089279
**1421481_at**	**Unknown**				0.61	3.63	4.79	0.0085968
**1458739_at**	**Unknown**				0.61	2.96	5.34	0.0037814
1420061_s_at	AA675344			X	0.62	2.64	5.09	0.0053747
1422276_at	P2ry4	X			0.62	3.16	5.05	0.0058121
1442405_at	Csrp2bp			X	0.62	3.62	5.68	0.0023458
1441289_at	Unknown	X			0.64	2.69	5.11	0.0051633
1441103_at	Unknown	X			0.65	3.66	5.80	0.0019598
1440124_at	B230334C09Rik			X	0.65	3.42	5.16	0.0048945
1446274_at	Slc16a1	X	X		0.66	3.34	5.05	0.0057606
**1442999_at**	**B930036G03Rik**				0.66	9.95	5.98	0.0015107
1420784_at	Scn11a			X	0.66	3.84	4.77	0.0087352
**1439694_at**	**Fbxw16**				0.66	4.35	5.29	0.0040453
1446027_at	Unknown			X	0.67	4.45	5.37	0.0036393
1429858_at	1700011E24Rik			X	0.67	4.39	6.61	0.0006637
1443121_at	Calcoco1			X	0.67	4.47	5.24	0.0043136
1446813_s_at	D5Ertd560e			X	0.68	3.09	7.04	0.0003936
1444932_at	4732419C18Rik			X	0.68	4.83	4.67	0.0099657
1441177_at	Unknown	X			0.68	3.86	6.21	0.0010947

1442146_at	Unknown	X			0.68	3.96	6.58	0.0006747
1442467_at	Nav2			X	0.68	3.90	5.58	0.0027707
1458058_at	7030407E18Rik			X	0.68	3.76	5.01	0.0061553
1431908_at	4933408J17Rik	X			0.69	4.04	5.30	0.004031
**1455974_at**	**1110049F12Rik**				0.69	2.96	5.51	0.0030068
**1429821_at**	**2810046L04Rik**				0.69	2.89	5.17	0.0048333
1459892_at	Psg19			X	0.69	2.57	6.68	0.0005907
1437326_x_at	Cela3b	X	X	X	0.69	3.86	6.34	0.0009408
1438779_at	Col4a3			X	0.70	3.44	5.50	0.0030193
1418286_a_at	Efnb1			X	0.70	6.97	4.73	0.0092219
**1453443_at**	**1110015O18Rik**				0.70	2.64	4.72	0.009312
1436932_at	Grhl3			X	0.70	3.55	4.76	0.0089204
1443025_at	Unknown			X	0.70	3.31	4.99	0.0064308
1432578_at	Naif1			X	0.71	5.89	6.30	0.0009848
1442485_at	Unknown			X	0.71	3.45	7.11	0.0003663
**1427361_at**	**Hoxc6**				0.71	4.83	5.24	0.0043136
**1452402_at**	**Unknown**				0.72	2.66	5.52	0.0029829
1446788_at	A430091L06Rik	X			0.72	3.49	5.38	0.0035997
**1443426_at**	**Sltm**				0.72	2.75	6.87	0.0004633
1447134_at	Unknown			X	0.72	3.59	5.51	0.0030064
1441155_at	Unknown			X	0.72	3.24	6.30	0.0009848
1442573_at	Unknown			X	0.72	3.22	6.95	0.0004207
1451951_at	Igkv14-111			X	0.73	3.84	7.46	0.0002631
**1441794_at**	**Unknown**				0.74	3.41	4.74	0.00908
1423714_at	Asf1b			X	0.74	5.10	5.01	0.0061397
**1447325_at**	**Unknown**				0.74	4.75	5.82	0.0019038
1447642_x_at	Dmwd			X	0.75	3.13	5.73	0.0021931
1430696_at	Eif2ak1	X			0.76	4.90	7.70	0.0001985
**1458610_at**	**Unknown**				0.76	3.58	6.56	0.0006886
**1428802_at**	**Mgat3**				0.76	4.73	5.51	0.0030064
1422802_at	Defa3			X	0.76	3.77	5.55	0.002862
1425289_a_at	Cr2	X			0.77	4.07	5.29	0.0040552
**1430098_at**	**6330409D20Rik**				0.77	4.46	5.18	0.0047944
1449866_at	Syt2			X	0.78	4.90	5.27	0.0041275
**1441435_at**	**Unknown**				0.78	3.29	4.89	0.0074134
**1456539_at**	**Unknown**				0.78	2.45	5.12	0.0051633
1454291_at	4933428P19Rik	X			0.79	3.91	8.20	0.0001278
1432626_at	5730507A11Rik			X	0.79	4.00	7.80	0.0001773
1432500_at	2410006F04Rik			X	0.79	2.93	7.27	0.0003198
1430299_at	Tsc22d4			X	0.79	6.75	4.95	0.006763
1420991_at	Ankrd1	X	X	X	0.80	3.67	7.20	0.0003341
**1454736_at**	**Ankrd57**				0.80	7.73	4.91	0.0071555
1445149_at	Pvr			X	0.80	4.11	6.87	0.0004608
1430653_at	A930026B05Rik			X	0.81	4.61	5.97	0.0015224
**1446188_at**	**Cep97**				0.81	3.69	7.47	0.0002617
1427756_x_at	Igh-6			X	0.81	3.68	5.39	0.0035145
1447080_at	Aco1			X	0.81	4.50	5.60	0.0026882

**1432852_at**	**Phactr1**			0.82	3.29	5.50	0.0030276
**1419589_at**	**Cd93**			0.82	3.16	4.94	0.0068607
1446628_at	Unknown		X	0.83	4.08	8.08	0.000136
**1440813_s_at**	**Plxnb3**			0.84	4.60	5.89	0.0017466
**1430706_at**	**Pebp4**			0.84	2.67	7.37	0.0002897
**1447437_at**	**Unknown**			0.85	3.15	6.93	0.0004235
1449572_at	Trhr		X	0.85	3.24	4.92	0.0070464
1429611_at	1700034E13Rik	X		0.85	5.20	7.18	0.0003341
1433224_at	5830442K09Rik	X		0.85	3.33	7.57	0.0002404
1417852_x_at	Clca1		X	0.85	2.81	7.79	0.0001773
1447128_at	C330013J21Rik	X		0.85	4.05	6.35	0.0009249
1454788_at	Arl4c	X		0.86	8.96	6.61	0.0006637
1429402_at	Glt8d2		X	0.86	5.41	4.82	0.0082485
1439929_at	Unknown		X	0.87	2.96	8.16	0.0001295
**1430145_at**	**Lrrc28**			0.87	3.99	4.79	0.008561
1442081_at	Gm4632		X	0.87	4.51	6.75	0.0005323
1444354_at	Unknown		X	0.87	4.69	8.66	7.43E-05
1442331_at	Unknown		X	0.88	3.72	8.01	0.0001497
**1419179_at**	**Txnl4a**			0.89	8.62	5.14	0.0050483
1446751_s_at	Impact		X	0.89	6.55	5.81	0.0019351
1431564_at	Bcar3		X	0.89	3.60	5.73	0.0021931
1417878_at	E2f1		X	0.89	6.82	4.72	0.009335
1447851_x_at	Atp10a		X	0.90	4.62	5.50	0.0030193
1441441_at	Prrg1		X	0.91	3.33	5.57	0.0027888
1419717_at	Sema3e		X	0.91	3.23	4.90	0.0072832
**1445164_at**	**Unknown**			0.92	2.82	5.24	0.0043136
**1446047_at**	**Unknown**			0.93	4.31	6.88	0.0004555
1445433_at	Glt8d2	X		0.93	3.05	4.83	0.0081442
**1454305_at**	**Cbx3**			0.94	4.19	5.51	0.0030064
**1458202_at**	**Fam65b**			0.95	5.97	5.10	0.0052623
1437563_at	Phf20l1	X		0.95	4.89	4.98	0.0064998
**1421009_at**	**Rsad2**			0.96	3.78	4.75	0.0089279
1419075_s_at	Saa1	X		0.96	4.03	7.19	0.0003341
1431038_at	Rassf4		X	0.97	3.34	5.59	0.0027165
1444457_at	Unknown	X		0.97	5.27	5.40	0.0035039
**1446899_at**	**Unknown**			0.98	2.87	5.04	0.0058868
**1441669_at**	**Acap2**			0.98	4.38	4.75	0.0089279
1423473_at	Sept2		X	0.98	2.96	5.77	0.0020269
**1421601_at**	**Gsx2**			0.98	3.21	4.72	0.009298
**1431306_at**	**Nt5c3**			0.99	3.55	6.15	0.0011856
**1458374_at**	**C79407**			0.99	2.84	6.39	0.000891
1458755_at	Unknown		X	0.99	3.04	7.06	0.000388
1431263_at	Prkag2		X	0.99	3.09	7.51	0.0002499
**1441690_at**	**Cdh8**			0.99	2.65	4.83	0.0082021
1422942_at	Galr2		X	0.99	3.97	5.83	0.0018775
1440048_at	Smarca5		X	1.00	3.19	6.72	0.0005608
1440711_at	C630001G18Rik		X	1.01	3.22	6.58	0.0006747

1442228_at	Unknown	X		X	1.02	3.45	10.26	1.92E-05
1445236_at	Unknown	X			1.02	3.83	8.44	9.69E-05
**1429468_at**	**1110018F16Rik**				1.03	7.32	5.80	0.0019569
1445309_at	Unknown	X			1.04	5.06	6.83	0.0004807
**1437063_at**	**Fem1a**				1.04	7.43	5.18	0.0047461
**1426016_a_at**	**Tro**				1.04	6.45	4.78	0.0086254
1437124_at	A630052C17Rik	X			1.04	6.00	5.31	0.0039132
1441395_at	AU021933	X			1.05	4.21	6.12	0.0012327
1417877_at	Eepd1	X			1.05	7.46	6.33	0.0009471
**1425291_at**	**Foxj1**				1.06	7.07	4.93	0.0069444
**1448983_at**	**Cdrt4**				1.07	5.30	5.36	0.0036397
**1455528_at**	**Unknown**				1.08	5.73	4.85	0.0078842
**1450956_at**	**LOC100048724**				1.08	6.16	5.01	0.006175
**1442030_at**	**Unknown**				1.08	4.17	6.38	0.0008924
**1421303_at**	**Ikzf1**				1.09	5.19	4.81	0.0083337
1444308_at	Unknown			X	1.09	4.78	5.74	0.0021513
**1458404_at**	**Ndufb8**				1.09	5.51	4.74	0.0091095
1430436_at	Fam115a			X	1.10	3.09	6.78	0.0005155
1435560_at	Itgal	X	X	X	1.10	3.85	6.79	0.0005096
1427497_at	2610015P09Rik			X	1.10	4.38	8.77	6.63E-05
1421686_at	Npvf			X	1.11	4.43	9.11	4.97E-05
**1456998_at**	**Dbpht2**				1.11	4.21	4.83	0.0082191
**1430212_at**	**Zfp712**				1.11	3.23	4.81	0.0082675
**1418603_at**	**Avpr1a**				1.11	4.26	5.45	0.0032345
1446175_at	Unknown			X	1.12	3.48	8.90	5.88E-05
**1446119_at**	**Unknown**				1.13	4.81	5.55	0.002862
1431397_at	3110099E03Rik	X			1.15	4.08	8.07	0.000136
1457093_at	Gapvd1			X	1.15	3.58	7.98	0.0001535
1448577_x_at	Syngr2			X	1.15	7.39	5.61	0.0026134
**1436002_at**	**Scube3**				1.15	4.92	6.11	0.0012527
**1434762_at**	**Orai2**				1.15	5.74	4.85	0.0079963
1454361_at	9230106L01Rik			X	1.16	3.28	6.81	0.0005003
**1429284_at**	**Mobkl2b**				1.16	5.43	6.23	0.0010628
1450260_at	Grpr	X			1.16	4.51	5.45	0.0032474
**1429250_at**	**Dync2h1**				1.16	4.69	4.68	0.0099388
1456681_at	Ptchd1			X	1.17	6.05	6.49	0.0007813
1437370_at	Sgol2			X	1.17	4.15	5.01	0.0061553
1435794_at	BC050254			X	1.18	5.84	5.75	0.0021243
1423367_at	Wnt7a	X			1.18	8.80	6.03	0.0013932
1441074_at	Unknown			X	1.19	4.40	7.74	0.0001902
1430330_at	Wiz			X	1.20	3.53	6.07	0.0013035
1444163_at	Nek5	X			1.21	3.99	4.73	0.0092254
**1436633_at**	**Unknown**				1.22	7.02	5.10	0.0052869
1427618_at	Cdh9			X	1.23	3.61	6.43	0.0008376
**1435116_at**	**4933403G14Rik**				1.23	5.21	4.96	0.0067016
1444207_at	Alms1			X	1.24	3.63	5.20	0.0046301
1453395_at	5330403D14Rik			X	1.24	3.82	8.22	0.0001263

1445653_at	BC031361	X		1.24	4.05	7.92	0.0001635
1446785_at	Unknown	X		1.25	3.30	5.14	0.0050483
1438515_at	Zfp207		X	1.25	4.67	5.24	0.0043136
1430464_at	9430021M05Rik	X		1.25	5.82	5.51	0.0030064
1431362_a_at	Smoc2		X	1.27	4.61	6.29	0.0009877
**1436107_at**	**Naa38**			1.27	5.90	5.28	0.0040629
1460073_at	Unknown		X	1.29	3.90	6.57	0.000683
1445505_at	Ndst1		X	1.29	3.59	5.71	0.0022589
1459716_at	AI835735	X		1.29	5.54	5.32	0.0038815
**1429443_at**	**Cpne4**			1.31	5.42	4.89	0.0074446
1443707_at	2900046B09Rik	X		1.34	4.04	6.47	0.0007931
1459255_at	Unknown		X	1.35	3.17	7.19	0.0003341
1460744_at	Unknown	X		1.36	5.08	7.35	0.0002989
**1422889_at**	**Pcdh18**			1.37	3.46	4.95	0.006763
1440862_at	Unknown		X	1.39	4.48	5.65	0.0024596
1432268_at	2310068J16Rik	X		1.42	5.94	5.51	0.0030068
1447178_at	Unknown	X		1.43	4.65	5.93	0.0016195
1459618_at	Unknown	X		1.43	3.99	8.62	7.67E-05
**1420938_at**	**Hs6st2**			1.44	6.34	4.83	0.0081877
1422530_at	Prph		X	1.45	4.06	5.58	0.002759
1444269_at	Unknown		X	1.49	3.68	8.11	0.0001335
1447552_s_at	Unknown		X	1.53	3.24	5.24	0.0043083
**1426033_at**	**Rgs9**			1.54	4.47	4.81	0.0082489
1422907_at	Gnat2	X		1.55	3.92	8.88	5.88E-05
1441311_at	Rps6ka2	X		1.56	4.42	6.75	0.0005323
1447268_at	Nol12	X		1.57	4.62	7.04	0.0003933
1458620_at	Gm5129		X	1.60	5.38	6.85	0.0004773
1456729_x_at	Rtel1	X		1.60	4.25	7.60	0.0002338
1425669_at	Mobkl2b	X	X	1.61	3.94	9.92	2.38E-05
1434070_at	Jag1	X		1.61	6.26	5.12	0.0051633
1446761_at	D8Ertd56e		X	1.68	3.33	8.23	0.0001263
**1445763_at**	**1700013F07Rik**			1.73	4.78	6.38	0.0008992
1444559_at	Phtf2		X	1.79	4.06	6.59	0.0006711
**1441389_at**	**Unknown**			1.81	3.45	7.18	0.0003341
**1443246_at**	**Fastkd2**			1.81	5.13	7.85	0.0001703
1456670_at	A930007A09Rik	X		1.82	4.06	7.54	0.000246
**1436540_at**	**Mirlet7d**			2.03	4.92	6.94	0.0004235
1429955_at	5031434O11Rik	X	X	2.03	4.48	10.41	1.92E-05
**1429616_at**	**Zfp91**			2.07	5.24	6.44	0.0008367
**1442708_at**	**Unknown**			2.11	4.62	5.14	0.0049998
