# Supplementary figures and images for: Effects of sleep and wake on astrocytes: clues from molecular and ultrastructural studies
Source: BMC Biol. 2015 Aug 25;13:66. doi: 10.1186/s12915-015-0176-7 (PMC4548305; doi:10.1186/s12915-015-0176-7)

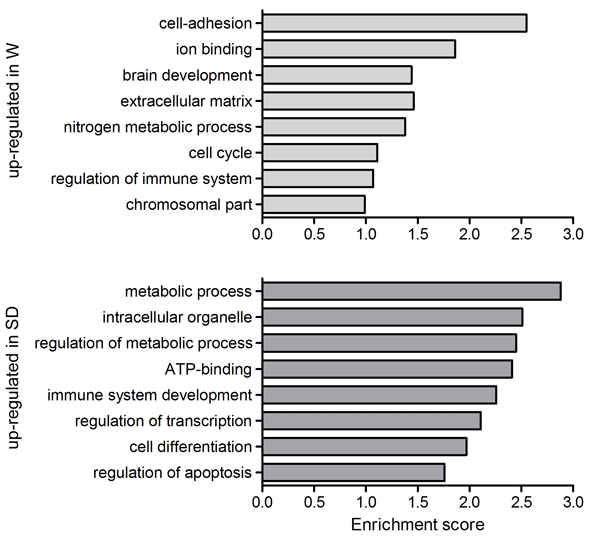

Supplement: Additional file 8: Figure S1. — Functional characterization of genes differentially expressed in wake (W) and sleep deprivation (SD). Functional annotation analysis (DAVID, default settings, except for kappa = 4, similarly threshold = 0.75) for W (n = 200) and SD (n = 384) genes. The top ten functional annotation clusters in order of enrichment score are shown for W (top) and SD (bottom). (TIFF 137 kb) [file 12915_2015_176_MOESM8_ESM.tiff]

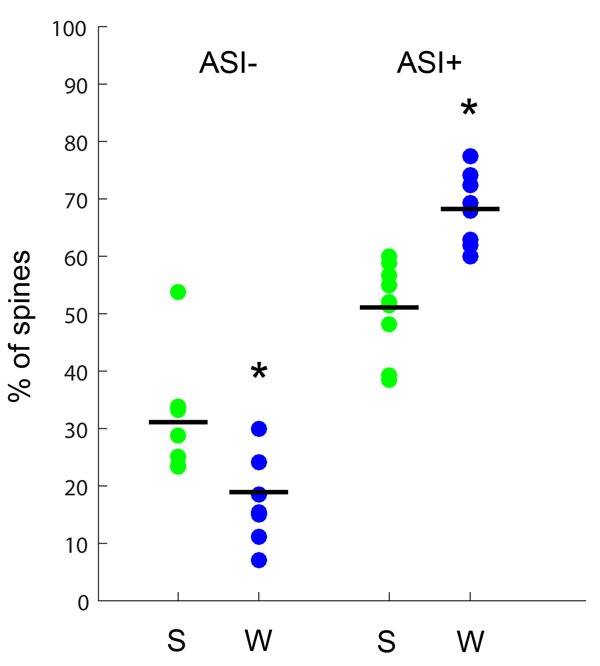

Supplement: Additional file 10: Figure S2. — PAPs move closer to the synaptic cleft after spontaneous wake. Percentage of PAP+ASI– and PAP+ASI+ spines per dendrite in layer II cortical fields for S (n = 9 dendrites; PAP+ASI–, 31.1 ± 9.6 %; PAP+ASI+, 51.09 ± 7.88 %), W (n = 8 dendrites; PAP+ASI–, 18.2 ± 8.45 %; PAP+ASI+, 68.23 ± 6.27 %). PAP+ASI–, S vs W, MW, *P = 0.019; PAP+ASI+, S vs W, MW, *P = 0.0002. All values are mean ± standard deviation. (TIFF 52 kb) [file 12915_2015_176_MOESM10_ESM.tiff]
